# Click-designed vanilloid-triazole conjugates as dual inhibitors of AChE and Aβ aggregation†

**DOI:** 10.1039/d2ra07539c

**Published:** 2023-01-19

**Authors:** Marwa Elsbaey, Yasuhiro Igarashi, Mahmoud A. A. Ibrahim, Eman Elattar

**Affiliations:** a Pharmacognosy Department, Faculty of Pharmacy, Mansoura University Mansoura 35516 Egypt marwaelsebay1611@mans.edu.eg; b Biotechnology Research Center and Department of Biotechnology, Toyama Prefectural University 5180 Kurokawa, Imizu Toyama 939-0398 Japan; c Computational Chemistry Laboratory, Chemistry Department, Faculty of Science, Minia University 61519 Egypt; d School of Health Sciences, University of KwaZulu-Natal Westville Durban 4000 South Africa

## Abstract

Based on their reported neuroprotective properties, vanilloids provide a good starting point for the synthesis of anti-Alzheimer's disease (AD) agents. In this context, nine new 1,2,3-triazole conjugates of vanilloids were synthesized *via* click chemistry. The compounds were tested for their effect on acetylcholine esterase (AChE) and amyloid-beta peptide (Aβ) aggregation. The triazole esters (*E*)-(1-(4-hydroxy-3-methoxybenzyl)-1*H*-1,2,3-triazol-4-yl)methyl 3-(4-hydroxy-3 methoxyphenyl)acrylate 9 and (1-(4-hydroxy-3-methoxybenzyl)-1*H*-1,2,3-triazol-4-yl)methyl-4-hydroxy-3-methoxybenzoate 8 displayed dual inhibitory activity for AChE and Aβ aggregation with IC_50_ values of 0.47/0.31 μM and 1.2/0.95 μM, respectively, as compared to donepezil (0.27 μM) and tacrine (0.41 μM), respectively. The results showed that the triazole ester moiety is more favorable for the activity than the triazole ether moiety. This could be attributed to the longer length of the spacer between the two vanillyl moieties in the triazole esters. Furthermore, the binding affinities and modes of the triazole esters 9 and 8 were examined against AChE and Aβ utilizing a combination of docking predictions and molecular dynamics (MD) simulations. Docking computations revealed promising binding affinity of triazole esters 9 and 8 as potential AChE, Aβ40, and Aβ42 inhibitors with docking scores of −10.4 and −9.4 kcal mol^−1^, −5.8 and −4.7 kcal mol^−1^, and −3.3 and −2.9 kcal mol^−1^, respectively. The stability and binding energies of triazole esters 9 and 8 complexed with AChE, Aβ40, and Aβ42 were measured and compared to donepezil and tacrine over 100 ns MD simulations. According to the estimated binding energies, compounds 9 and 8 displayed good binding affinities with AChE, Aβ42, and Aβ40 with average Δ*G*_binding_ values of −32.9 and −31.8 kcal mol^−1^, −12.0 and −10.5 kcal mol^−1^, and −20.4 and −16.6 kcal mol^−1^, respectively. Post-MD analyses demonstrated high steadiness for compounds 9 and 8 with AChE and Aβ during the 100 ns MD course. This work suggests the triazole conjugate of vanilloids as a promising skeleton for developing multi-target potential AD therapeutics.

## Abstract 2

Based on their reported neuroprotective properties, vanilloids provide a good starting point for the synthesis of anti-Alzheimer's disease agents.

## Graphical abstract

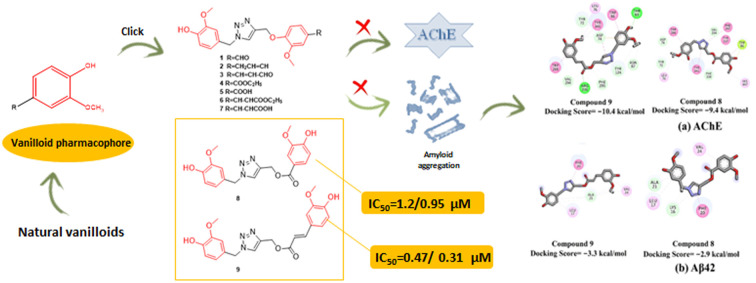

## 1. Introduction

Alzheimer's disease (AD) is a progressive neurodegenerative disorder and is the most prevalent cause of dementia.^1^ It affects more than 40 million people worldwide.^2^ Even though the pathophysiology of AD has not been completely elucidated, cholinergic deficiency and amyloid-β peptide (Aβ) deposition are widely accepted as important features of the pathophysiology of AD,^3^ the pathological aggregates of Aβ deposits being known as senile plaques.^4^ Acetylcholine esterase (AChE) has been reported to consistently co-localize with Aβ deposits and induce their assembly by forming a complex with the growing fibrils.^4^

The enzyme AChE is involved in the hydrolysis of the neurotransmitter acetylcholine (ACh). The profound loss of forebrain cholinergic neurons during the progression of AD, results in a progressive decline in acetylcholine. Current therapies are mostly based on AChE inhibitors (AChEI) to reverse the cholinergic deficit.^5^ Dual targeted inhibitors of AChE and Aβ aggregation are the main focus of AD paradigm.^6^ These drugs can be synthesized or harvested from nature, the advantage of the latter being the potential for chemical diversity, biological selectivity and favorable properties. Natural products and their derivatives represent more than 50% of the market pharmaceutics.^7^

Vanilloids are a group of natural products that are characterized by the presence of a vanillyl group. They include vanillin, vanillic acid, eugenol, capsaicin *etc.* Vanilloids attracted the attention of the authors because of their reported neuroprotective properties. Vanillin is reported to inhibit both Aβ aggregation and AChE and its profound antioxidant activity in neuroblastoma cells.^8^ Eugenol,^9^ vanillic acid,^10^ ferulic acid,^11^ curcumin, and capsaicin^12^ are reported to decrease or inhibit AChE. Vanillin,^13^ eugenol,^14^ ferulic acid, its derivatives,^15^ curcumin^16^ and capsaicin^17^ are also reported to inhibit Aβ aggregation. Vanillin derivatives have been reported as multi-target drugs for AD treatment.^8,18^ Several ferulic acid and its analogs based scaffolds were developed for management of AD.^19,20^ Consequently, the authors have selected the vanilloid pharmacophore to design potential anti-AD drugs. The triazole ring is reported as a good linker to combine pharmacophores into innovative bioactive functional molecules.^21^ Furthermore, several triazole-based compounds were reported as promising inhibitors against AD.^22–25^ In this context, we are encouraged to design new 1,2,3-triazole-conjugates of vanilloids and investigate their neuroprotective activity against AD. The molecular docking technique was utilized to anticipate the docking scores and poses of the most potent triazole esters 9 and 8 with AChE and Aβ40/42. The docked structures of compounds 9 and 8 complexed with AChE and Aβ40/42 were then subjected to MD over the simulation time of 100 ns. Structural and energetical analyses were utilized to examine the constancy of compounds 9 and 8 complexed with the investigated targets over 100 ns MD.

## 2. Results and discussion

### 2.1. Chemistry

**Fig. 1:**
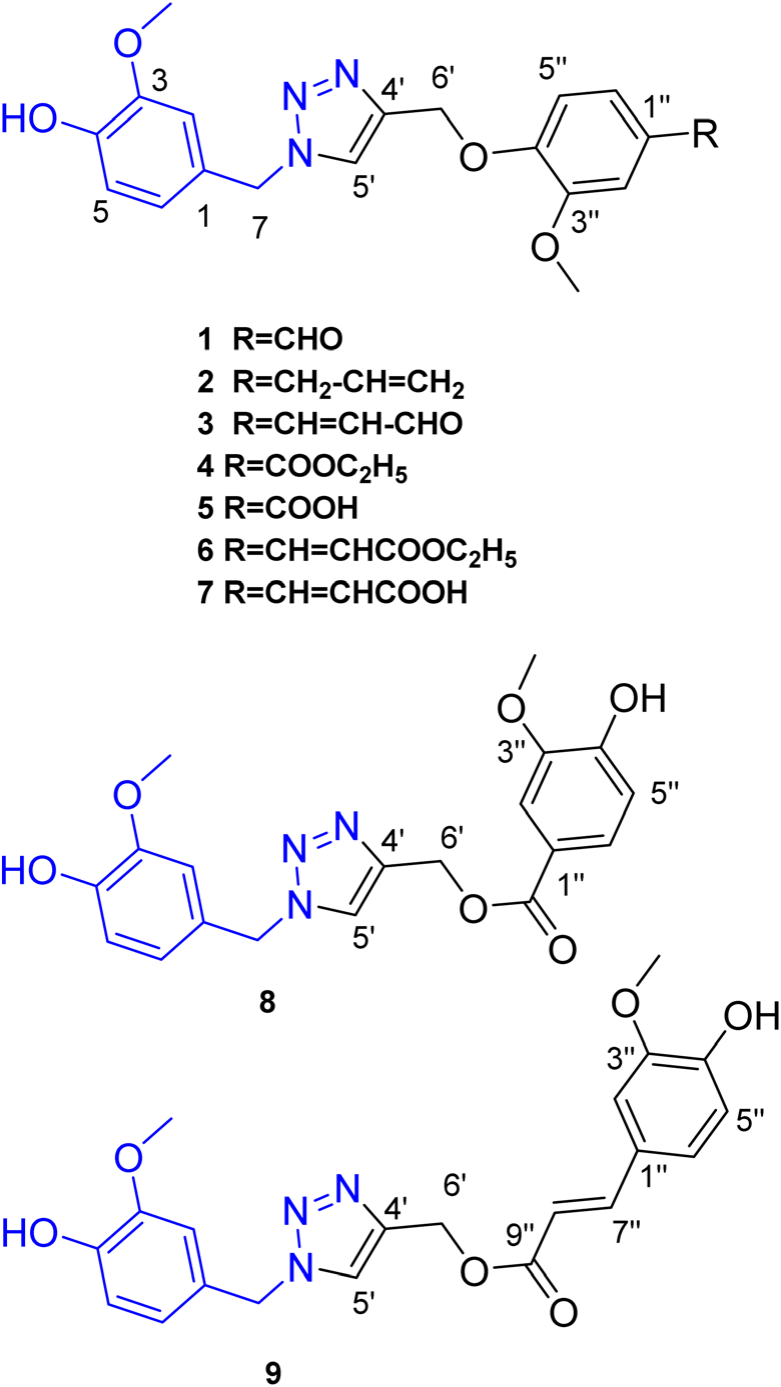
Compounds 1–9 prepared *via* click reaction.

**Scheme 1:**
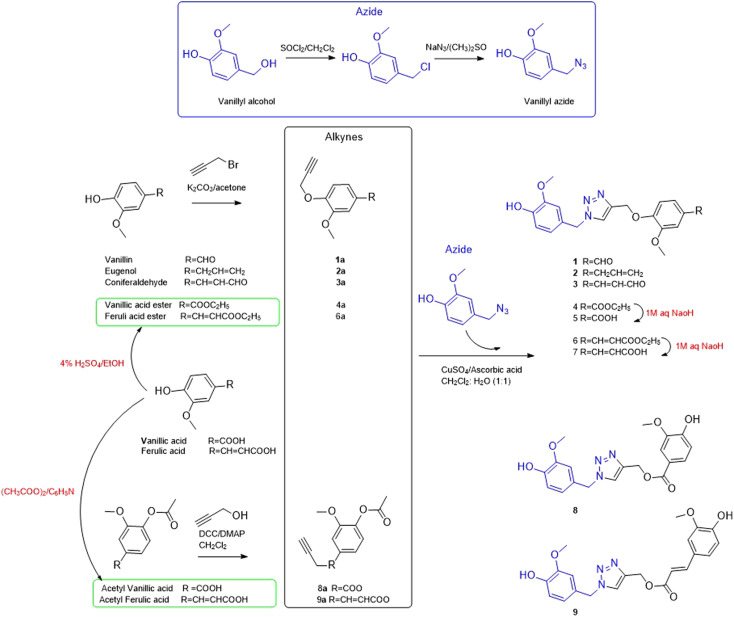
General route of synthesis for triazole derivatives (1–9).

Based on the reported anti-Alzheimer properties of both of vanilloids and triazole-based compounds, the authors set a rationale for synthesis of triazole-conjugates of vanilloids *via* click chemistry. Nine new 1,2,3-triazole-conjugates of vanilloids (Fig. 1) were prepared *via* click chemistry (Scheme 1).

For azide, vanillyl alcohol were purchased from Sigma-Aldrich. For alkynes, ferulic acid, and vanillin were purchased from Sigma-Aldrich. Coniferaldehyde and vanillic acid were previously isolated from *Cocos nucifera* L.^26^ Eugenol was extracted and purified from clove oil using 30% aqueous KOH, according to the literature.^27^ Vanillyl alcohol was converted to vanillyl azide and reacted with different alkynes. The monoalkynes were prepared from natural vanilloids, as will be described in the experimental part.

Compounds 1–3 were obtained *via* click reaction of vanillyl azide with the respective mono-alkyne ether. Compounds 4 and 6 were obtained *via* click reaction of vanillyl azide with the respective mono-alkyne ether after masking the carboxylic moiety with an ester. Compounds 5 and 7 were obtained from the ester hydrolysis of compounds 4 and 6, respectively. Compounds 8 and 9 were obtained *via* click reaction of vanillyl azide with the mono-alkyne ester after masking the hydroxyl group.

Investigation of the ^1^H-NMR Table 1 and APT Table 2 spectral data of the compounds confirmed the formation of a triazole moiety in each compound. The triazole moiety was characterized in ^1^H-NMR spectrum by the presence of the methine proton signal at *δ*_H_ ranging from 7.60 to 8.27, in addition to the methylene protons (–CH_2_O–) and (–CH_2_N–) at *δ*_H_ ranging from 5.19 to 5.40 and 5.35 to 5.48, respectively.

**Table 1:** H^1^-NMR data (*δ*_H_, m, *J* in Hz) of compounds 1–9 (400 MHz)

H	1	2^a^	3^a^	4	5	6	7	8^a^	9
**Azide moiety**									
2	6.99, brs	6.74, brs	6.69, brs	6.99, brs	6.98, brs	7.00, brs	6.98, brs	6.76 (1.8)	6.98, brs
5	6.73–6.79	6.88 (8)	6.83 (8)	6.74–6.77	6.77	6.76–6.79	6.76	6.88 (8)	6.76–6.77
6	6.73–6.79	6.78 (8, 1.8)	6.75 (2.0, 8)	6.74–6.77	6.77	6.76–6.79	6.76	6.80 (8, 1.8)	6.76–6.77
7	5.46, 2H, s	5.38, 2H, s	5.35, 2H, s	5.46, 2H, s	5.48, 2H, s	5.45, 2H, s	5.46, 2H, s	5.40, 2H, s	5.45, 2H, s
OCH_3_	3.74, s	3.78, 3H, s	3.75, 3H, s	3.74, 3H, s	3.74, 3H, s	3.74, 3H, s	3.73, 3H, s	3.78, 3H, s	3.74, 3H, s
OH	9.14	—	—	9.15	9.14	—	—		9.14

**Triazole moiety**									
5′	8.27, s	7.53, s	7.52, s	8.25, s	8.25, s	8.19, s	8.23, s	7.60, s	8.19, s
6′	5.23, 2H, s	5.20, 2H, s	5.23, 2H, s	5.19, 2H, s	5.20, 2H, s	5.21, 2H, s	5.14, 2H, s	5.40, 2H, s	5.21, 2H, s

**Alkyne moiety**									
2′′	7.37–7.39	6.69^b^	6.99, brs	7.44 (1.8)	7.44, brs	7.32, brs	7.31, brs	7.5 (1.8)	7.32, brs
5′′	7.37–7.39	6.92 (8.2)	7.10 (8.3)	7.57 (8.0, 1.8)	7.55 (8.2)	7.2^b^	7.16–7.31	7.57 (8.0, 1.8)	7.14^b^
6′′	7.54 (8.2, 1.5)	6.66–6.69^b^	7.06^b^	7.26 (8)	7.24 (8.2)	6.76–6.79^b^	7.16–7.31	6.89 (8)	7.12^b^
7′′	9.84, s	3.3 (6.7)	7.32 (15.8)	—	—	7.55 (16)	7.52 (16)	—	7.56 (15.9)
8′′	—	5.92, m	6.53 (7.7, 15.8)	—	—	6.47 (16)	6.45 (16)	—	6.48 (15.9)
9′′	—	5.06, m	9.57 (7.7)	—	—	—	—	—	—
OCH_3_	3.79, 3H, s	3.79, 3H, s	3.80, 3H, s	3.77, 3H, s	3.76, 3H, s	3.79, 3H, s	3.76, 3H, s	3.79, 3H, s	3.79, 3H, s
CH_2_	—	—	—	4.27, 2H (7.1)	—	4.17, 2H (7.1)	—		
CH_3_	—	—	—	1.3, 3H (7.1)	—	1.2, 3H, (7.1)	—		
COOH	—				12.7		—	—	—

a Is recorded in CDCl_3_. The rest of compounds are recorded in DMSO-*d*_6_.

b Overlapped.

**Table 2:** APT-NMR data of compounds 1–9 (100 MHz)

	1	2^a^	3^a^	4	5	6	7	8^a^	9
**Azide moiety**									
1	126.5, qC	126.1, qC	126.1, qC	126.5, qC	126.5, qC	126.6, qC	126.5, qC	126.0, qC	126.6, qC
2	112.6, CH	112.3, CH	110.9, CH	112.6, CH	112.6, CH	112.7, CH	112.6, CH	110.9, CH	112.7, CH
3	147.6, qC	147.2, qC	147.2, qC	147.7, qC	147.6, qC	147.7, qC	147.6, qC	147.2, qC	147.7, qC
4	146.7, qC	146.4, qC	146.4, qC	146.7, qC	146.7, qC	146.7, qC	146.7, qC	146.4, qC	146.7, qC
5	115.5, CH	114.8, CH	114.8, CH	115.5, CH	115.5, CH	115.4, CH	115.5, CH	114.9, CH	115.5, CH
6	121.1, CH	121.6, CH	121.8, CH	121.1, CH	121.1, CH	121.1, CH	121.1, CH	121.7, CH	121.2, CH
7	53.0, CH_2_	54.2, CH_2_	54.5, CH_2_	52.9, CH_2_	52.9, CH_2_	53.0, CH_2_	52.9, CH_2_	54.3, CH_2_	52.9, CH_2_
OCH_3_	55.6, CH_3_	55.9, CH_3_	56.09, CH_3_	55.6, CH_3_	55.6, CH_3_	55.62, CH_3_	55.6, CH_3_	56.09, CH_3_	55.7, CH_3_

**Triazole moiety**									
4′	142.1, qC	144.8, qC	144.2, qC	142.3, qC	142.4, qC	142.6, qC	142.6, qC	143.4, qC	142.3, qC
5′	124.8, CH	122.7, CH	123.2, CH	124.7, CH	124.7, CH	124.6, CH	124.6, CH	124.5, CH	124.6, CH
6′	61.7, CH_2_	63.4, CH_2_	63.0, CH_2_	61.6, CH_2_	61.6, CH_2_	61.6, CH_2_	61.6, CH_2_	57.7, CH_2_	57.0, CH_2_

**Alkyne moiety**									
1′′	129.9, qC	133.9, qC	127.8, qC	122.4, qC	123.4, qC	127.4, qC	127.6, qC	121.5, qC	125.5, qC
2′′	109.6, CH	110.9, CH	110.3, CH	111.8, CH	112.1, CH	110.6, CH	110.5, CH	112.0, CH	111.2, CH
3′′	149.3, qC	149.5, qC	149.8, qC	148.6 qC	148.5 qC	149.2, qC	149.2, qC	148.0, qC	148.0, qC
4′′	152.8, qC	145.9, qC	150.6, qC	151.5, qC	151.3, qC	149.6, qC	149.4, qC	150.5, qC	149.5, qC
5′′	112.5, CH	114.5, CH	113.7, CH	112.5, CH	112.4, CH	115.7, CH	115.5, CH	114.3, CH	115.6, CH
6′′	125.9, CH	120.5, CH	123.4, CH	122.9, CH	123.0, CH	122.7, CH	122.4, CH	125.7, CH	123.3, CH
7′′	191.5, CH	39.9, CH_2_	152.8, CH	165.5, qC	167.1, qC	144.5, CH	143.9, CH	166.3, qC	145.6, CH
8′′	—	137.5, CH	127.1, CH	—	—	113.4, CH	113.1, CH	—	114.0, CH
9′′	—	115.8, CH_2_	193.7, CH	—	—	166.6, qC	168.0, qC	—	166.4, qC
OCH_3_	55.5, CH_3_	55.8, CH_3_	56.05, CH_3_	55.5, CH_3_	55.4, CH_3_	55.58, CH_3_	55.5, CH_3_	55.8, CH_3_	55.6, CH_3_
CH_2_	—	—	—	60.5, CH_2_	—	59.9, CH_2_	—	—	—
CH_3_	—	—	—	14.3, CH_3_	—	14.0, CH_3_	—	—	—

a Recorded in CDCl_3_. The rest of compounds are recorded in DMSO-*d*_6_.

Compound 1 was obtained *via* click reaction of vanillyl azide with mono-alkyne vanillin as colorless needles, yielding 16.2%. Its molecular formula was determined to be C_19_H_19_N_3_O_5_ from [M − H]^−^, [M + H]^+^ and [M + Na]^+^ at *m*/*z* 368.1248, 370.1365, and 392.1185, respectively, (calc. 368.1246, 370.1403 and 392.1222) in the HRMS (Fig. S3 and S4†). The ^1^H-NMR spectrum (Fig. S1†) revealed the presence of an aldehydic proton; a downfield proton; six aromatic protons, two methylene signals and two methoxy groups. The three aromatic protons at *δ*_H_ 6.99 (H-2) and 6.73–6.79 (H-5/6); the methylene at *δ*_H_ 5.46 (H-7) and the methoxy groups at *δ*_H_ 3.74 (3H, s) were assigned to the vanillyl moiety of the azide. This was confirmed by the APT signals at *δ*_C_ 147.6, 146.7, 126.5, 121.1, 115.5, 55.6, and 53.0. Meanwhile, the aldehyde proton at *δ*_H_ 9.84 (H-7′′), the remaining three aromatic protons at *δ*_H_ 3.37–7.39 (H-2′′/5′′) and 7.54 (H-6′′), and the methoxy group at *δ*_H_ 3.79 (3H, s) were assigned to the vanillin moiety from the alkyne. This was confirmed from the APT signals at *δ*_C_ 191.5, 152.8, 149.3, 129.9, 125.9, 112.5, 109.6 and 55.5. These data were compatible with those published for the vanillyl alcohol^28^ and vanillin^29^ moieties. The methylene at *δ*_H_ 5.23 (H-6′), the methine at *δ*_H_ 8.27 (H-5′), and the APT signals at *δ*_C_ 142.1, 124.8, and 61.7 were typical for triazole moiety.^30^ Hence it was concluded to be the new compound, 4-((1-(3-methoxy-4-hydroxybenzyl)-1*H*-1,2,3-triazol-4-yl)methoxy)-3-methoxybenzaldehyde.

Compound 2 was obtained *via* click reaction of vanillyl azide with mono-alkyne eugenol as brown amorphous residue, yield 19.0%. Its molecular formula was determined to be C_21_H_23_N_3_O_4_ from [M + H]^+^ and [M + Na]^+^ at *m*/*z* 382.1774 and 404.1589, respectively, (calc. 382.1767 and 404.1589) in the HRMS (Fig. S8†). The NMR data were similar for compound 1 except for replacing vanillin signals with eugenol signals. The eugenol moiety was characterized by the aromatic signals at *δ*_H_ 6.69, 6.92, and 6.66–6.69 and the allyl moiety at *δ*_H_ 5.92, 5.06, 3.3. Hence it was concluded to be the new compound, 4-((4-((4-allyl-2-methoxyphenoxy)methyl)-1*H*-1,2,3-triazol-1-yl)methyl)-2-methoxyphenol.

Compound 3 was obtained *via* click reaction of vanillyl azide with mono-alkyne coniferaldehyde as yellow amorphous residue, yielding 33.2%. Its molecular formula was determined to be C_21_H_21_N_3_O_5_ from [M + H]^+^ and [M + Na]^+^ at *m*/*z* 396.1501 and 418.1323, respectively, (calc. 396.1559 and 418.1379) in the HRMS (Fig. S12†); [M − H]^−^ at *m*/*z* 394.1410 (calc. 394.1403) (Fig. S13†). The coniferaldehyde moiety was characterized by the aromatic signals at *δ*_H_ 7.10, 7.06, and 6.99; the olefinic protons at *δ*_H_ 7.32 and 6.53; and the aldehydic proton at *δ*_H_ 9.57. Hence it was concluded to be the new compound, 4-((1-(3-methoxy-4-hydroxybenzyl)-1*H*-1,2,3-triazol-4-yl)methoxy)-3-methoxyphenyl acrylaldehyde.

Compounds 4 and 8 were obtained *via* click reaction of vanillyl azide with the mono-alkyne ether of vanillic acid ester and the mono-alkyne ester of acetyl vanillic acid, respectively, as a white powder, yielding 63.8%; and white residue, yielding 21.5%.

Compound 4 was assigned to the molecular formula C_21_H_23_N_3_O_6_ from [M − H]^−^ at *m*/*z* 412.1511 (calc. 412.1509) in the HRMS. The vanillic acid ester moiety was characterized by the aromatic signals at *δ*_H_ 7.57, 7.44, and 7.26 and the ethyl moiety at *δ*_H_ 4.27 (2H, q) and 1.30 (2H, t). It was identified as 4-((1-(3-methoxy-4-hydroxybenzyl)-1*H*-1,2,3-triazol-4-yl)methoxy)-3-methoxy-ethyl benzoate.

Compound 5 was obtained from the ester hydrolysis of compound 4, as white amorphous powder, yielding 95.2%. Compounds 5 and its corresponding ethyl ester 4 showed the same NMR data except for the presence of the characteristic signals of the ethyl moiety at *δ*_H_ 4.27 (2H, q) and 1.3 (2H, t). For the APT spectrum, the free carboxylic group in compound 5 resonated at a higher field at *δ*_C_ 167.1 compared to the corresponding ethyl ester 4, where it resonated at *δ*_C_ 165.5. The same pattern was observed for compound 7 and its corresponding ethyl ester 6. Compound 5 was identified as 4-((1-(3-methoxy-4-hydroxybenzyl)-1*H*-1,2,3-triazol-4-yl)methoxy)-3-methoxybenzoic acid.

The isomeric compounds 5 and 8 were assigned to the molecular formula C_19_H_19_N_3_O_6_ based on the molecular ion peak at 408.1170 and 408.1178, respectively. The triazole ester 8 showed a distinct upfield shift in C-6′ (*δ*_C_ 57.7) and C-7′′ (*δ*_C_ 166.3) as compared to the triazole ether 5 (*δ*_C_ 61.6) and (*δ*_C_ 167.1). Also, the chemical shift of the vanillic acid moiety was slightly different between the two compounds. The same pattern was observed for the isomeric compounds 7 and 9, where C-6′ and C-7′′resonated at *δ*_C_ 61.6/57.0 and 168.0/166.4, respectively. It was named (1-(4-hydroxy-3-methoxybenzyl)-1*H*-1,2,3-triazol-4-yl)methyl 4-hydroxy-3-methoxy benzoate.

Similarly, compounds 6 and 9 were obtained *via* click reaction of vanillyl azide with the mono-alkyne ether of ferulic acid ester and the mono-alkyne ester of acetyl ferulic acid, respectively, as a white powder, yielding 56.8%; and white powder yielded 68.0%.

Compound 6 was assigned to the molecular formula C_23_H_25_N_3_O_6_ from [M − H]^−^ at *m*/*z* 438.1664, (calc. 438.1665) in the HRMS. The ferulic acid ester moiety was characterized by the aromatic protons at *δ*_H_ 7.32, 7.2, 6.76–6.79; the olefinic protons at *δ*_H_ 7.55 and 6.47; and the methoxy group at *δ*_H_ 3.79. It was named as ethyl 4-((1-(3-methoxy-4-hydroxybenzyl)-1*H*-1,2,3-triazol-4-yl)methoxy)-3-methoxyphenyl acrylate.

Compound 7 was obtained from the ester hydrolysis of compound 6, as white amorphous powder, yielding 43.4%. Both compounds displayed the same NMR data except for the presence of the characteristic signals of the ethyl moiety at *δ*_H_ 4.17 (2H, q), 1.2 (2H, t), and *δ*_C_ 59.9, 14.0. For the APT spectrum, the free carboxylic group in compound 7 resonated at a higher field at *δ*_C_ 168.0 as compared to the corresponding ethyl ester 6, where it resonated at *δ*_C_ 166.6. It was named as 4-((1-(3-methoxy-4-hydroxybenzyl)-1*H*-1,2,3-triazol-4-yl)methoxy)-3-methoxyphenyl acrylic acid.

The isomeric compounds 7 and 9 were assigned to the molecular formula C_21_H_21_N_3_O_6_ based on the molecular ion peak at 410.1438 and 410.1361 (calc. 410.1352), respectively. The triazole ester 9 showed a distinct upfield shift in C-6′ (*δ*_C_ 57.0) and C-9′ (*δ*_C_ 166.4) as compared to the triazole ether 7 (*δ*_C_ 61.6) and C-9′′(*δ*_C_ 168.0). It was named as (*E*)-(1-(4-hydroxy-3-methoxybenzyl)-1*H*-1,2,3-triazol-4-yl)methyl 3-(4-hydroxy-3 methoxyphenyl)acrylate.

### 2.2. Biological evaluation

The semi-synthetic compounds (1–9) and curcumin were evaluated for their inhibitory effect on acetylcholine esterase and β-amyloid aggregation Table 3. Curcumin was chosen because it is a natural vanilloid analogue with reported AChE and Aβ aggregation inhibition activity.

**Table 3:** AchE and Aβ42 inhibitory activity^a^

Compound	IC_50_ ± SD (μM)	
	AChE	Aβ42 inhibition
1	14.24 ± 0.72	4.85 ± 0.24
2	26.83 ± 1.35	22.74 ± 1.14
3	5.53 ± 0.28	2.86 ± 0.14
4	2.107 ± 0.12	1.59 ± 0.08
5	2.109 ± 0.11	1.48 ± 0.07
6	2.52 ± 0.13	3.06 ± 0.15
7	10.55 ± 0.53	4.48 ± 0.22
8	1.2 ± 0.06	0.95 ± 0.05
9	0.47 ± 0.02	0.31 ± 0.02
Curcumin	8.72 ± 0.44	13.39 ± 0.67
Donepezil	0.28 ± 0.01	—
Tacrine	—	0.41 ± 0.02

a The data are expressed as mean ± standard deviation.

Regarding the acetylcholine esterase assay results, compound 9 was the most active, IC_50_ value of 0.47 ± 0.02 μM; compounds 8, 4, 5, 6, and 3 showed IC_50_ values ranging from 1.2 ± 0.06 to 5.53 ± 0.28 μM.

Compound 9 was the most active, showing an IC_50_ value of 0.47 ± 0.02 μM, which is about two times that of the standard donepezil, the IC_50_ value of 0.27 ± 0.01 μM. It is worth noting that its isomeric compound 7 was much less active, the IC_50_ value of 10.55 ± 0.53 μM. This may suggest that the triazole ester moiety is more favorable to the activity than the triazole ether moiety. This may be also confirmed by observing the IC_50_ values of the isomeric compounds 5 and 8; the triazole ester 8 displayed about half of the IC_50_ value of its isomeric triazole ether 5, IC_50_ value 1.2 ± 0.06 and 2.11 ± 0.11 μM, respectively.

Compound 4 showed nearly similar activity to compound 5, suggesting that a free or conjugated carboxylic group may have not impact on the activity. However, this pattern was not observed for 7 and its ethyl ester 6 compound 4 showed nearly similar activity to compound 5, suggesting that a free or conjugated carboxylic group may have no impact on the activity. However, this pattern was not observed for 7 and its ethyl ester 6; the ethyl ester derivative 6 displayed about the fifth of IC_50_ of the free form 7, IC_50_ value 2.52 ± 0.13 and 10.55 ± 0.53 μM, respectively. It is worth noting that compounds 9, 8, 4, 5, 6 and 3 were more active than curcumin, the IC_50_ value of 8.72 ± 0.1 μM. Compounds 7, 1, and 2 were much less active than curcumin showing the IC_50_ value of 10.55 ± 0.53, 14.24 ± 0.72 and 26.83 ± 1.35 μM. The hybrid containing the vanillin and the eugenol moiety was the least active.

The ethyl ester derivative 6 displayed about fifth of IC_50_ of the free form 7, the IC_50_ value of 2.52 ± 0.13 and 10.55 ± 0.53 μM, respectively. It is worth noting that compounds 9, 8, 4, 5, 6, and 3 were more active than curcumin, the IC_50_ value of 8.72 ± 0.1 μM. Compounds 7, 1, and 2 were much less active than curcumin showing the IC_50_ value of 10.55 ± 0.53, 14.24 ± 0.72, and 26.83 ± 1.35 μM. The hybrid containing the vanillin and the eugenol moiety was the least active.

For the amyloid-β aggregation assay, compound 9 was more active than tacrine; their IC_50_ values were 0.31 ± 0.02 and 0.41 ± 0.02 μM, respectively. Compound 8 was next in activity with the IC_50_ values of 0.95 ± 0.05 μM. It is worth noting that the triazole esters 9 and 8 were much more active than their isomeric triazole ethers 7 and 5, respectively. This may suggest that the triazole ester moiety is more favorable to the activity. Compound 5 and its ethyl ester 4 also showed comparable IC_50_ values of 1.48 ± 0.07 and 1.59 ± 0.08 μM, respectively. For compound 7 and its ethyl ester 6, they showed nearly similar IC_50_ values of 4.48 ± 0.22 and 3.06 ± 0.15 μM, respectively. Next in activity to compounds 5 and 4 was compound 3, showing the IC_50_ value of 2.86 ± 0.14 μM.

All compounds except for compound 2 were more active than curcumin, the IC_50_ value of 22.74 ± 1.14 and 13.39 ± 0.67 μM, respectively. The hybrid containing the eugenol moiety was the least active.

From the above results, it can be concluded that compounds 9 and 8 could act as dual inhibitors for AChE and Aβ aggregation with IC_50_ values 0.47/1.2 and 0.31/0.95 μM, respectively. Their promising activity over compounds 1–7, could be attributed to the longer length of the spacer between the two vanillyl moieties. Therefore, they hold a particular interest in developing new anti-Alzheimer drugs. The skeleton of 9 and 8 may offer some structural features for the development of dual inhibitors. Hence, they should be subjected to further investigation for designing novel anti-Alzheimer drugs. The results provides a preliminary idea about the anti-AD potential of vanilloid-triazole conjugates. However, a further extensive study is required to investigate their activity *in vitro* and *in vivo*, including the morphology of the Aβ-oligomers.

### 2.3. Molecular docking

**Fig. 2:**
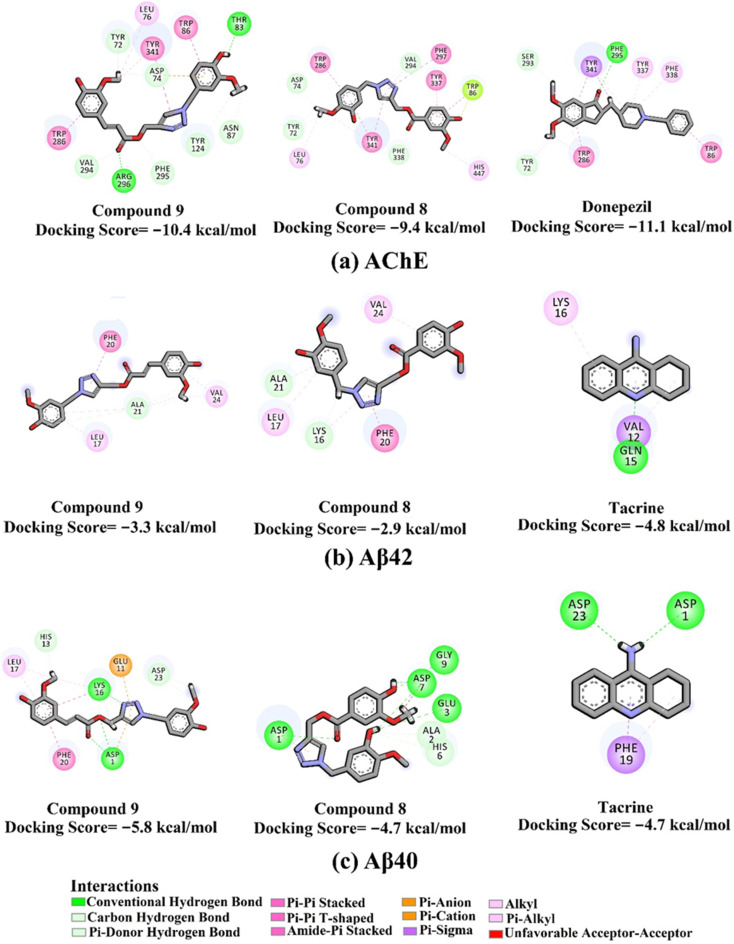
2D molecular interactions of triazole esters 9 and 8 and controls with (a) AChE (PDB ID: 4EY7), (b) Aβ42 (PDB ID: 1IYT), and (c) Aβ40 (PDB ID: 1BA4).

The docking scores and poses of triazole esters 9 and 8 with AChE and Aβ40/42 were predicted using AutoDock4.2.6 software. The protended binding features and docking scores are shown in Fig. 2. As depicted in Fig. 2, triazole esters 9 and 8 unveiled good docking scores towards AChE and Aβ40 with values of −10.4 and −5.8 kcal mol^−1^ and −9.4 and −4.7 kcal mol^−1^, respectively. Triazole esters 9 and 8 were also investigated against the Aβ42 protein. For Aβ42, the docking scores were not promising compared to those against Aβ40, with values of −3.3 and −2.9 kcal mol^−1^ of compounds 9 and 8, respectively (Fig. 2). The good potentiality of compounds 9 and 8 may be imputed to their ability to form a variation of H-bonds, π-based, and other interactions with the key residues within the binding sites of AChE and Aβ40. More precisely, compound 9 demonstrated two hydrogen bonds with THR83 (2.27 Å) and ARG296 (1.86 Å) inside the binding site of AChE (Fig. 2). For Aβ40, compound 9 formed three hydrogen bonds with ASP1 (2.07, 2.80 Å) and LYS16 (1.76 Å). Compound 8 exhibited five hydrogen bonds with ASP1 (2.13 Å), GLU3 (3.06 Å), ASP7 (1.94, 2.14 Å), and GLY9 (2.84 Å) (Fig. 2). Although compound 8 could not form any hydrogen bond within the binding site of AChE, other noncovalent interactions were noticed, involving π–π stacking interactions with PHE297, TYR337, TYR341, and TRP286 (Fig. 2).

**Fig. 3:**
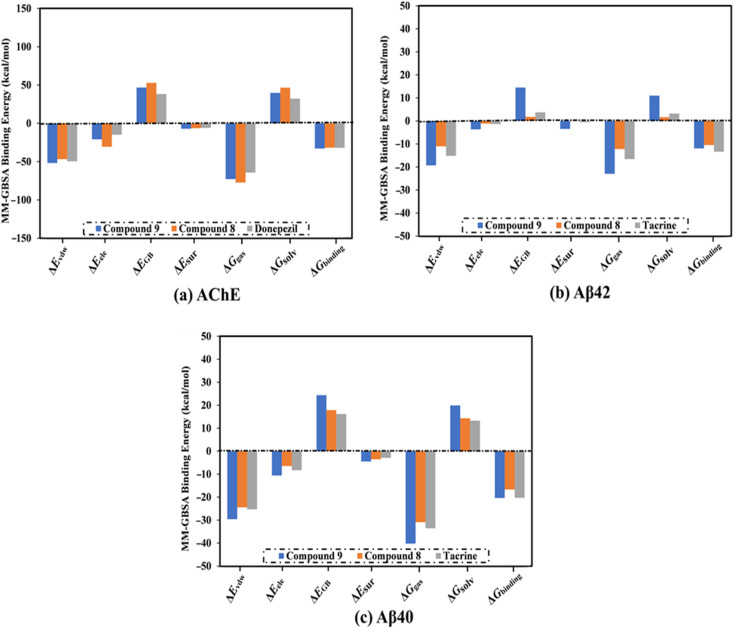
Decomposition of binding affinities for the studied ligands complexed with (a) AChE, (b) Aβ42, and (c) Aβ40 during a period of 100 ns MD.

Compared with compounds 9 and 8, donepezil displayed a similar docking score towards AChE with a value of −11.1 kcal mol^−1^, forming only one hydrogen bond with PHE295 and π–π stacking interactions with TRP86 and TRP286 (Fig. 2). On the other hand, tacrine exhibited one and two hydrogen bonds with GLN15 (1.85 Å) and ASP1 (2.08 Å) and GLY29 (1.92 Å) with the Aβ42 and Aβ40, respectively (Fig. 3).

### 2.4. Molecular dynamics simulations

MD simulations were utilized to puzzle out the stabilization of the ligand–target complex, structural specifics, conformational elasticities, and the trustworthiness of ligand–target binding energy.^31,32^ Consequently, the inspected triazole esters 9 and 8 in complex with AChE and Aβ40/42 were submitted to MD simulations over 100 ns, followed by binding affinity evaluations. The estimated MM-GBSA binding affinities over 100 ns MD simulations are depicted in Fig. 3. As illustrated in Fig. 3, compounds 9 and 8 complexed with AChE exposed competitive binding affinities with an average Δ*G*_binding_ values of −32.9 and −31.8 kcal mol^−1^, respectively, compared to donepezil in complex with AChE (calc. −35.2 kcal mol^−1^). However, compounds 9 and 8 complexed with Aβ42 manifested appropriate binding energies with values of −12.0 and −10.5 kcal mol^−1^, respectively, compared to tacrine (calc. −13.4 kcal mol^−1^) (Fig. 2). A comparison of Δ*G*_binding_ values of compounds 9 and 8 complexed with Aβ42 and those with Aβ40 revealed the higher potency of compounds 9 and 8 with Aβ40 over Aβ42 with Δ*G*_binding_ values of −20.4 and −16.6 kcal mol^−1^, respectively (Fig. 3). The calculated MM-GBSA binding energies were in line with the IC_50_ values.

To determine the most crucial interactions between ligand and target, binding affinities of the studied ligands in complex with AChE and Aβ40/42 were decomposed and illustrated in Fig. 3. As shown in Fig. 3, the binding energies of compounds 9 and 8 and donepezil complexed with AChE were dominated by *E*_vdw_ interactions with values of −51.8, −46.7, and −49.5 kcal mol^−1^, respectively. *E*_ele_ interactions were appropriate with values of −20.9, −30.6, and −14.8 kcal mol^−1^ for compounds 9 and 8 and donepezil complexed with AChE, respectively (Fig. 3).

For compounds 9 and 8 and tacrine complexed with Aβ42 and Aβ40, *E*_vdw_ interactions were a significant contributor with values of −19.3, −11.0, and −15.2 kcal mol^−1^, and −29.6, −24.5, and −25.3 kcal mol^−1^, respectively (Fig. 3). *E*_ele_ interactions were favorable with values of −3.7, −1.2, and −1.4 and −10.6, −6.5, and −8.3 kcal mol^−1^ for compounds 9 and 8 and tacrine complexed with Aβ42 and Aβ40, respectively (Fig. 3). These binding energies computations provided quantitative evidence of compounds 9 and 8 as anti-Alzheimer (AD) agents.

### 2.5. Post-MD analyses

To confirm the steadiness of compounds 9 and 8 in complex with AChE and Aβ40/42, the complexes were investigated structurally and energetically during a period of 100 ns MD, and the results were compared to those of controls (*i.e.*, donepezil and tacrine).

#### 2.5.1. Binding affinity analysis

**Fig. 4:**
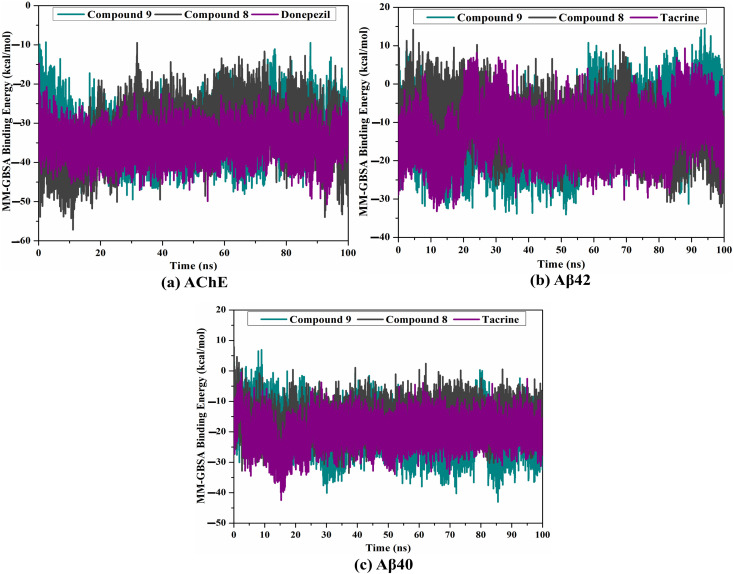
Estimated binding affinity per trajectory for compound 9 (in cyan), compound 8 (in grey), and controls (in purple) with (a) AChE, (b) Aβ42, and (c) Aβ40 during a period of 100 ns MD.

Gauging the correlation between binding affinity per trajectory and time was used to investigate the comprehensive structural steadiness of compounds 9 and 8 complexed with AChE and Aβ40/42 over the 100 ns MD simulations. Overall stability for compounds 9 and 8 was observed with AChE, Aβ42, and Aβ40 with average Δ*G*_binding_ values of −32.9 and −31.8 kcal mol^−1^, −12.0 and −10.5 kcal mol^−1^, and −20.4 and −16.6 kcal mol^−1^, respectively (Fig. 4). Comparing Δ*G*_binding_ values of 9 and 8 with donepezil and tacrine, it can be seen that these compounds are competitive inhibitors compared to controls (Fig. 4). According to binding affinity analysis, all inspected systems preserved stabilization over the 100 ns MD.

#### 2.5.2. Root-mean-square deviation

**Fig. 5:**
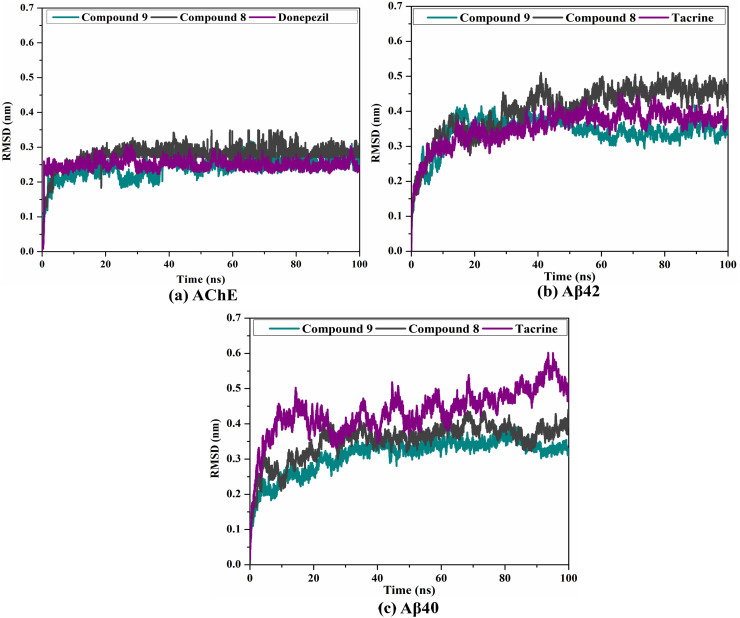
RMSD of the backbone atoms from the initial structure for compound 9 (in cyan), compound 8 (in grey), and controls (in purple) with (a) AChE, (b) Aβ42, and (c) Aβ40 during a period of 100 ns MD.

The RMSD of the backbone atoms of the whole system was inspected to check the conformational stability of 9, 8, and controls complexed with AChE and Aβ40/42 (Fig. 5). As depicted in Fig. 5, the estimated RMSD values for the investigated complexes continued below 0.3 nm during a period of 100 ns MD. The inspected complexes achieved the stabilization state in the first 15 ns MD and preserved their steady till the termination of the simulation. The RMSD data proved that compounds 9 and 8 are tightly bound and do not impact the global topology of AChE and Aβ40/42.

#### 2.5.3. Root-mean-square fluctuation

**Fig. 6:**
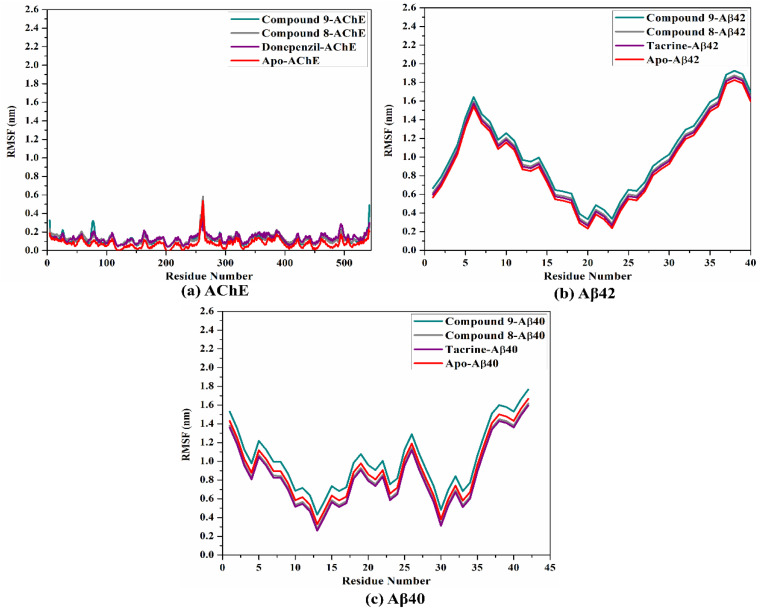
RMSF of the alpha carbon atoms of apo and ligand-soaked AChE and Aβ40/42 during the 100 ns MD course. Color scheme: apo AChE and Aβ40/42 (in red), compound 9 (in cyan), compound 8 (in grey), and controls (in purple) with (a) AChE, (b) Aβ42, and (c) Aβ40.

To inspect the conformational change and steadiness of the backbone of the apo AChE/Aβ, compounds 9, 8, and controls complexed with AChE and Aβ40/42, the root-mean-square fluctuation (RMSF) of alpha carbon was measured and depicted in Fig. 6. As illustrated in Fig. 6, the amino acids were found stable in compounds 9, 8, and controls complexed with AChE and Aβ40/42 during the 100 ns MD course.

## 3. Experimental

### 3.1. General experimental procedures

The progress of reactions and the purity of final products were monitored by thin layer chromatography (TLC), carried out using Merck precoated silica gel F254 plates (E-Merck, Germany) and using vanillin–sulfuric acid spray reagent. Column chromatography was carried out using silica gel G 60-230 (Merck, Germany). The solvents used included *n*-hexane, methylene chloride (CH_2_Cl_2_), and ethyl acetate (EtOAc) used were of reagent grade (El-Nasr Co., Abu Zaabal – Kalyoubia, Cairo, Egypt). ^1^H and APT spectra were measured in CDCl_3_ and DMSO-*d*_6_ using Bruker Avance III HD-400 spectrometer at 400 MHz for ^1^H and 100 MHz for APT in NMR unit, Faculty of Pharmacy, Mansoura, Egypt. Chemical shifts (*δ*) are expressed in ppm with reference to the residual solvent signal. Coupling constants (*J* values) are given in Hz. Melting points were determined on Stuart® melting point apparatus model SMP10 and are uncorrected. High-resolution mass (HR-ESI-MS) was measured using a Bruker microTOF mass spectrometer (Shimadzu, Tokyo, Japan). IR spectra were obtained using a Thermo Scientific Nicolet™ iS™ 10 FT-IR spectrometer instrument.

### 3.2. Chemicals

Propargyl bromide, propargyl alcohol, vanillyl alcohol, ferulic acid, clove oil, sodium azide, *N*,*N*′-dicyclohexylcarbodiimide solution (DCC) and 4-(dimethylamino)pyridine (DMAP) and were purchased from Sigma-Aldrich St. Louis, USA. Vanillin (100% purity) was purchased from Eternal Pearl (The Zhonghua Chemical Factory, Zhejiang, China). Coniferaldehyde and vanillic acid were previously isolated from *Cocos nucifera* L., as reported.^26^ Eugenol was extracted and purified from clove oil using 30% aqueous KOH, according to the literature.^27^

#### 3.2.1. General procedure for preparation of vanillyl azide

Vanillyl alcohol (32 mmol) was dissolved in dry CH_2_Cl_2_ containing catalytic drops of DMF, then thionyl chloride (SOCl_2_) (96 mmol) was added dropwise. The reaction was refluxed and monitored by TLC till the complete reaction.^33,34^ The solvent was evaporated under reduced pressure to give a brown amorphous residue. The oily residue was used as such without any further purification.

Vanillyl chloride (24.15 mmol) was dissolved in 5 ml DMSO, then sodium azide (48.3 mmol) was added. The reaction was stirred at room temperature and monitored by TLC. After completion, the reaction was quenched with water. The crude was extracted with ethyl acetate, dried over anhydrous sodium sulphate and concentrated under a vacuum.^35^ The azide was purified by silica gel column chromatography (2 cm × 28 cm, 35 g) using a gradient elution of ethyl acetate in hexane. The effluents, 50 ml each were collected, concentrated, and screened by TLC. Fractions with the same chromatographic pattern were pooled together. Fractions (7–15), eluted with 5–7% ethyl acetate in hexane, afforded vanillyl azide as a pure compound.

#### 3.2.2. Preparation of alkynes

Five natural vanilloid precursors were used for the preparation of the seven readily clickable propargyl ethers and propargyl esters. Vanillin, eugenol and coniferaldehyde were converted for their respective propargyl ethers using propargyl bromide. Vanillic and ferulic acid were converted to respective propargyl ethers and esters using propargyl bromide and propargyl alcohol, respectively.

##### 3.2.2.1. General procedure for preparation of alkynes by propargylic etherification

The start vanilloid was stirred with K_2_CO_3_ (3.5 equivalent) in dry acetone at room temperature for 2 hours. Propargyl bromide (1.3 equivalents) was added to the mixture was stirred under reflux at 80 °C for 10 h.^36^ After the completion of the reaction confirmed by TLC, the reaction was stopped by the addition of water. The solid salts were separated by filtration, and the product was extracted with ethyl acetate (3 × 50 ml). The ethyl acetate was concentrated under reduced pressure to give corresponding alkynes.

For vanillic or ferulic acid, before preparation of the corresponding propargyl ethers, the carboxylic group was masked by esterification.^37^ Vanillic (500 mg, 1.7 mmol) or ferulic acid (500 mg, 2.05 mmol) were dissolved in 25 ml absolute EtOH containing 1 ml conc. H_2_SO_4_ and refluxed.^37^

##### 3.2.2.2. General procedure for preparation of alkynes by propargylic esterification

Prior to the preparation of the corresponding propargyl ester, the hydroxyl group was masked by acetylation. Vanillic (300 mg, 1.7 mmol) or ferulic acid (400 mg, 2.05 mmol), and pyridine (2 mmol), were dissolved in acetic anhydride (2 ml, 18.1 mol).^38^ The reaction was stirred for 12 h and monitored by TLC for completion.

Acetyl vanillic acid (360 mg, 1.7 mmol) or acetyl ferulic acid (415 mg, 1.75 mmol) and propargyl alcohol (1.7 mmol) were dissolved in dry CH_2_Cl_2_. To the stirred mixture, DCC (3.4 mmol) and DMAP (0.17 mmol) were added dropwise. The reaction mixture was monitored by TLC for completion. After 24 h stirring at room temperature, the reaction mixture was filtered over silica and the solution was washed with CH_2_Cl_2_ and concentrated under reduced pressure.

#### 3.2.3. General procedure for preparation of triazole derivatives by click reaction

Equimolar amounts of the alkynes (1–7a) and vanillyl azide were dissolved in 2 ml CH_2_Cl_2_ : H_2_O (1 : 1). To this mixture, CuSO_4_·5H_2_O (0.15 eq.) and ascorbic acid (0.45 eq.) were added and stirred at room temperature. The reaction was monitored by TLC till completion. The reaction was quenched with distilled water and extracted with ethyl acetate, dried over anhydrous Na_2_SO_4_ and concentrated under a vacuum.^39^ The product was purified by silica gel column chromatography using gradient elution of ethyl acetate in hexane as to give 1,2,3-triazole derivatives.

#### 3.2.4. Hydrolysis of triazole esters into the corresponding alcohol

The triazole esters 4 and 5 were dissolved in 1 M aqueous NaOH and stirred at room temperature. After reaction completion, 1 N HCl was added dropwise till neutral pH. The product was extracted using ethyl acetate.

### 3.3. Biological evaluation

#### 3.3.1. Acetylcholine esterase inhibition assay

The inhibition of acetylcholine esterase (AChE) enzyme activity was measured using the colorimetric Acetylcholinesterase Inhibitor Screening Kit (155 S. Milpitas Blvd., Milpitas, CA 95035 USA) according to manufacturer's instructions.

#### 3.3.2. Amyloid-β aggregation inhibition assay

The β-amyloid aggregation inhibition was measured using the fluorometric SensoLyte® Thioflavin T Beta-Amyloid (1–42) Aggregation Kit (ANASPEC Inc., Fremont, CA., USA) according to the manufacturer's instructions.

### 3.4. *In silico* computations

#### 3.4.1. Target preparation

The X-ray resolved three-dimensional structure of human acetylcholine esterase (PDB ID: 4EY7,^40^ resolution: 2.35 Å), and NMR structures of monomeric amyloid-beta peptide (Aβ42/40) (PDB ID: 1IYT^41^/1BA4 (ref. 42)) were obtained and utilized as templates for all *in silico* computations. For the AChE and Aβ40/42 preparation, all crystallographic water molecules, ligands, ions, and heteroatoms were eliminated. The empirical program PropKa was utilized to determine the protonation state of titratable residues of the investigated targets.^43^

#### 3.4.2. Ligand preparation

The chemical structures of triazole esters 9 and 8 were manually created. Omega2 software was applied to convert two-dimensional formats into three-dimensional structures.^44,45^ The compounds were then minimized using an MMFF94S force field within SZYBKI software.^46,47^ The Gasteiger–Marsili method was used to assign the atomic charges of these compounds.^48^

#### 3.4.3. Molecular docking

All docking predictions were executed using AutoDock4.2.6 software.^49^ A maximum of 25 000 000 energy evaluations and 250 independent runs were employed for docking computations. The remaining docking parameters were kept at the default settings. The AutoGrid program was employed to construct the grid maps. A grid box with dimensions 50 Å × 50 Å × 50 Å (*x*, *y*, *z* directions) was set around the binding pocket of AChE. The grid spacing value of 0.375 Å was utilized. The grid of AChE was positioned at the coordinates *X* = 11.367, *Y* = −56.25, and *Z* = −22.605. For Aβ40/42 targets, the AutoDock-based blind docking strategy was utilized in the current study.

#### 3.4.4. MD simulations

Molecular dynamics (MD) simulations were conducted using AMBER16 software for the studied ligands in complex with AChE and Aβ40/42 at a time scale of 100 ns.^50^ The details of the employed MD simulations are described elsewhere.^51–53^ Briefly, the parameters of the investigated ligands were generated utilizing the general AMBER force field (GAFF2).^54^ AMBER force field 14SB was employed for AChE and Aβ40/42 parametrization.^55^ The geometry optimization was executed at the HF/6-31G* level of theory using Gaussian 09 software.^56^ Thereafter, the restrained electrostatic potential (RESP) approach was utilized to compute the atomic charges of the investigated compounds.^57^ An octahedron box with a distance of 1.2 nm was used to solvate the inspected target–inhibitor complexes using the TIP3P water model. The inspected complexes were neutralized using Na^+^ and Cl^−^ ions.^58^ The solvated complexes were minimized for 5000 cycles. The minimized complexes were smoothly heated up to 300 K during a period of 50 ps. Subsequently, equilibration of the systems was performed for 10 ns, followed by a production stage of 100 ns. All MD simulations were conducted with the pmemd.cuda module implemented within AMBER16 software on the CompChem GPU/CPU cluster (https://hpc.compchem.net). The BIOVIA Discovery Studio Visualizer 2020 was employed to depict the molecular interactions.^59^

#### 3.4.5. Binding energy estimation

The molecular mechanical/generalized born surface area (MM/GBSA) approach was utilized to compute the binding energies of the inspected ligands in complex with AChE and Aβ40/42.^60^ Uncorrelated trajectories were gathered every 10 ps during the production run for the MM/GBSA computations. The binding energy (Δ*G*_binding_) was evaluated using the following equation:Δ*G*_binding_ = *G*_complex_ − (*G*_ligand_ + *G*_target_)

## 4. Conclusion

Based on their reported neuroprotective properties, the authors have selected the vanilloid pharmacophore to design potential anti-AD drugs. Nine new vanilloids hybrids (1–9) were semi-synthetized *via* click reactions of vanillyl azide and several vanilloid monoalkynes. Compounds 9 and 8 showed remarkable ACE and Aβ aggregation inhibition. The results suggested that the triazole ester moiety may be favorable for the activity over the triazole ether moiety. The results showed that compounds 9 and 8 are promising dual AChE/Aβ aggregation inhibitors. They may serve as potential leads for designing novel anti-Alzheimer agents.

## Author contributions

Marwa Elsbaey and Eman Elattar: conceptualization, investigation, methodology, writing – review & editing; Mahmoud Ibrahim: formal analysis, software, writing – review & editing; Yasuhiro Igarashi: supervision.

## Conflicts of interest

There are no conflicts to declare.

## Supplementary Material

RA-013-D2RA07539C-s001
