# Supplementary material for: Click-designed vanilloid-triazole conjugates as dual inhibitors of AChE and Aβ aggregation
Source: RSC Adv. 2023 Jan 19;13(5):2871–83. doi: 10.1039/d2ra07539c (PMC9850456; doi:10.1039/d2ra07539c)
Supplement: RA-013-D2RA07539C-s001 [file RA-013-D2RA07539C-s001.pdf]

Electronic Supplementary Material (ESI) for RSC Advances.
This journal is © The Royal Society of Chemistry 2023

Supporting information

Click designed vanilloid-triazole conjugates as dual inhibitors of AChE and Aβ aggregation

Marwa Elsbaey[1*], Yasuhiro Igarashi[2], Mahmoud A. A. Ibrahim[3,4], Eman Elattar[1]

[1]Department of Pharmacognosy, Faculty of Pharmacy, Mansoura University, 35516, Egypt

[2]Biotechnology Research Center and Department of Biotechnology, Toyama Prefectural University, 5180 Kurokawa, Imizu, Toyama, 939-0398, Japan

[3]Computational Chemistry Laboratory, Chemistry Department, Faculty of Science, Minia University, 61519, Egypt

[4]School of Health Sciences, University of Kwa-Zulu-Natal, Westville, Durban 4000, South Africa

*Correspondence author: Marwa Elsbaey (PhD of Pharmacognosy, 2014)

Postal address: 35516- Mansoura University building- Dakahlia- North Delta- Egypt

Tel.: 00201066364811; Fax: +02(050)2247496.

Email: marwaelsebay1611@mans.edu.eg

Table of content

Content	Page
Figure S1: ^{1}H-NMR spectrum of compound 1 (DMSO-*d6*, 400 MHz).	3
Figure S2: APT spectrum of compound 1 (DMSO-*d6*, 100 MHz).	4
Figure S3: Positive HRESI-Mass of compound 1	5
Figure S4: Negative HRESI-Mass of compound 1	6
Figure S5: IR spectrum of compound 1	7
Figure S6: ^{1}H-NMR spectrum of compound 2 ($CDCl_3$, 400 MHz).	8
Figure S7: APT spectrum of compound 2 ($CDCl_3$, 100 MHz).	9
Figure S8: Positive HRESI-Mass of compound 2	10
Figure S9: IR spectrum of compound 2.	11
Figure S10: ^{1}H-NMR spectrum of compound 3 ($CDCl_3$, 400 MHz).	12
Figure S11: APT spectrum of compound3 ($CDCl_3$, 100 MHz).	13
Figure S12: Positive HRESI-Mass of compound 3	14
Figure S13: Negative HRESI-Mass of compound 3	15
Figure S14: IR spectrum of compound 3.	`16
Figure S15: ^{1}H-NMR spectrum of compound 4 (DMSO-*d6*, 400 MHz).	17
Figure S16: APT spectrum of compound 4 (DMSO-*d6*, 100 MHz).	18
Figure S17: HMBC of compound 4 (DMSO-*d6*, 100 MHz).	19
Figure S18: Negative HRESI-Mass of compound 4	20
Figure S19: IR spectrum of compound 4.	21
Figure S20: ^{1}H-NMR spectrum of compound 5 (DMSO-*d6*, 400 MHz).	22
Figure S21: APT spectrum of compound 5 (DMSO-*d6*, 100 MHz).	23
Figure S22: Positive HRESI-Mass of compound 5.	24
Figure S23: IR spectrum of compound 5.	25
Figure S24: ^{1}H-NMR spectrum of compound 6 (DMSO-*d6*, 400 MHz).	26
Figure S25: APT spectrum of compound 6 (DMSO-*d6*, 100 MHz).	27
Figure S26: HMBC of compound 6 (DMSO-*d6*, 100 MHz).	28
Figure S27: Negative HRESI-Mass of compound 6.	29
Figure S28: IR spectrum of compound 6.	30
Figure S29: ^{1}H-NMR spectrum of compound 7 (DMSO-*d6*, 400 MHz).	31
Figure S30: APT spectrum of compound 7 (DMSO-*d6*, 100 MHz).	32
Figure S31: Negative HRESI-Mass of compound 7.	33
Figure S32: IR spectrum of compound 7.	34
Figure S33: ^{1}H-NMR spectrum of compound 8 ($CDCl_3$, 100 MHz).	35
Figure S34: APT spectrum of compound 8 ($CDCl_3$, 100 MHz).	36
Figure S35: Positive HRESI-Mass of compound 8.	37
Figure S36: IR spectrum of compound 8.	38
Figure S37: ^{1}H-NMR spectrum of compound 9 (DMSO-*d6*, 400 MHz).	39
Figure S38: APT spectrum of compound 9 (DMSO-*d6*, 100 MHz).	40
Figure S39: HMBC of compound 9 (DMSO-*d6*, 100 MHz).	41
Figure S40: Negative HRESI-Mass of compound 9.	42
Figure S41: IR spectrum of compound 9.	43
Figure S42: UV spectrum of compounds 1-4.	44
Figure S43: UV spectrum of compounds 5-8.	45
Figure S44: UV spectrum of compound 9.	46
Biological evaluation	47

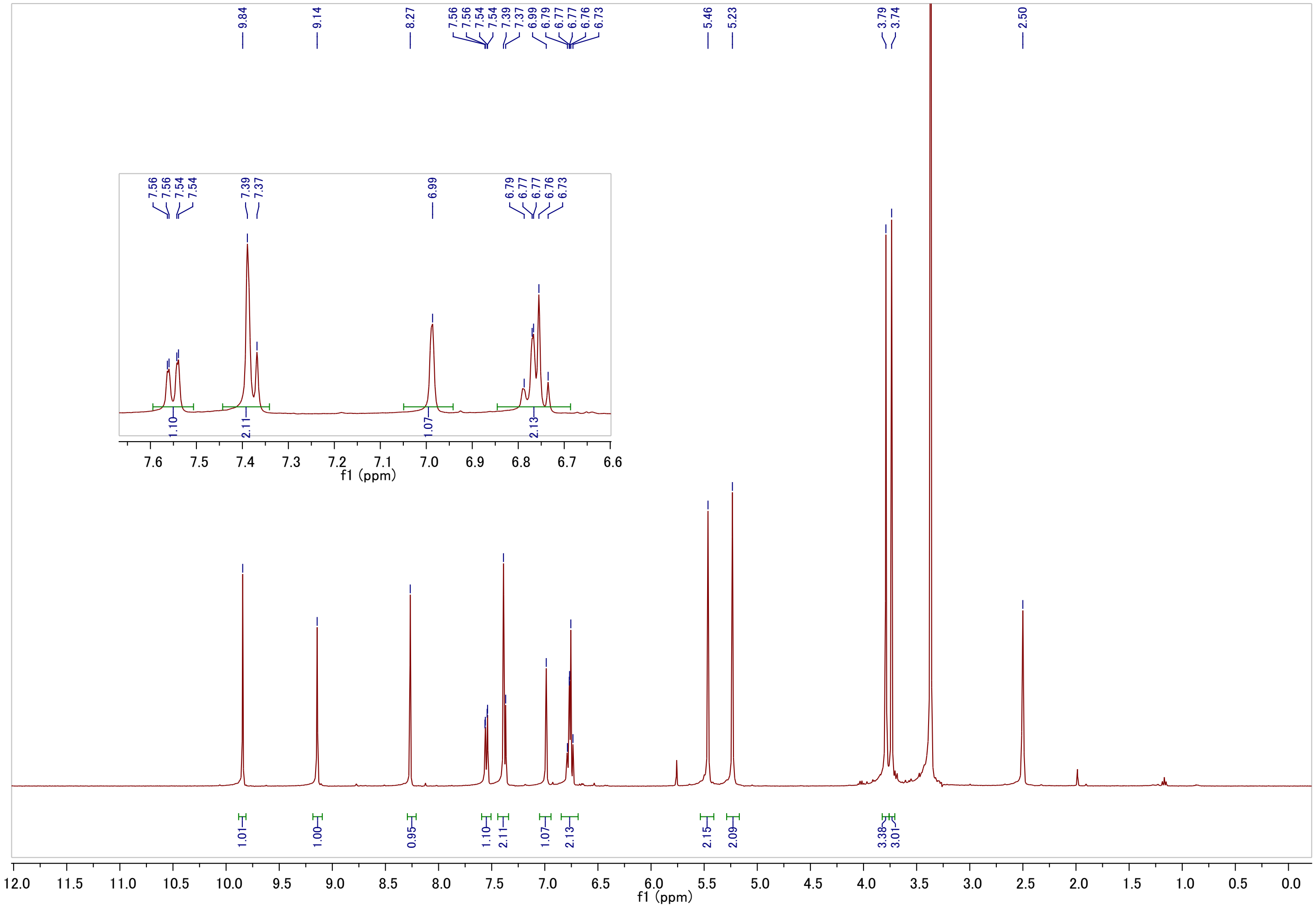

Figure S1: ^{1}H-NMR spectrum of compound 1 (DMSO-*d6*, 400 MHz).

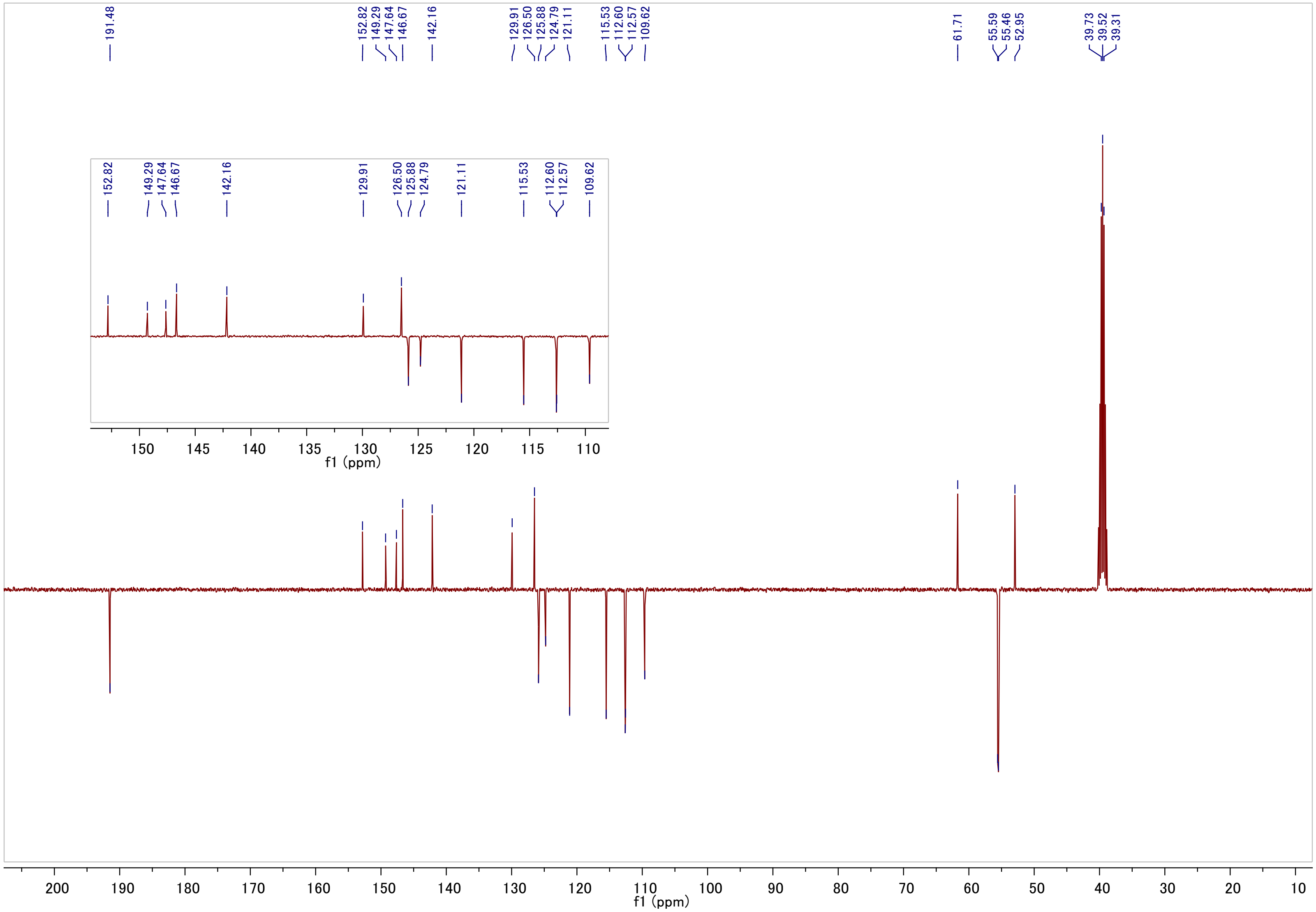

Figure S2: APT spectrum of compound 1 (DMSO-*d6*, 100 MHz).

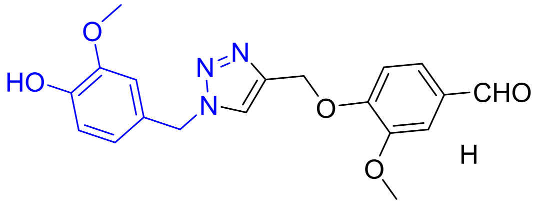

Chemical Formula: $C_{19}H_{20}N_3O_5$
Exact Mass: 370.1403

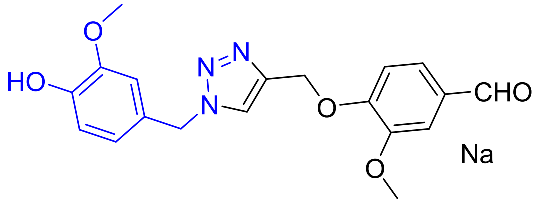

M+ Na
Chemical Formula: $C_{19}H_{20}N_3NaO_5$
Exact Mass: 392.1222

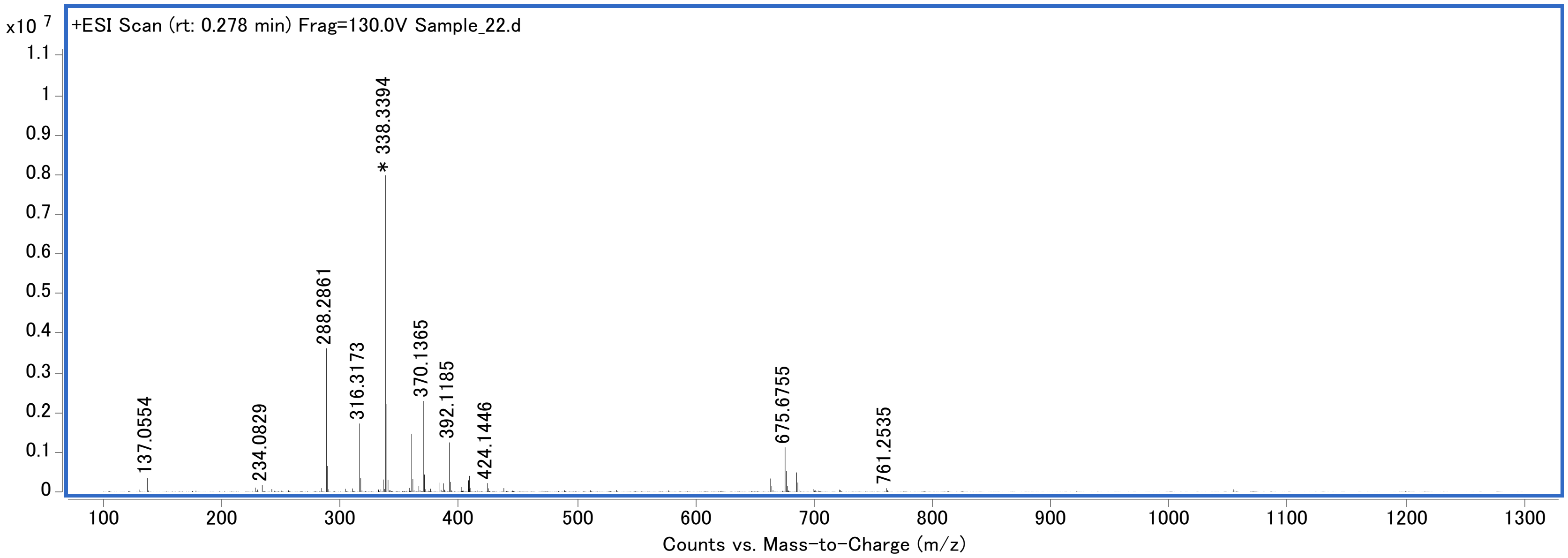

Figure S3: Positive HRESI-Mass of compound 1.

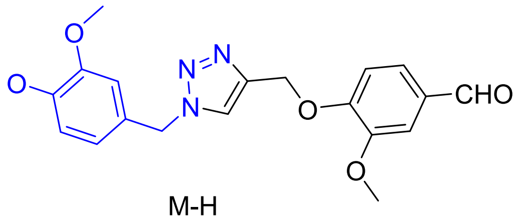

M-H
Chemical Formula: $C_{19}H_{18}N_3O_5$
Exact Mass: 368.1246

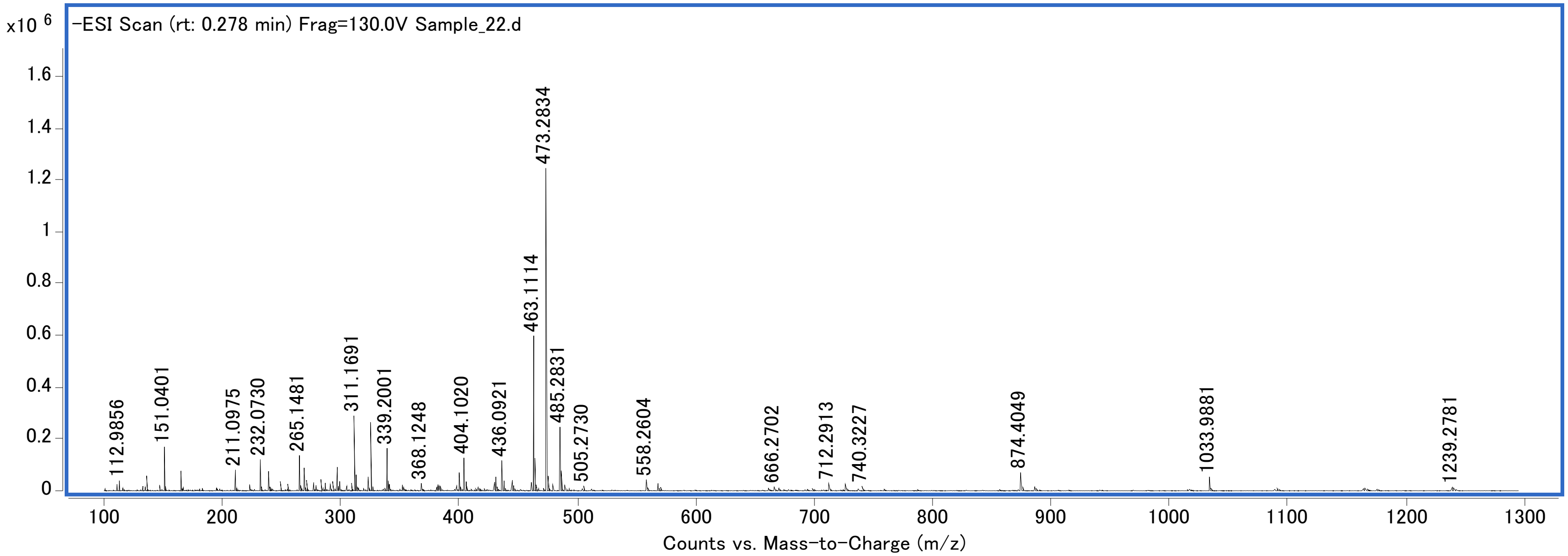

Figure S4: Negative HRESI-Mass of compound 1

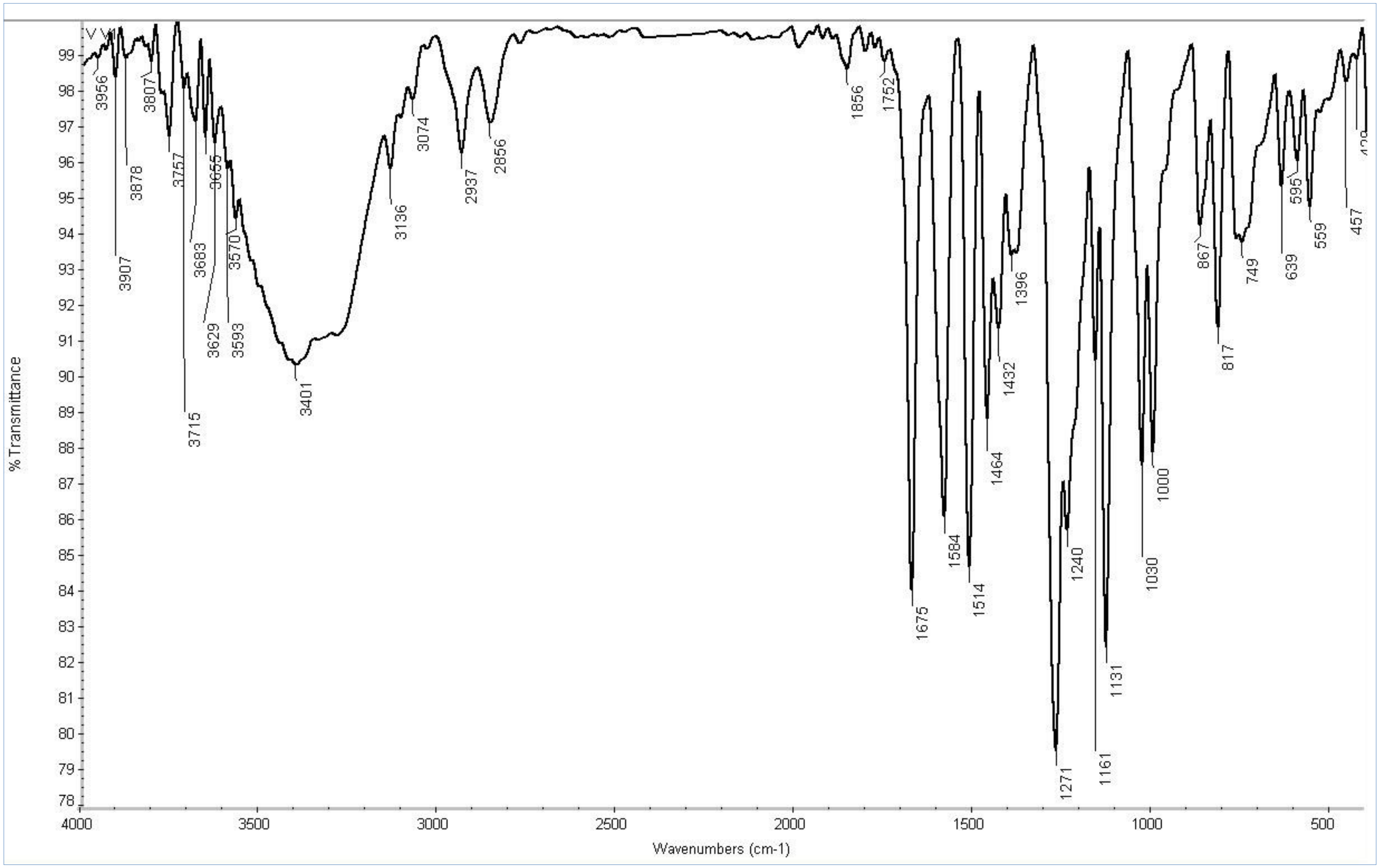

Figure S5: IR spectrum of compound 1

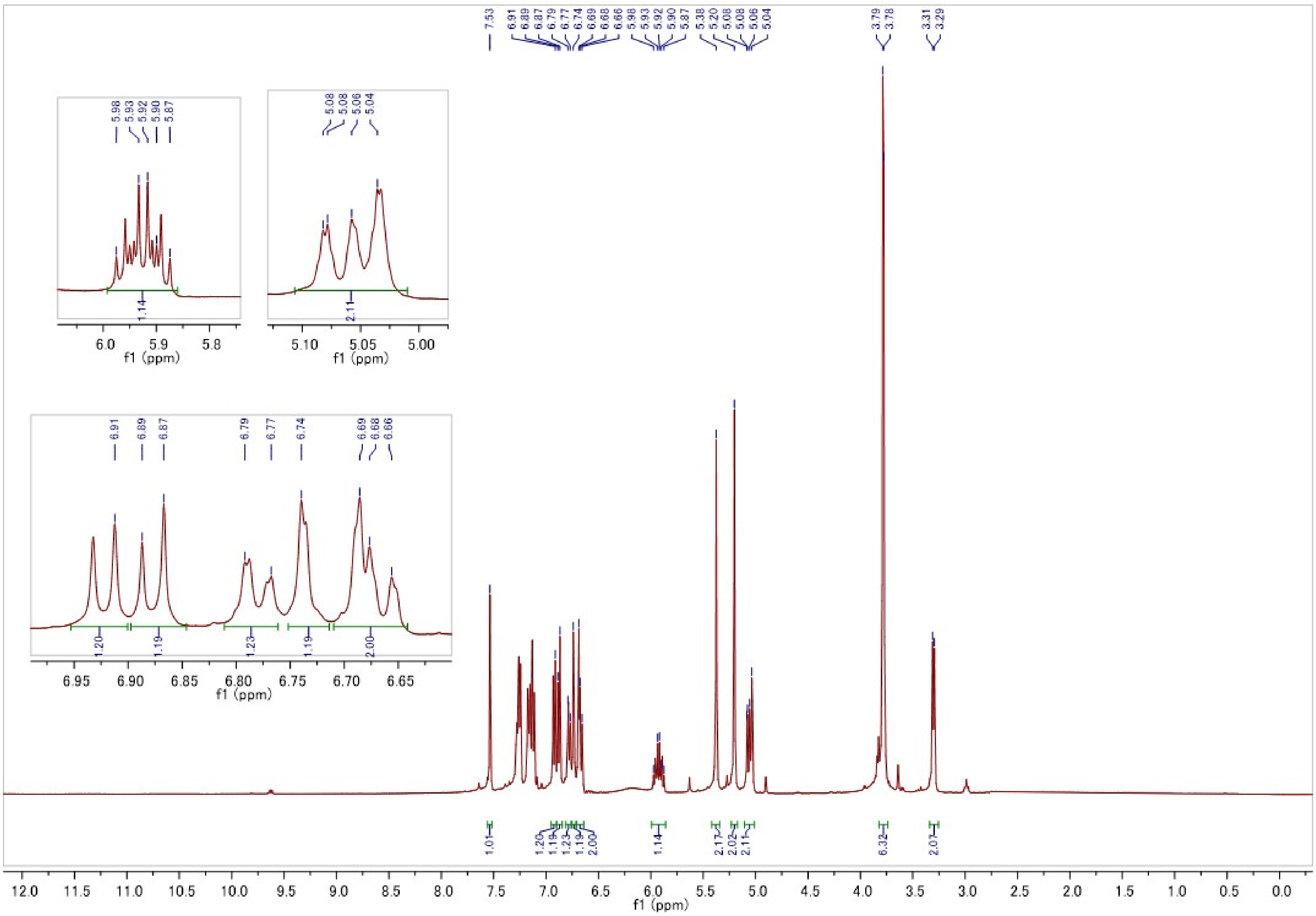

Figure S6: ^{1}H-NMR spectrum of compound 2 ($CDCl_3$, 400 MHz).

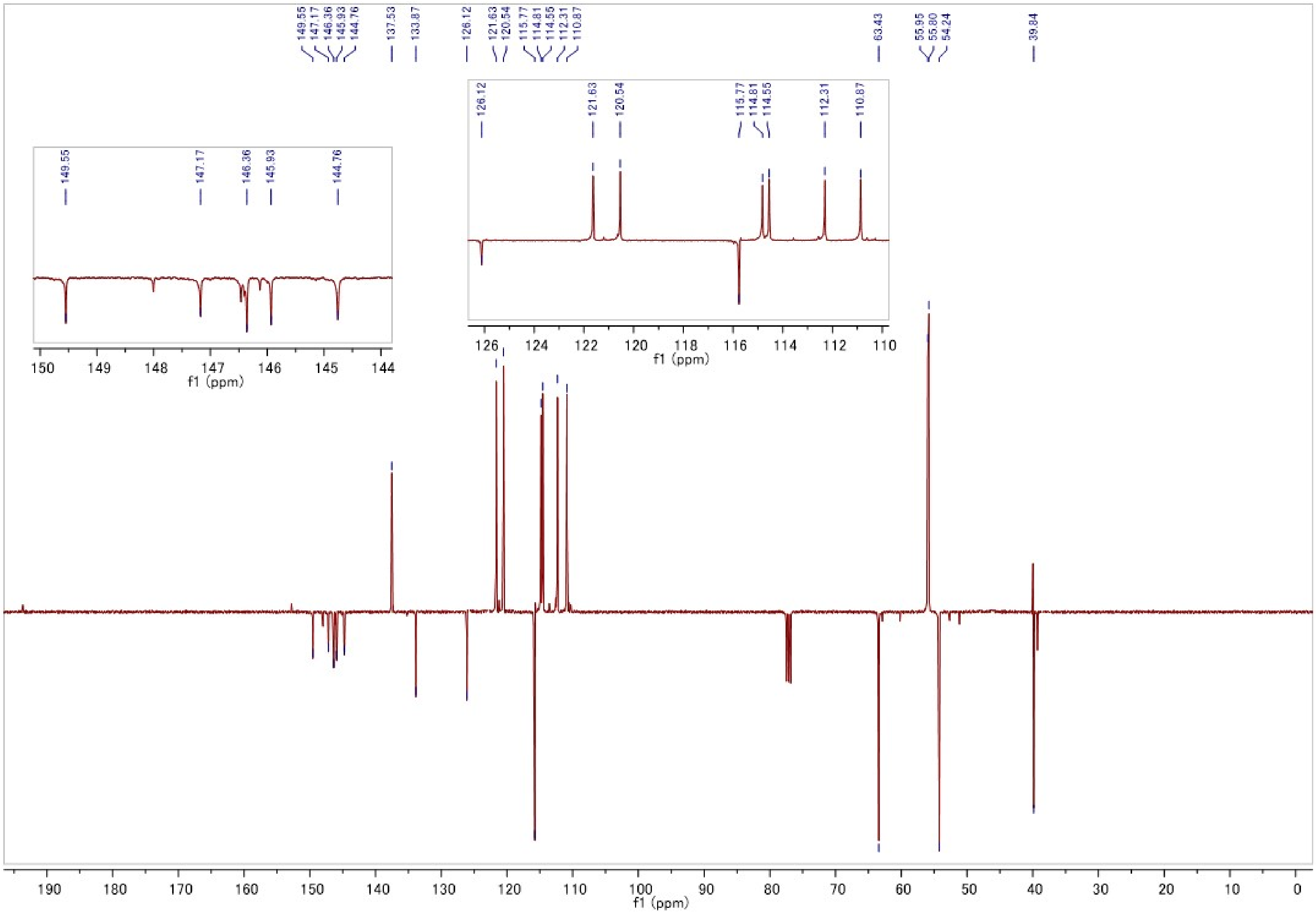

Figure S7: APT spectrum of compound 2 ($CDCl_3$, 100 MHz).

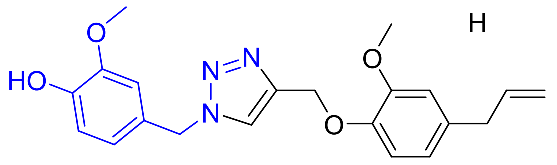

M+H
Chemical Formula: C21H24N3O4
Exact Mass: 382.1767

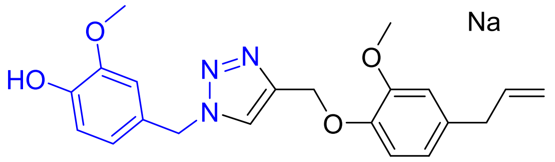

M+Na
Chemical Formula: C21H23N3NaO4
Exact Mass: 404.1586

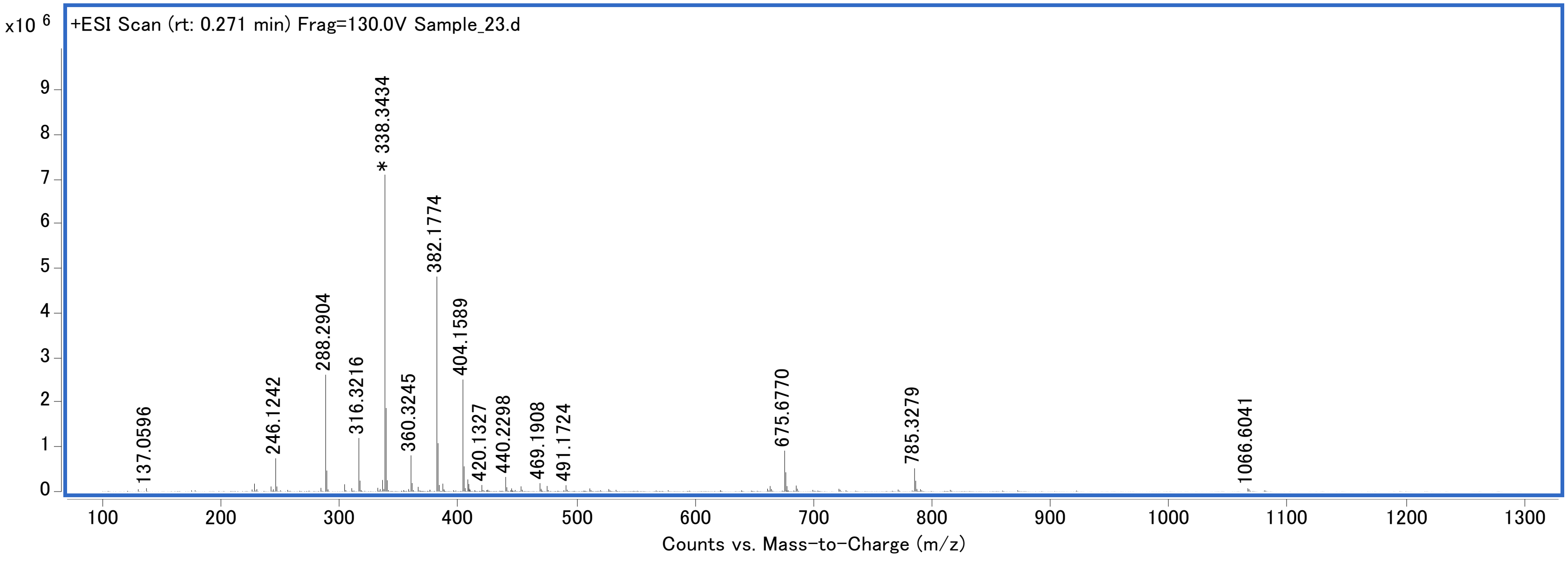

Figure S8: Positive HRESI-Mass of compound 2.

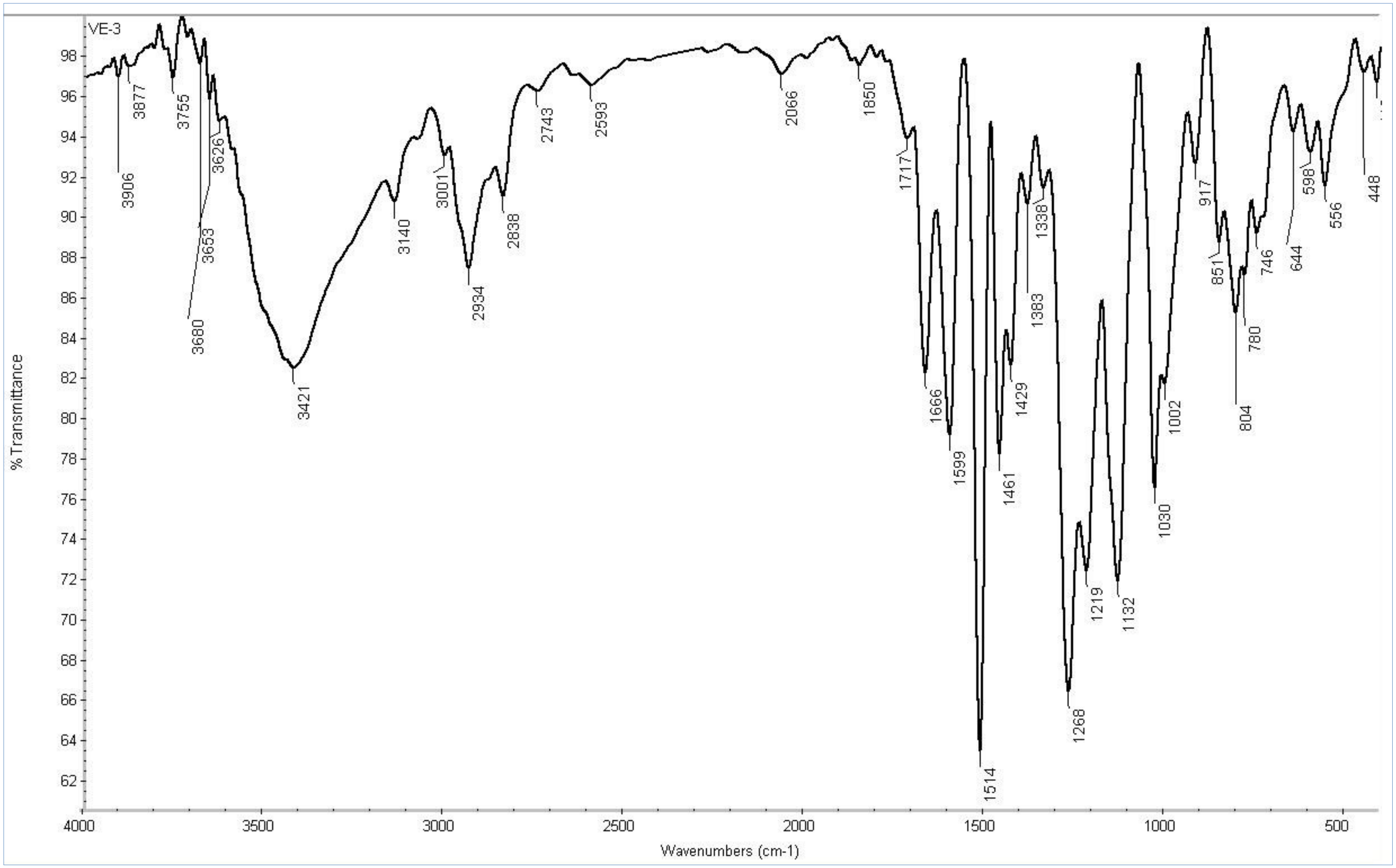

Figure S9: IR spectrum of compound 2.

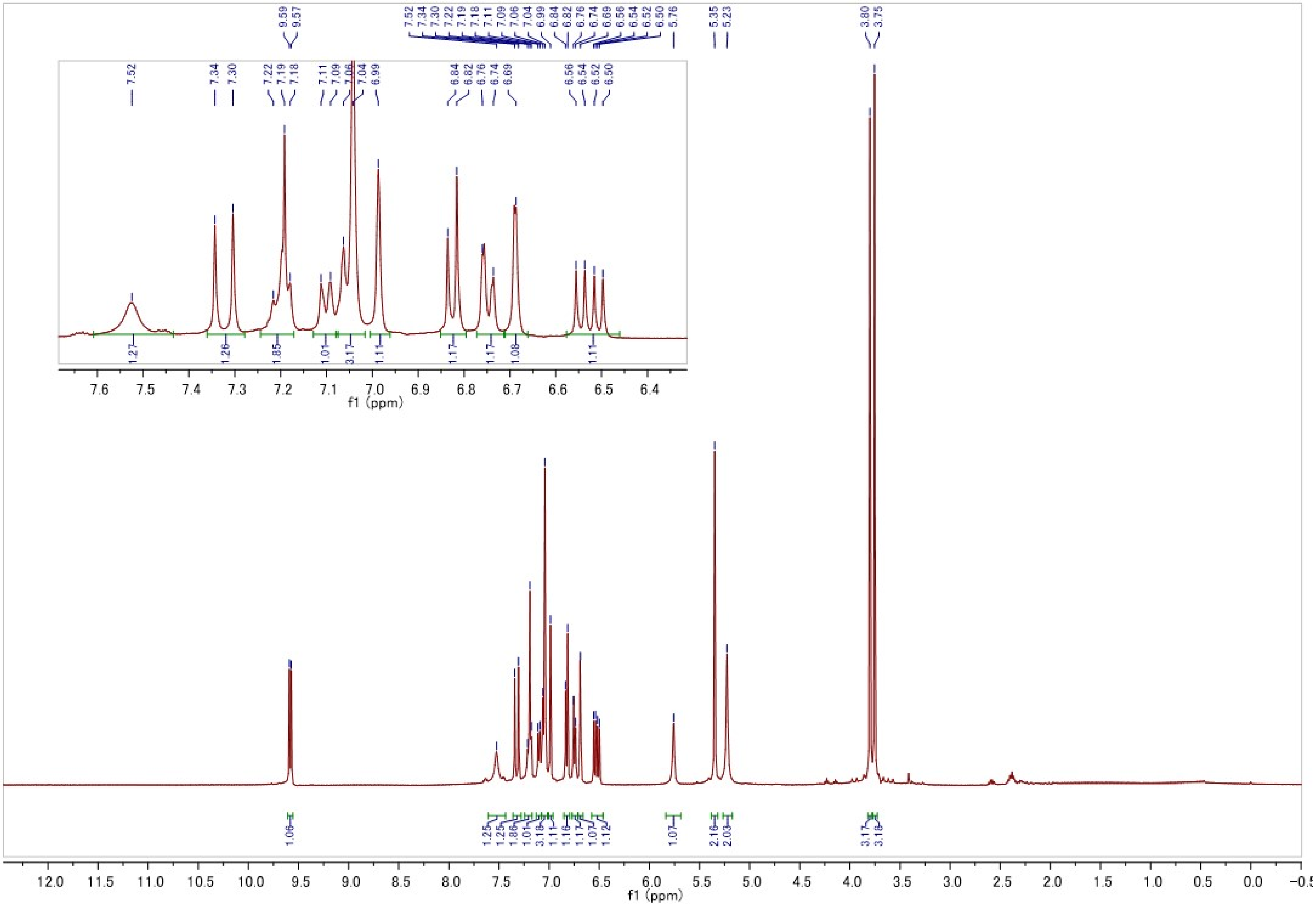

Figure S10: ^{1}H-NMR spectrum of compound 3 ($CDCl_3$, 400 MHz).

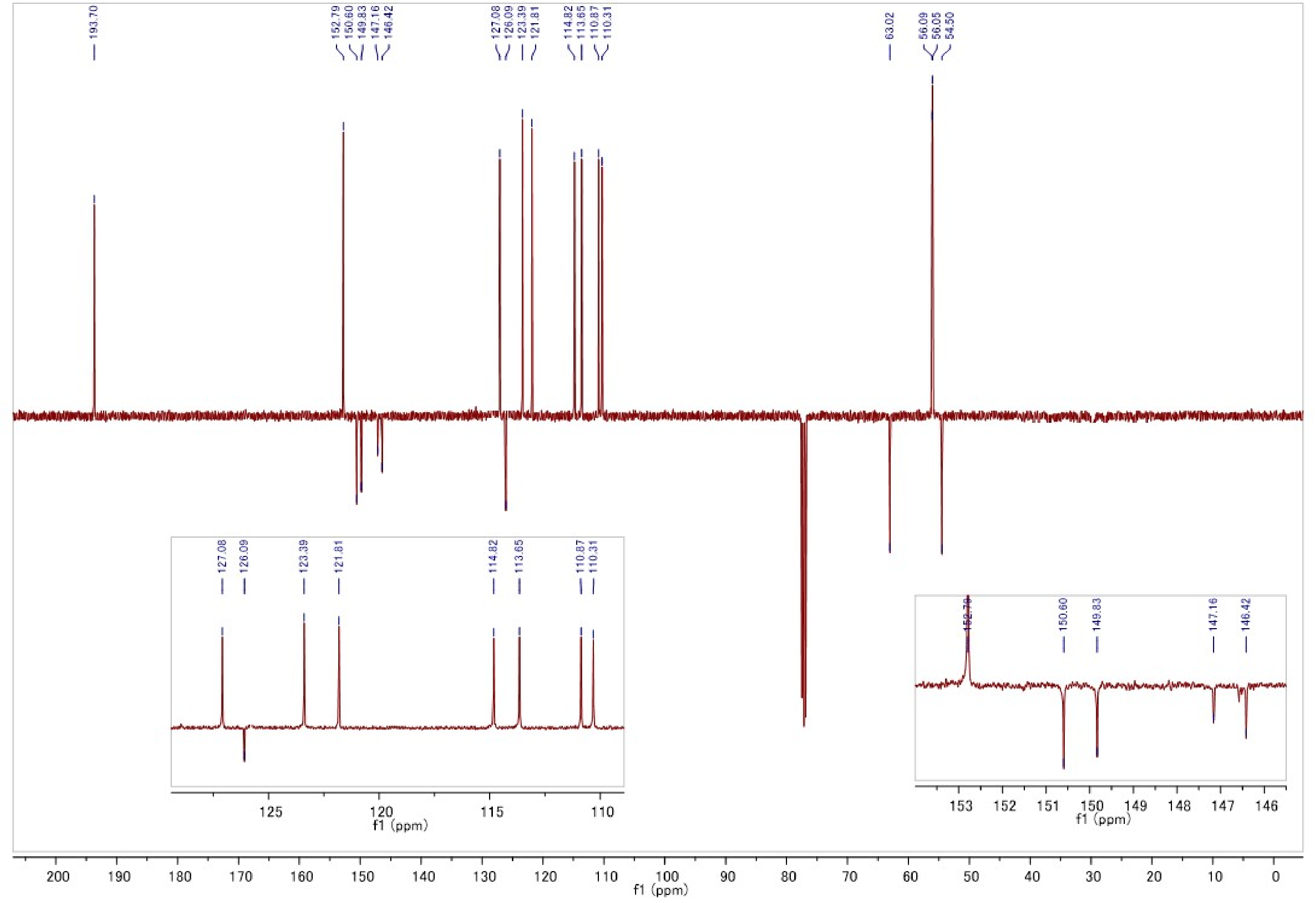

Figure S11: APT spectrum of compound3 ($CDCl_3$, 100 MHz).

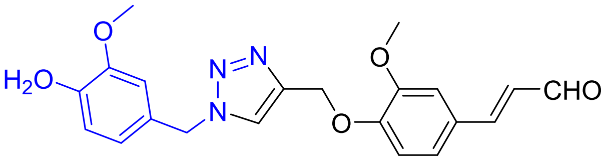

M+H
Chemical Formula: $C_{21}H_{22}N_3O_5$
Exact Mass: 396.1559

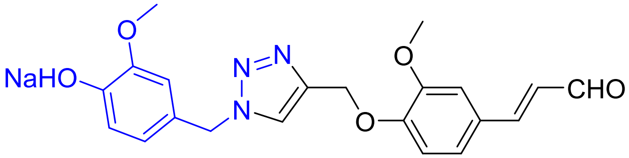

M+Na
Chemical Formula: $C_{21}H_{21}N_3NaO_5$
Exact Mass: 418.1379

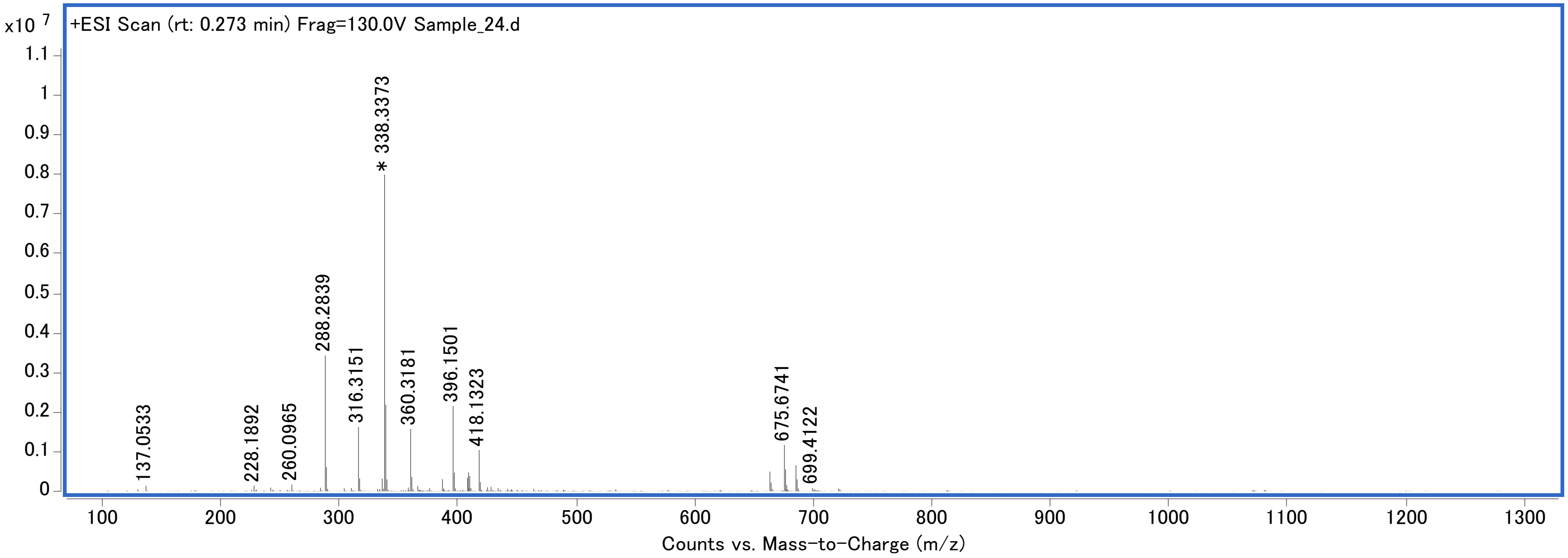

Figure S12: Positive HRESI-Mass of compound 3.

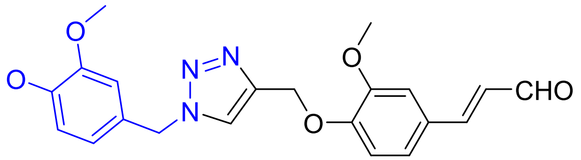

M-H
Chemical Formula: $C_{21}H_{20}N_3O_5$
Exact Mass: 394.1403

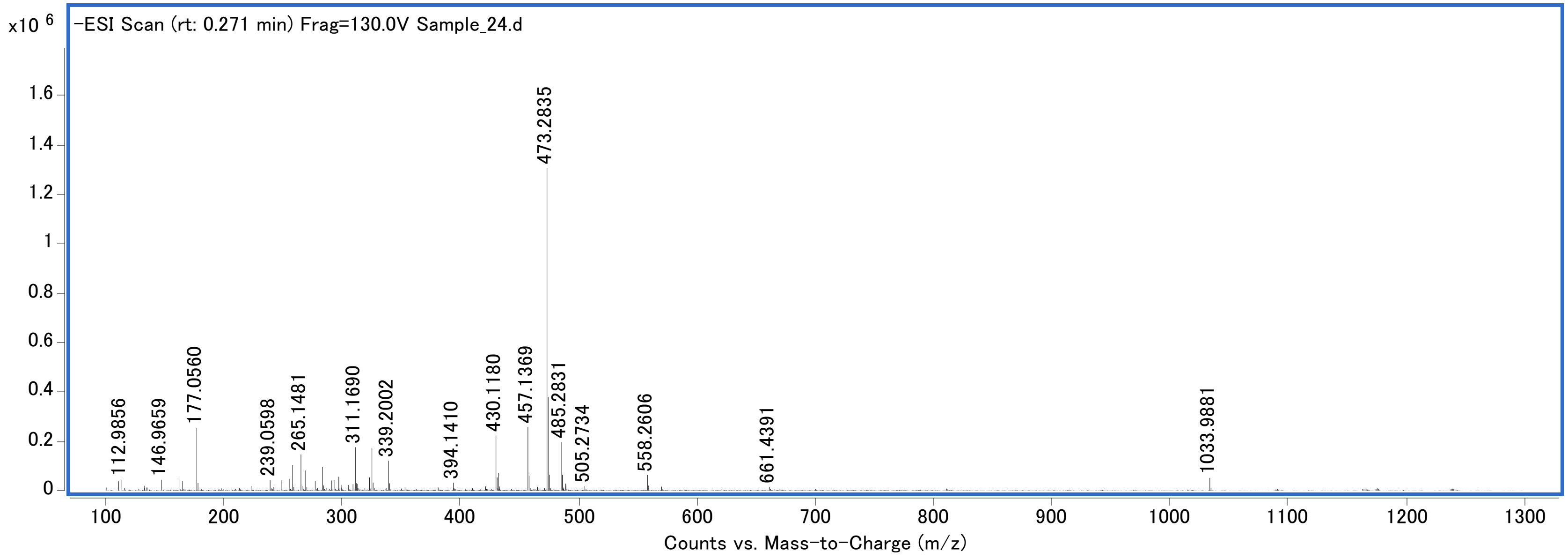

S13: Negative HRESI-Mass of compound 3.

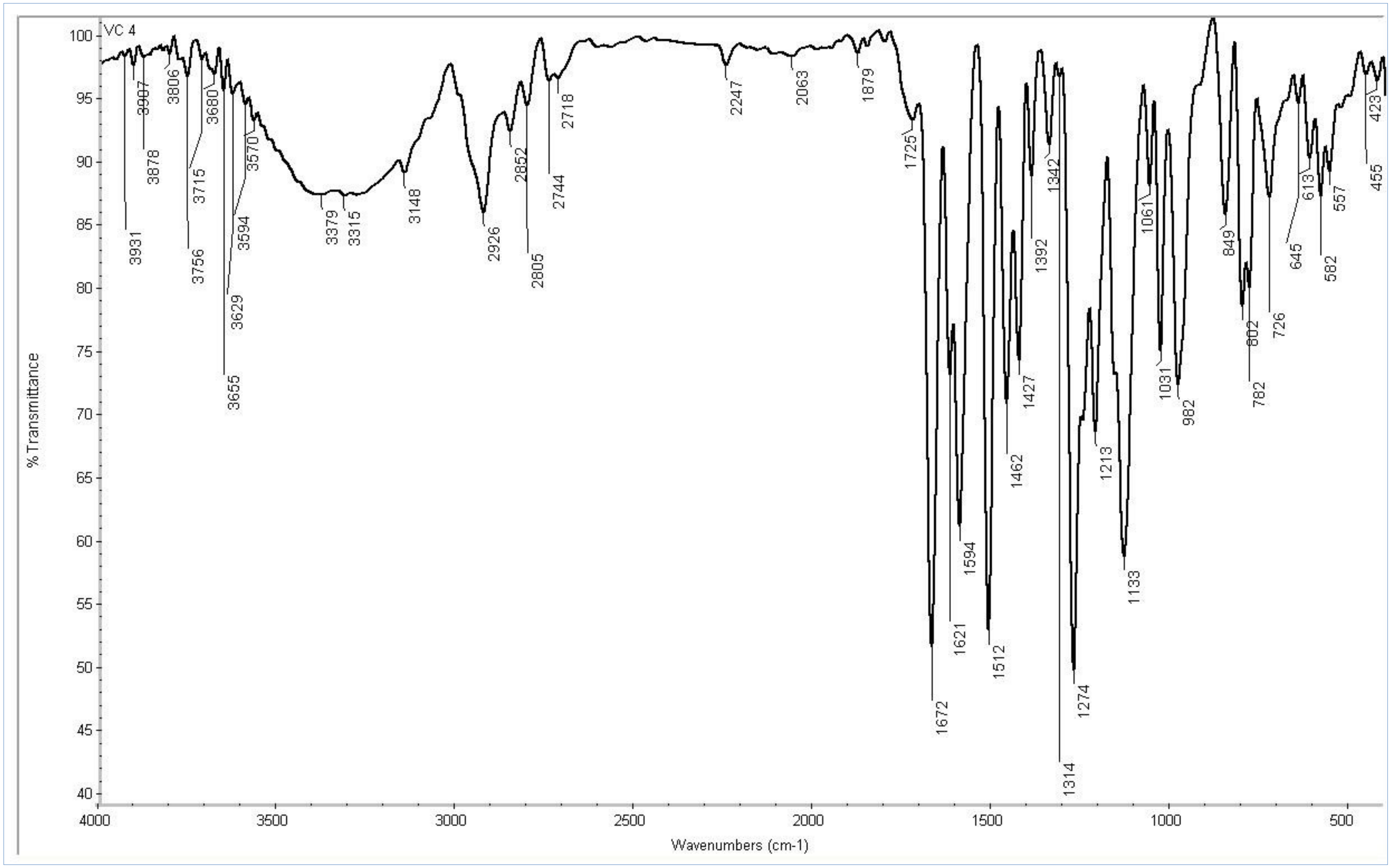

Figure S14: IR spectrum of compound 3.

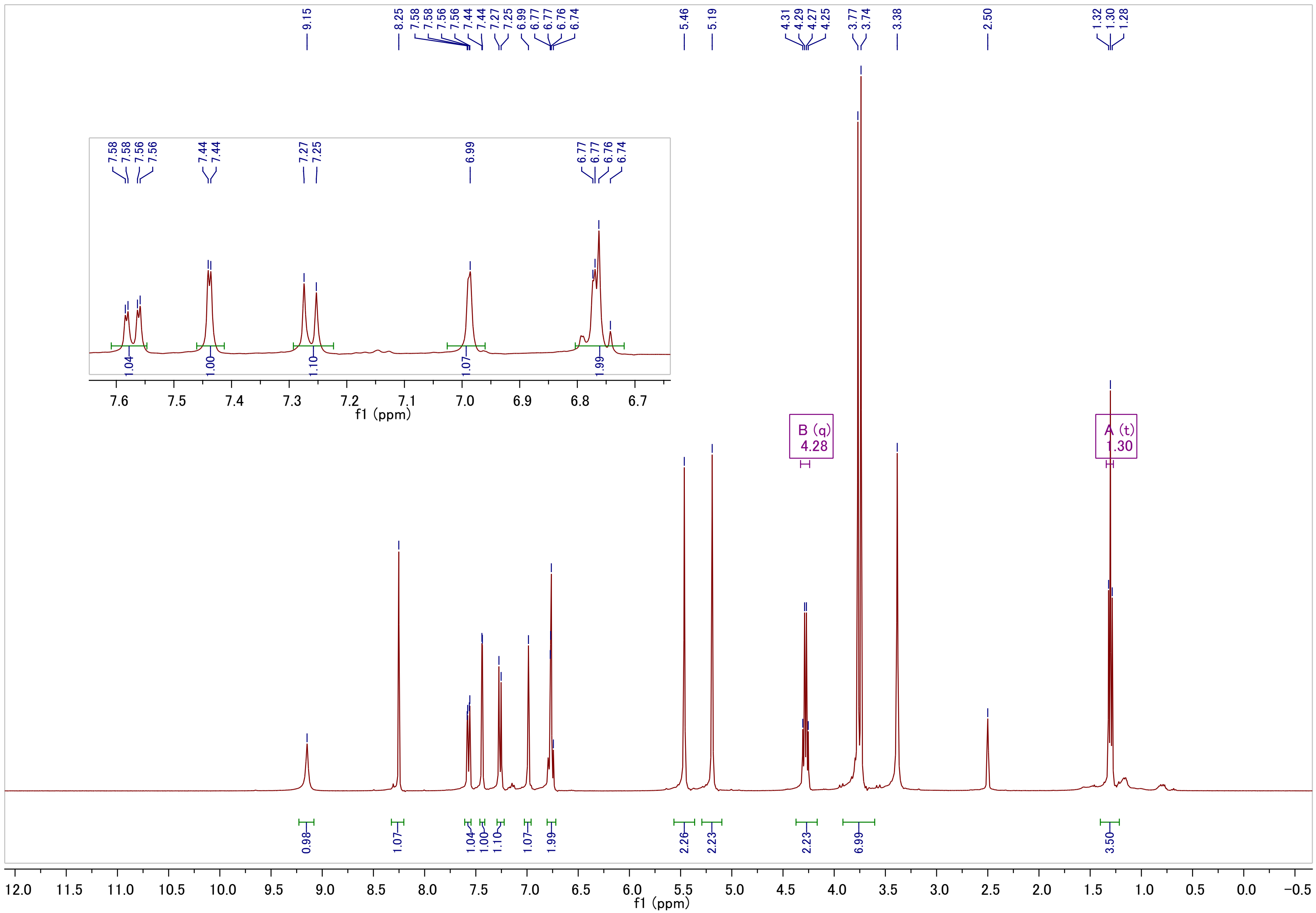

Figure S15: ^{1}H-NMR spectrum of compound 4 (DMSO-*d6*, 400 MHz).

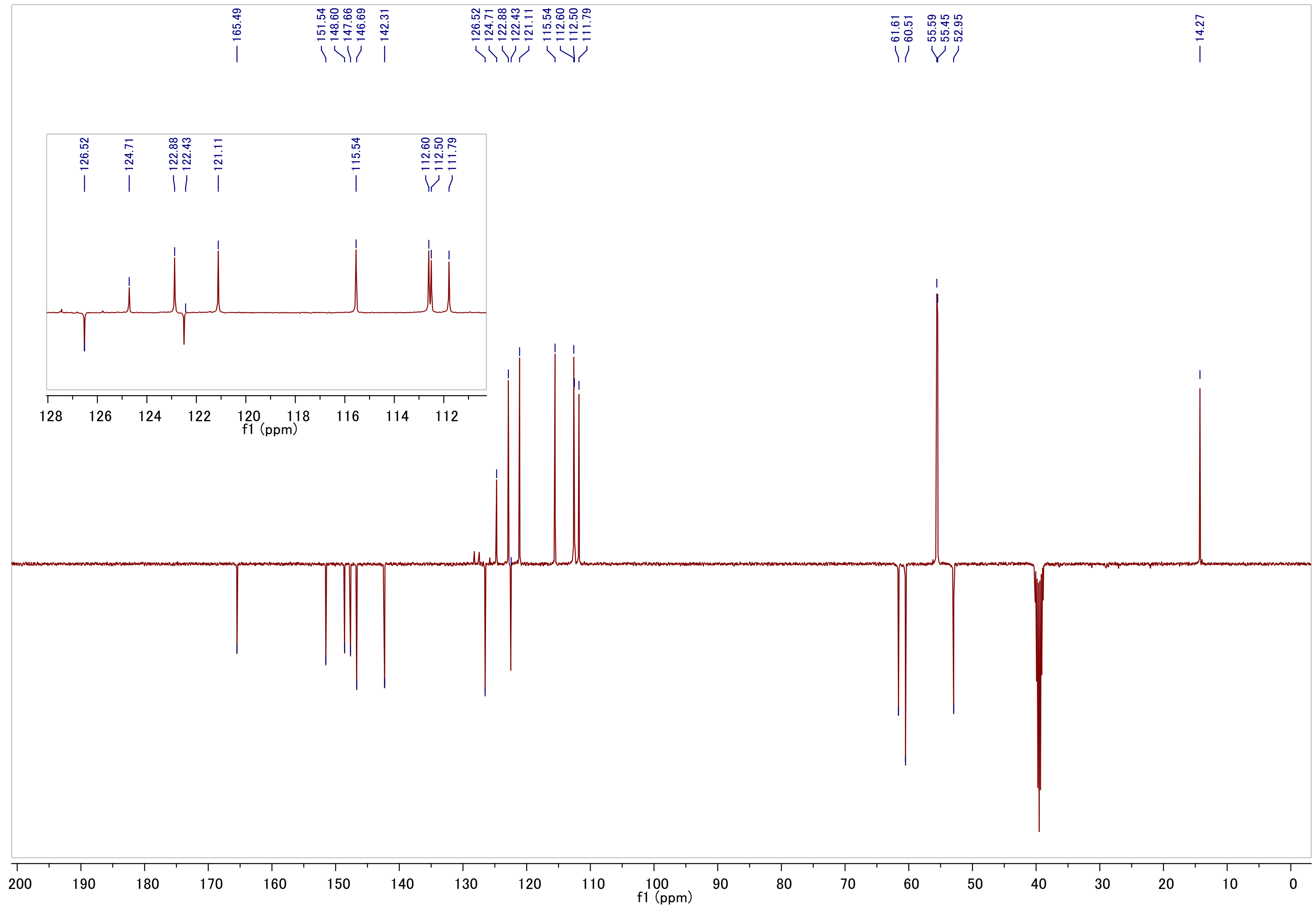

Figure S16: APT spectrum of compound 4 (DMSO-*d6*, 100 MHz).

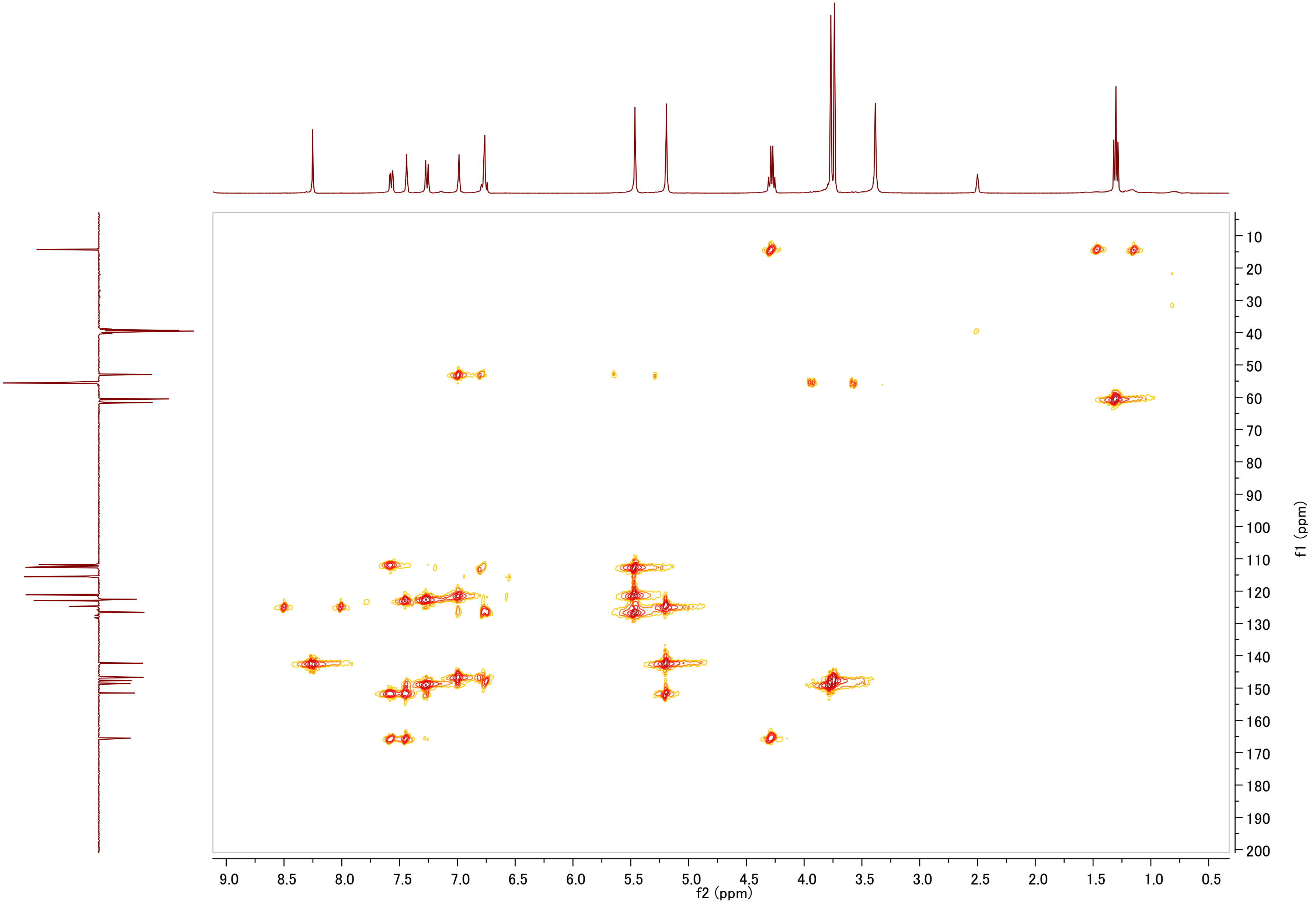

Figure S17: HMBC of compound 4 (DMSO-*d6*, 100 MHz).

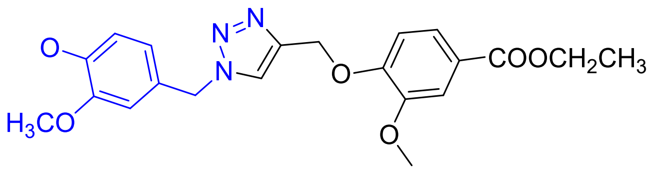

Chemical Formula: $C_{21}H_{22}N_3O_6$
Exact Mass: 412.1509

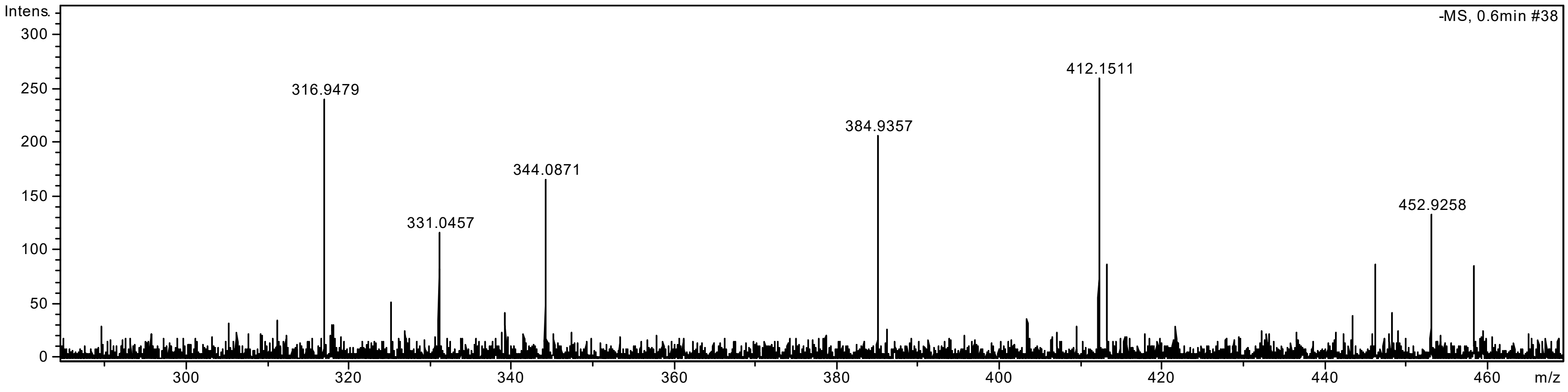

Figure S18: Negative HRESI-Mass of compound 4

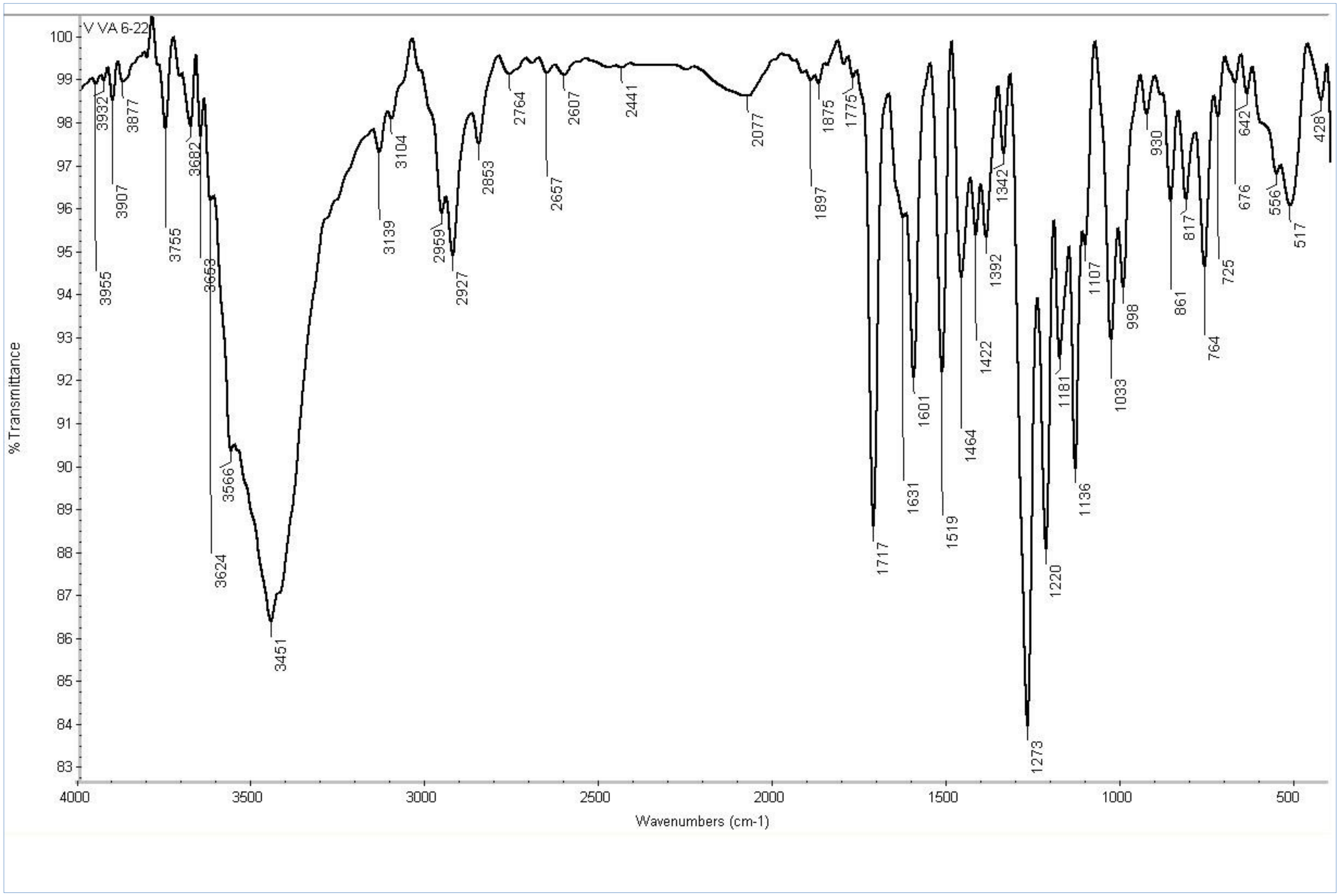

Figure S19: IR spectrum of compound 4.

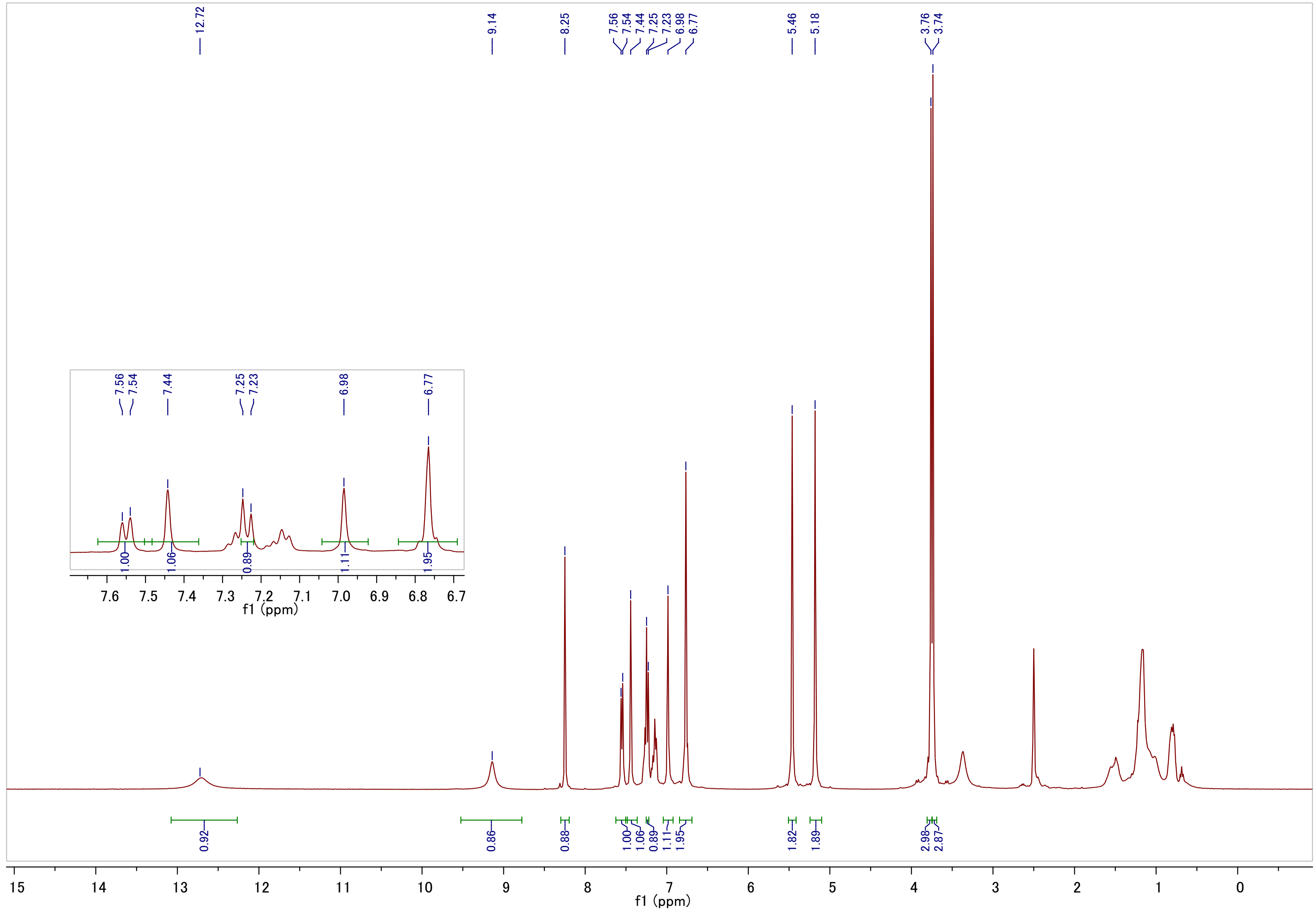

Figure S20: ^{1}H-NMR spectrum of compound 5 (DMSO-*d6*, 400 MHz).

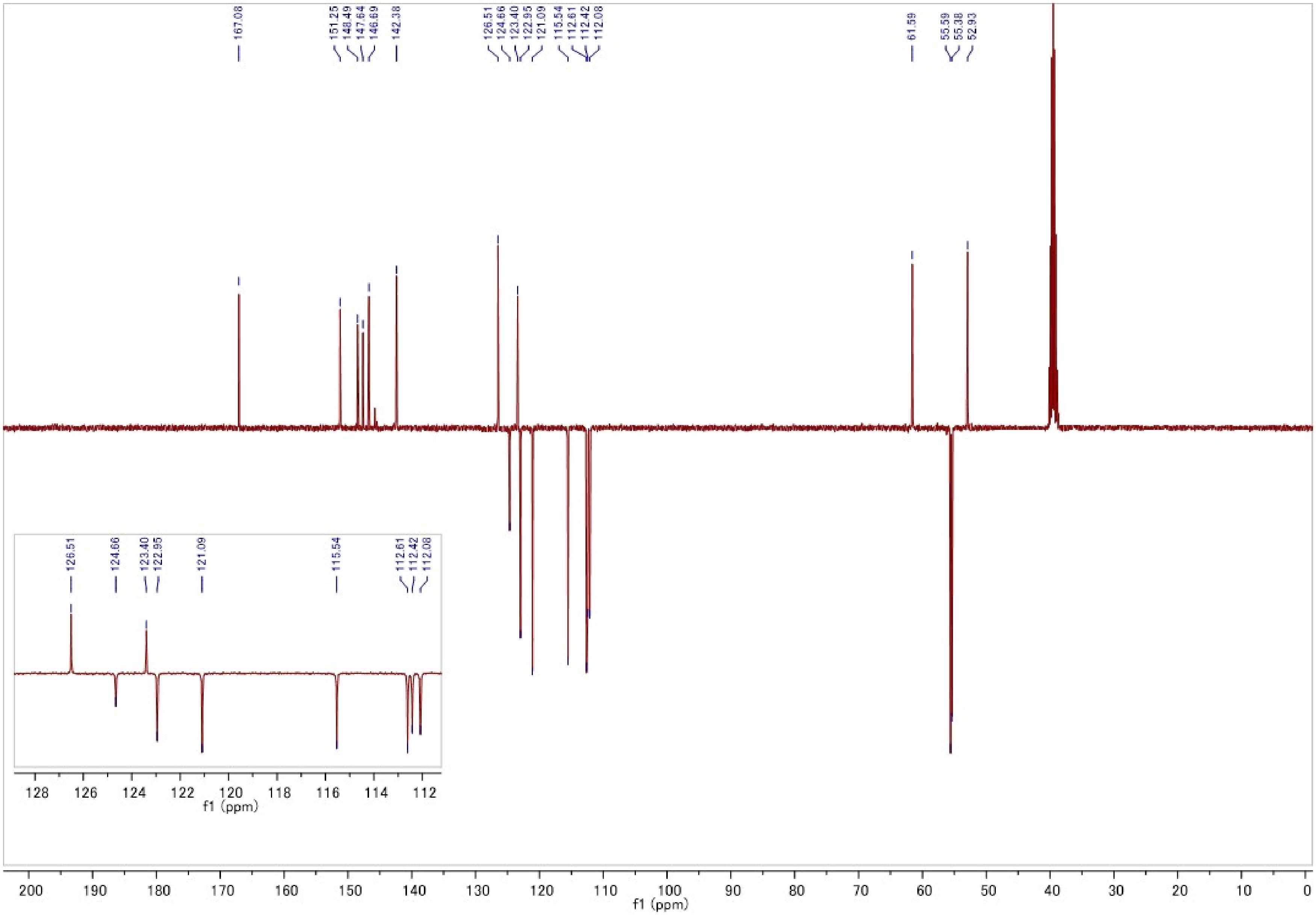

Figure 21: APT spectrum of compound 5 (DMSO-*d6*, 100 MHz).

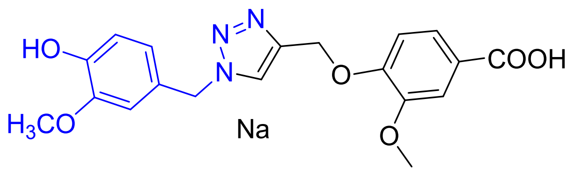

Chemical Formula: $C_{19}H_{19}N_3NaO_6Na$
Exact Mass:408.1172

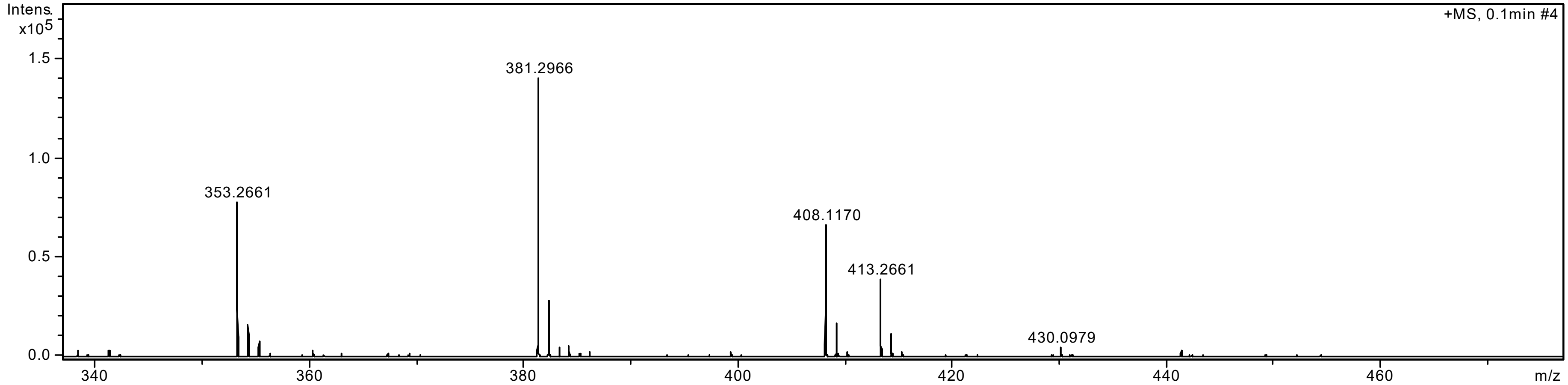

Figure S22: Positive HRESI-MS of compound 5.

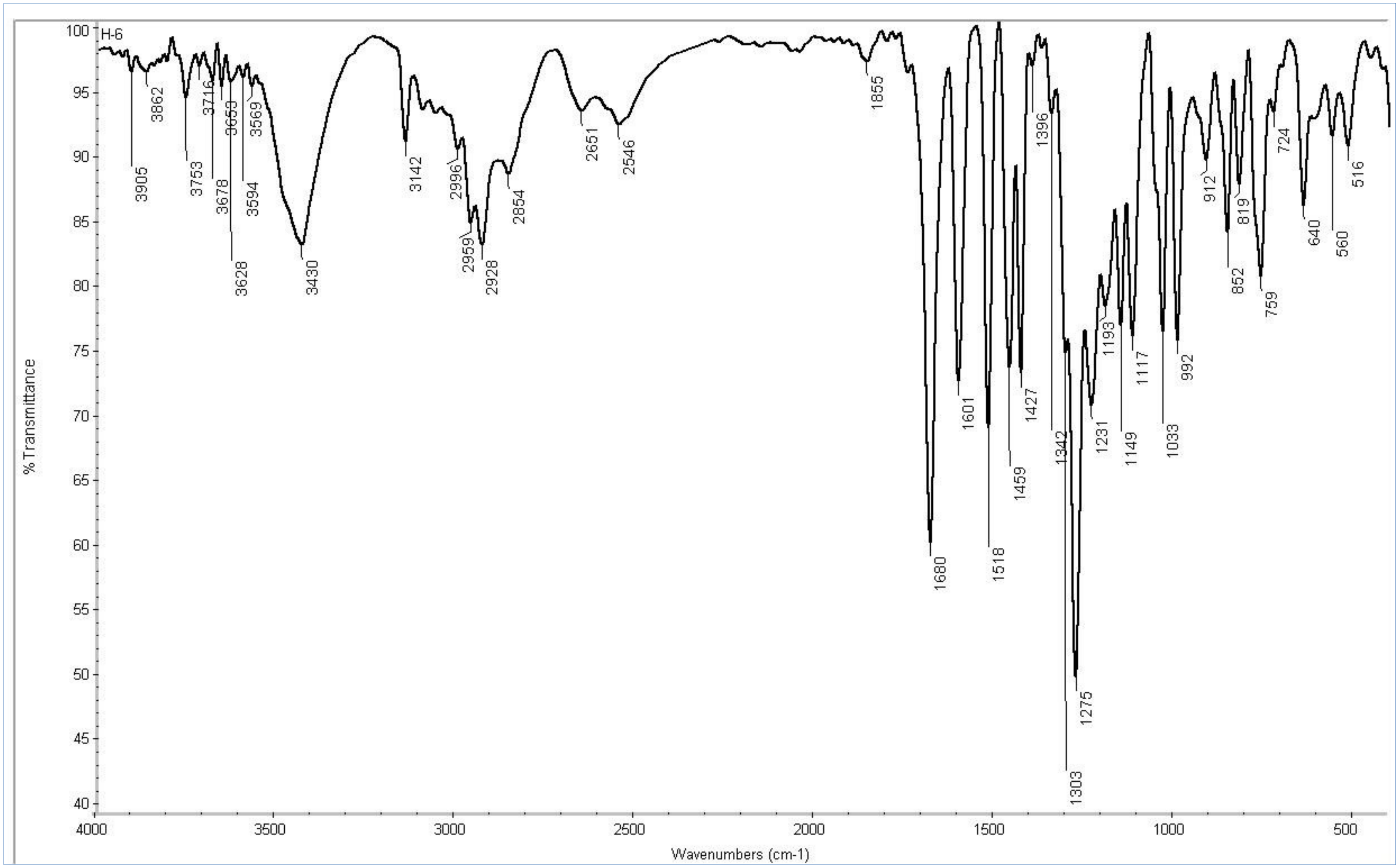

Figure S23: IR spectrum of compound 5.

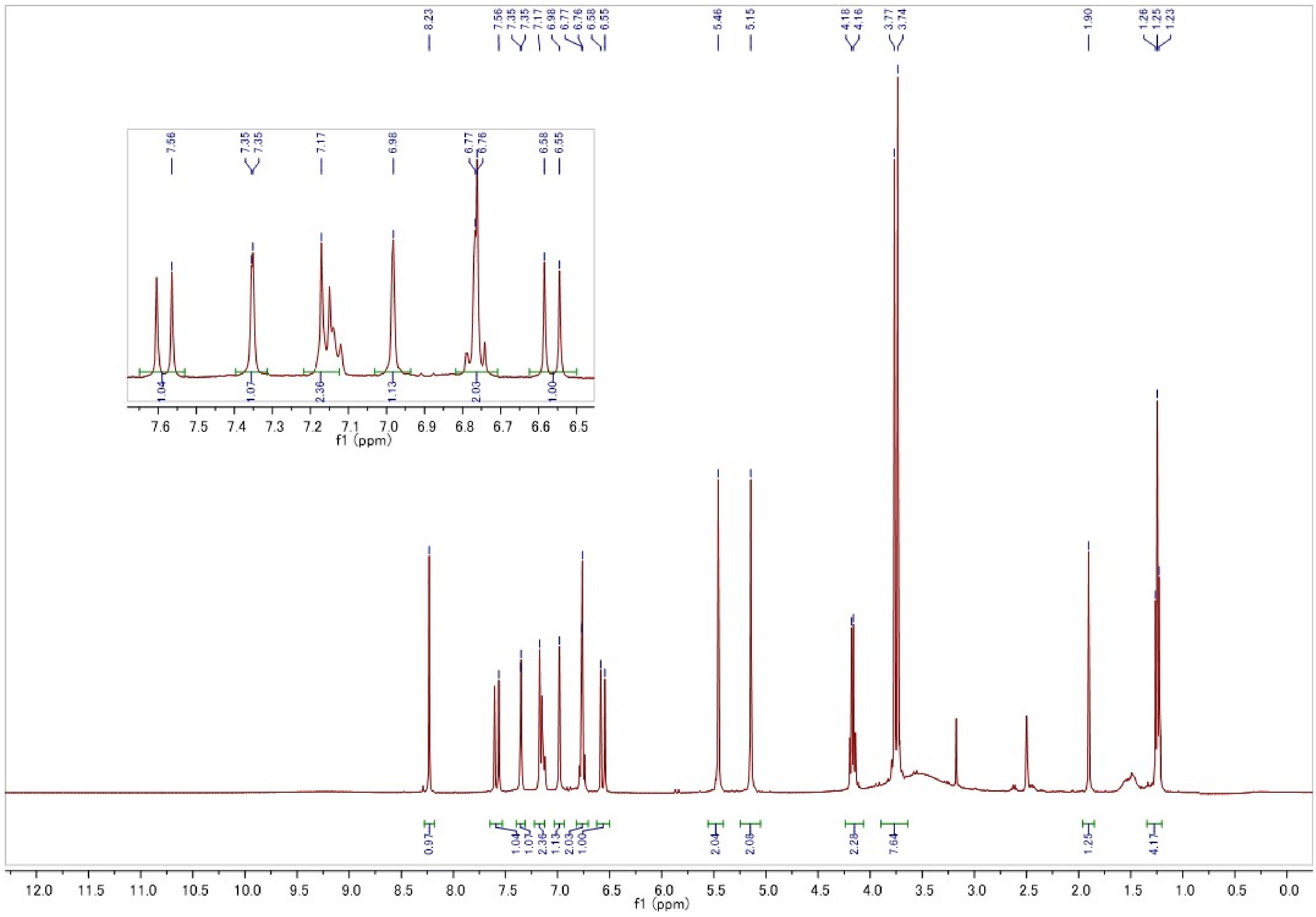

Figure S24: ^{1}H-NMR spectrum of compound 6 (DMSO-*d6*, 400 MHz).

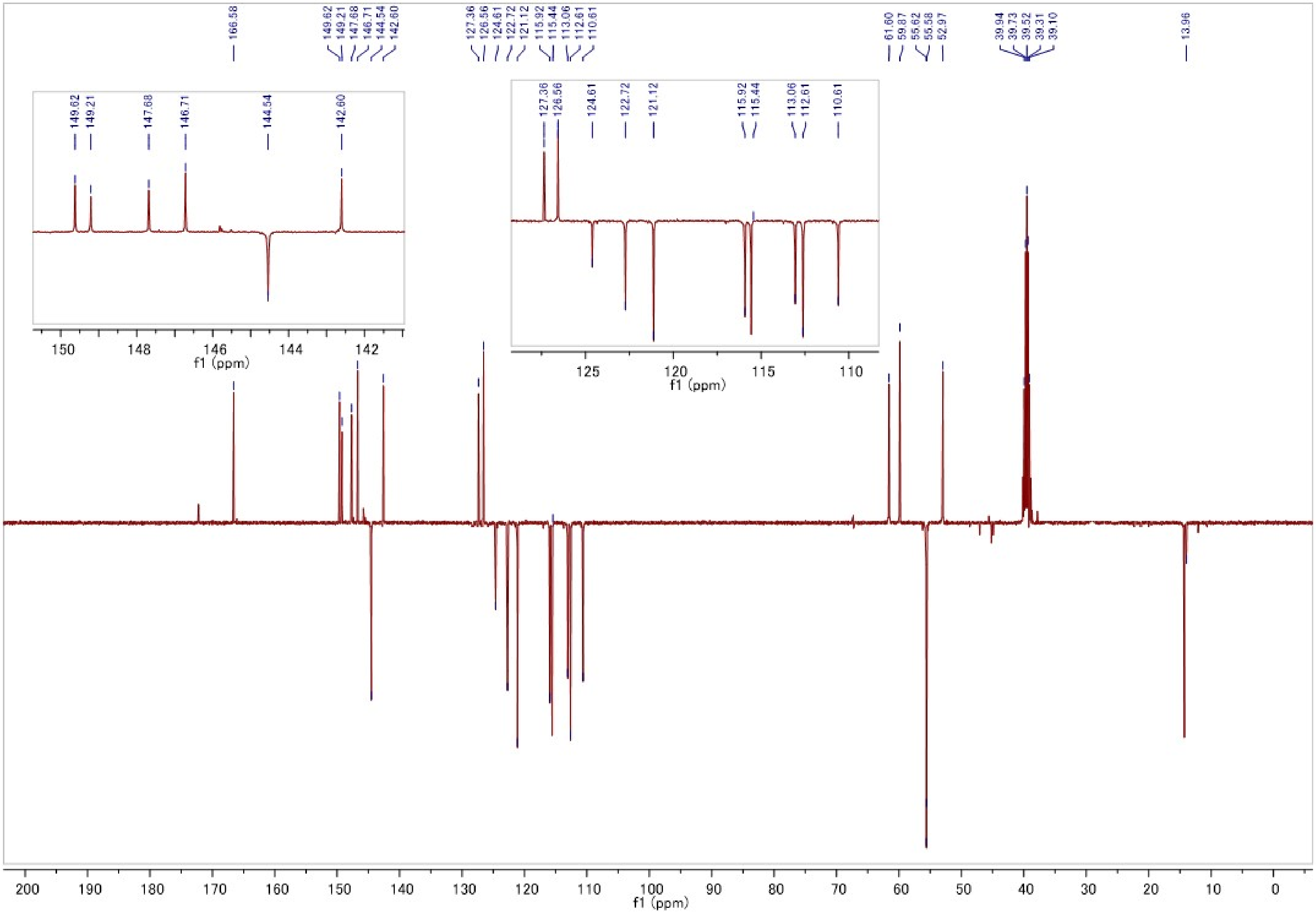

Figure S25: APT spectrum of compound 6 (DMSO-*d6*, 100 MHz).

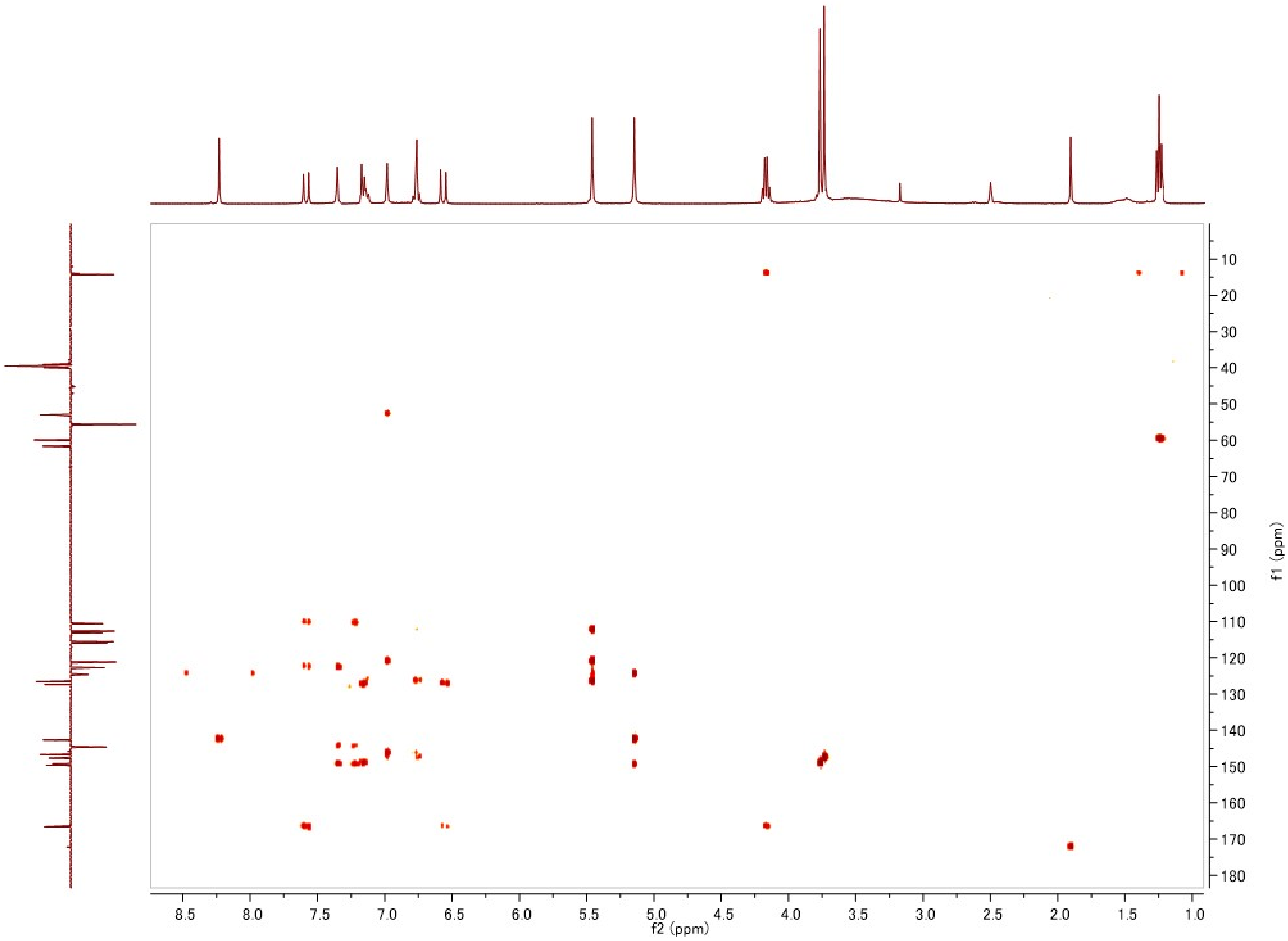

Figure S26: HMBC of compound 6 (DMSO-*d6*, 100 MHz).

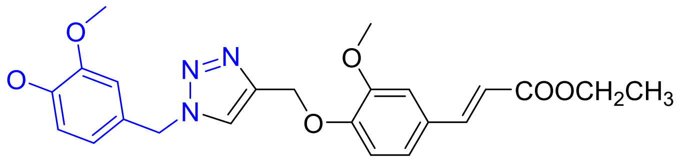

Chemical Formula: $C_{23}H_{24}N_3O_6$
Exact Mass: 438.1665

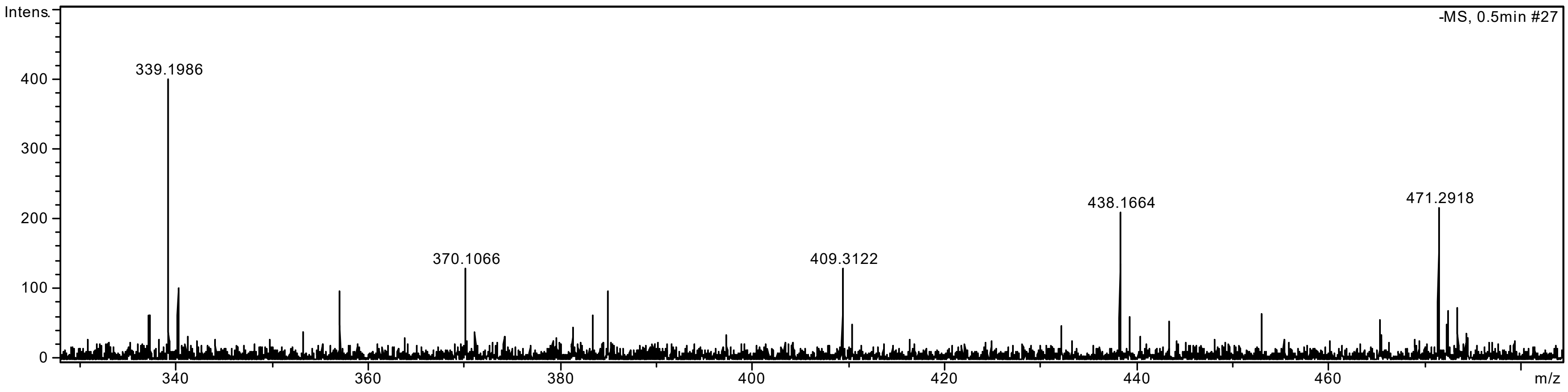

Figure S27: Negative HRESI-MS of compound 6.

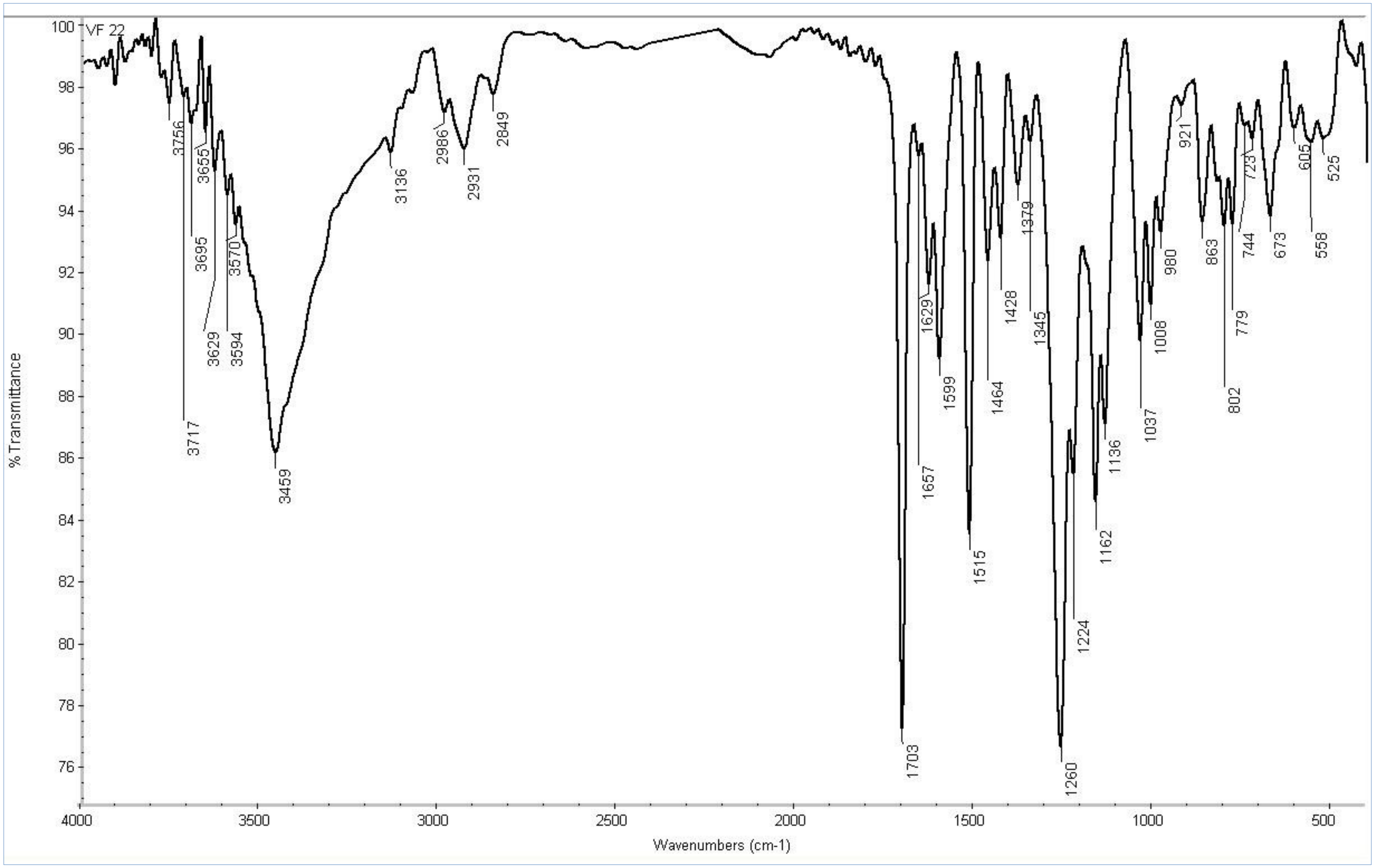

Figure S28: IR spectrum of compound 6.

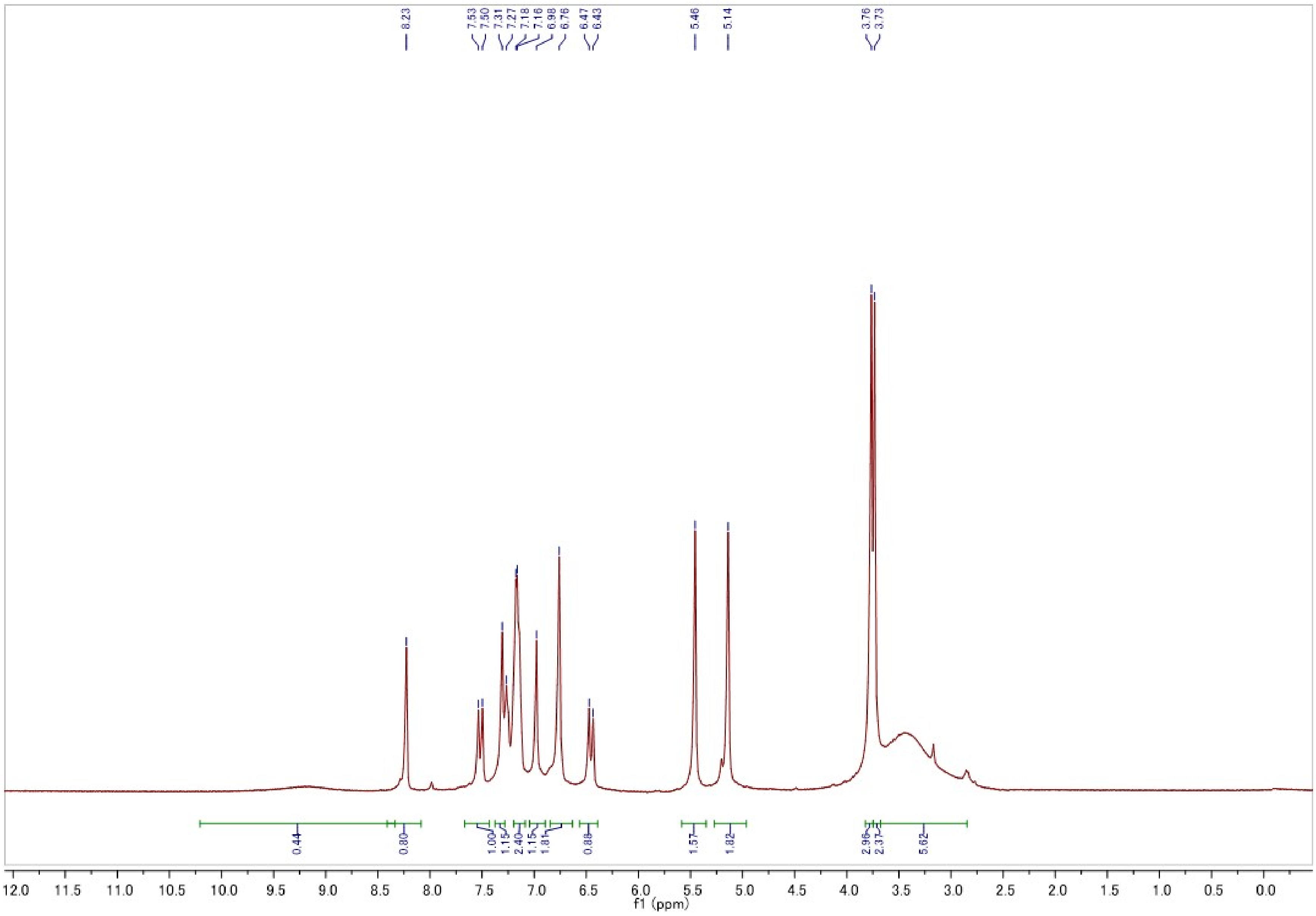

Figure S29: ^{1}H-NMR spectrum of compound 7 (DMSO-*d6*, 400 MHz).

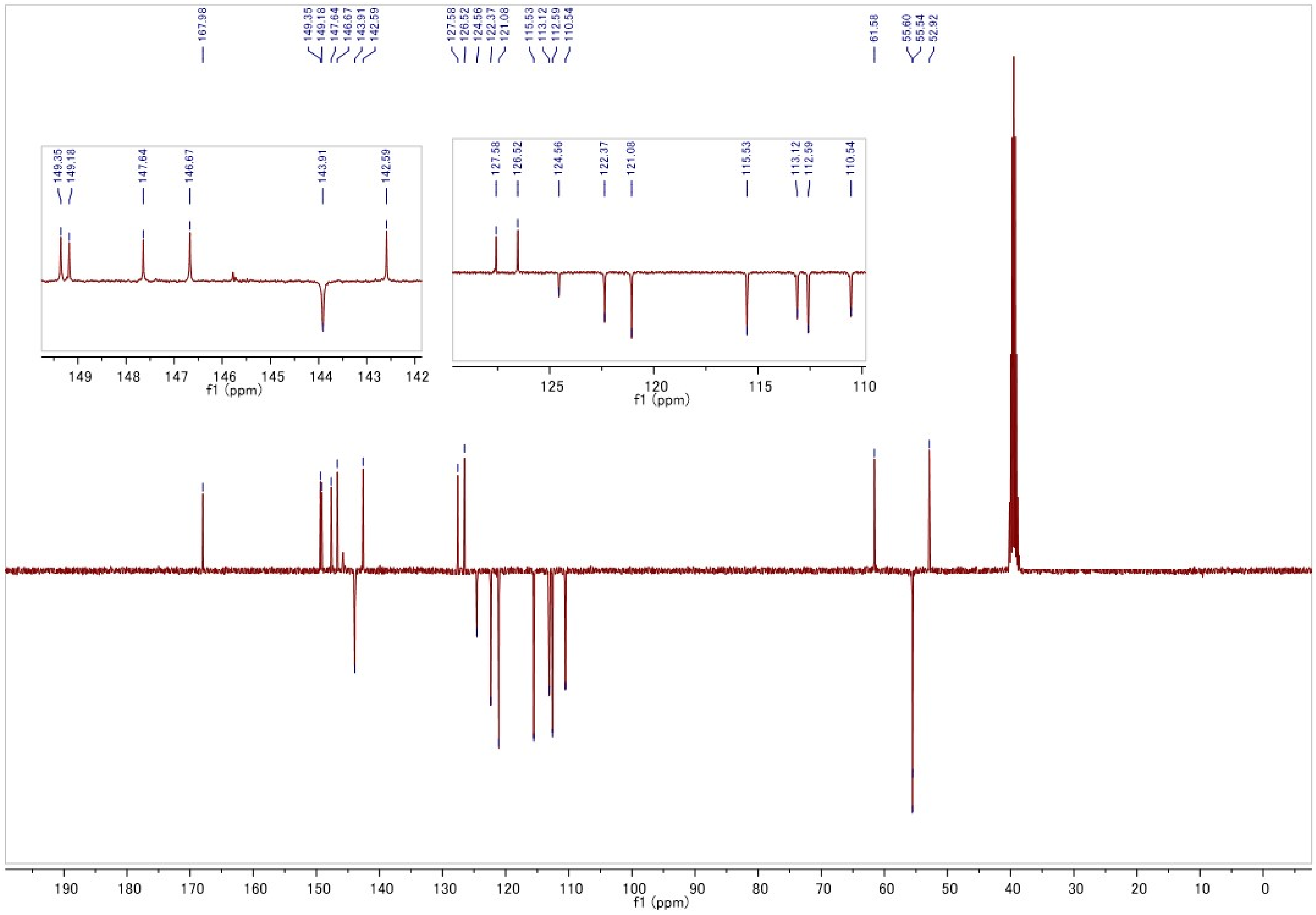

Figure S30: APT spectrum of compound 7 (DMSO-*d6*, 100 MHz).

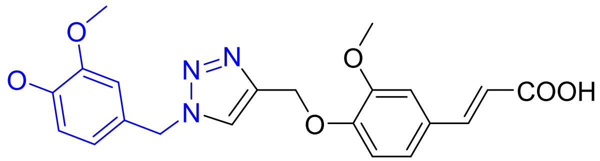

Chemical Formula: $C_{21}H_{20}N_3O_6$
Exact Mass: 410.1352

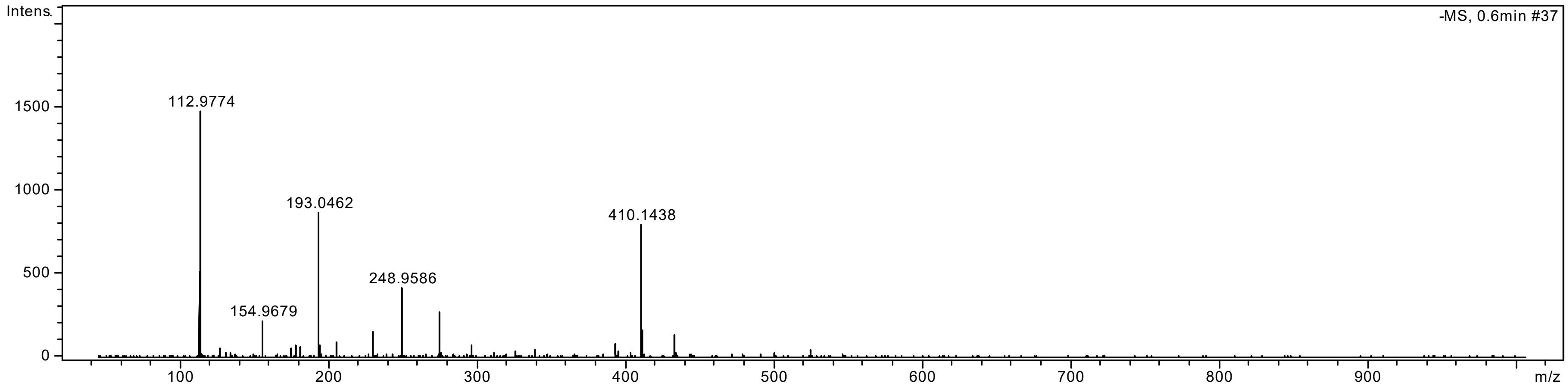

Figure S31: Negative HRESI-MS of compound 7.

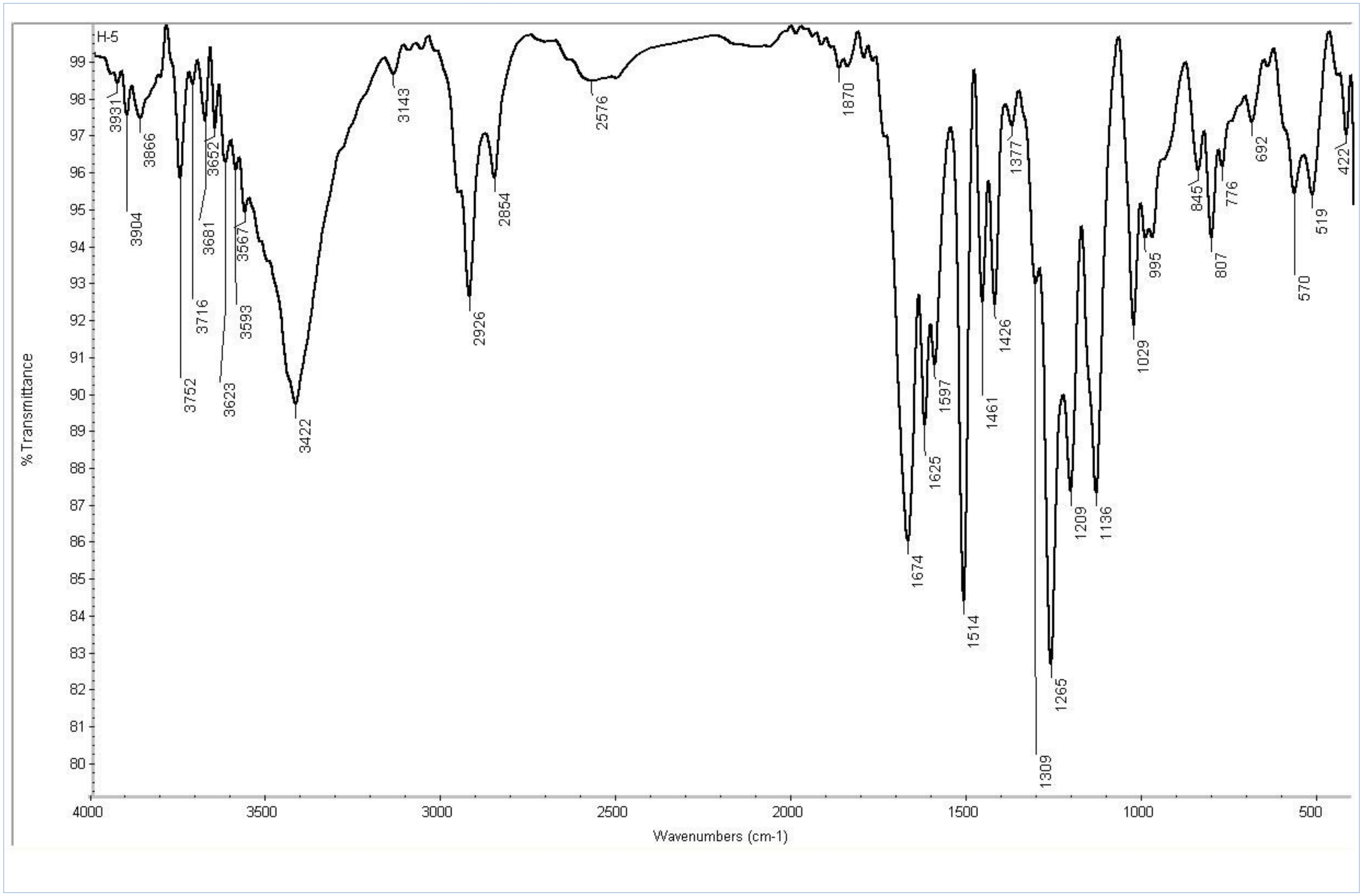

Figure S32: IR spectrum of compound 7.

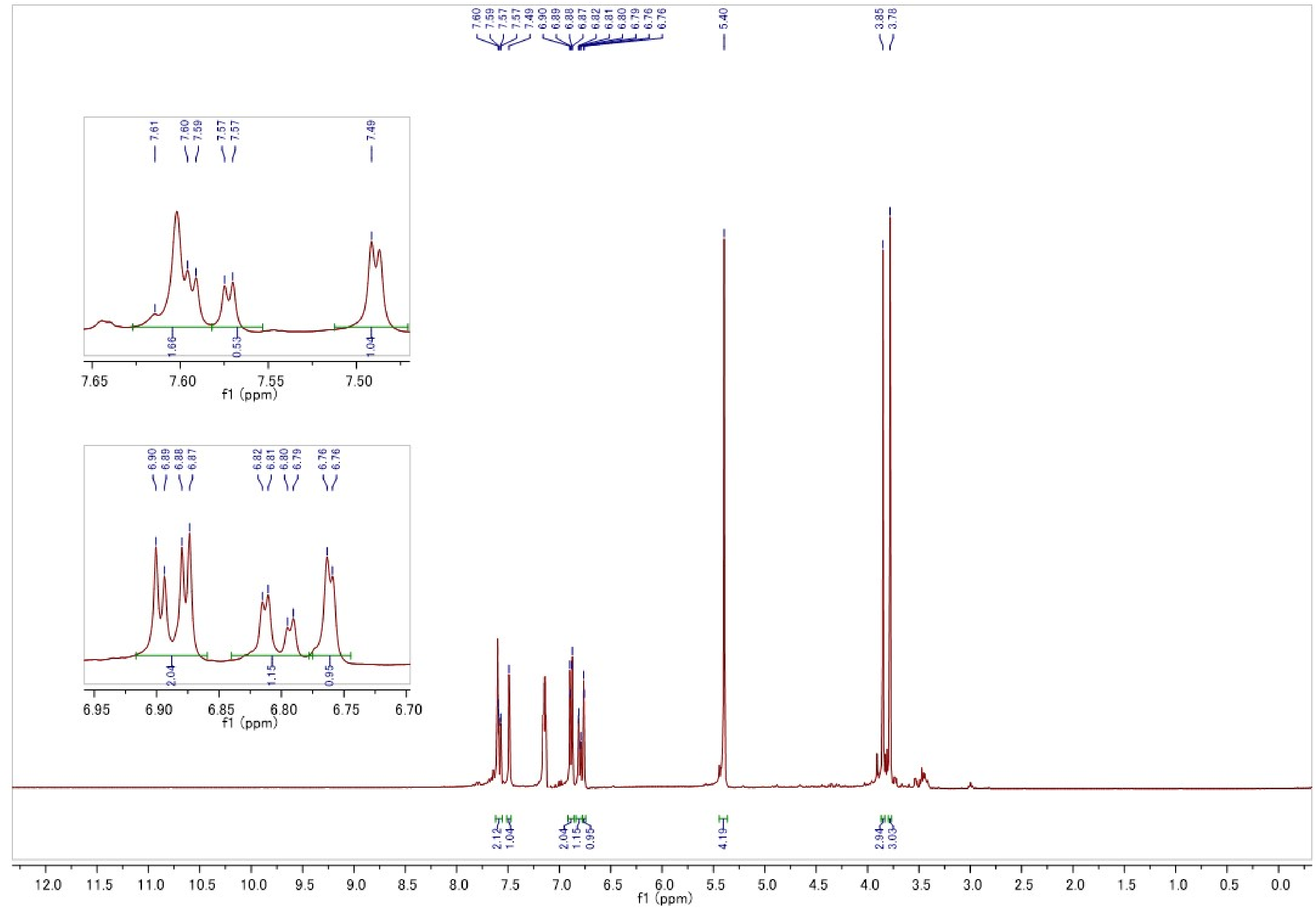

Figure S33: ^{1}H-NMR spectrum of compound 8 (DMSO-*d6*, 400 MHz).

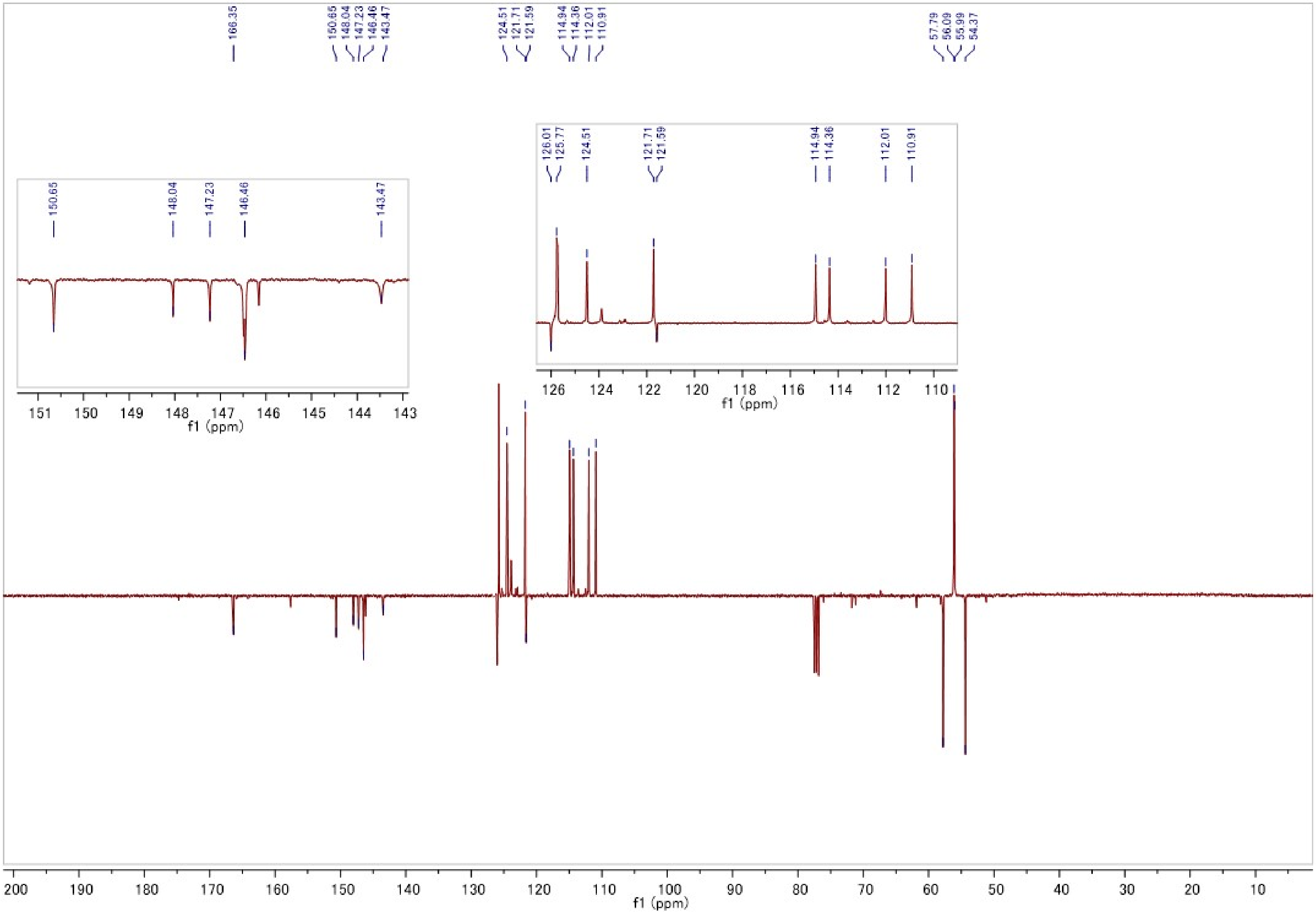

Figure S34: APT spectrum of compound 8 ($CDCl_3$, 100 MHz).

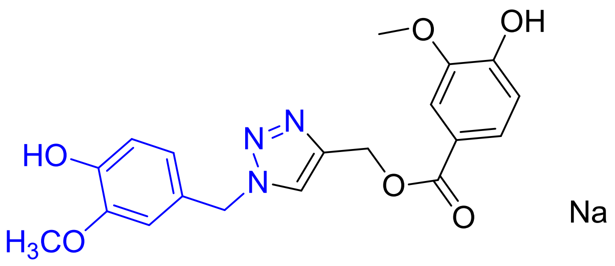

M+ Na

Chemical Formula: $C_{19}H_{19}N_3NaO_6$

Exact Mass:408.1172

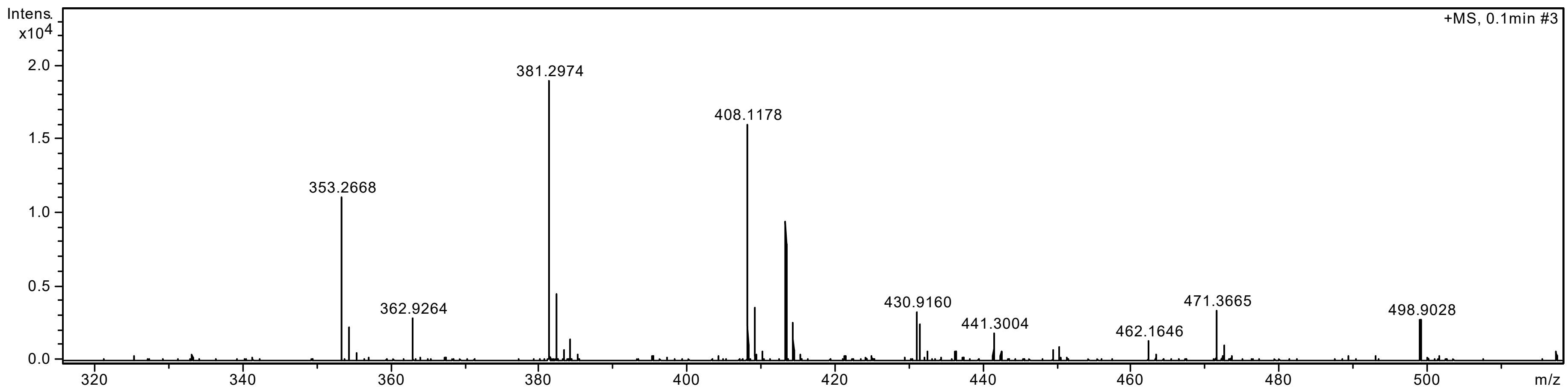

Figure S35: Positive HRESI-Mass of compound 8.

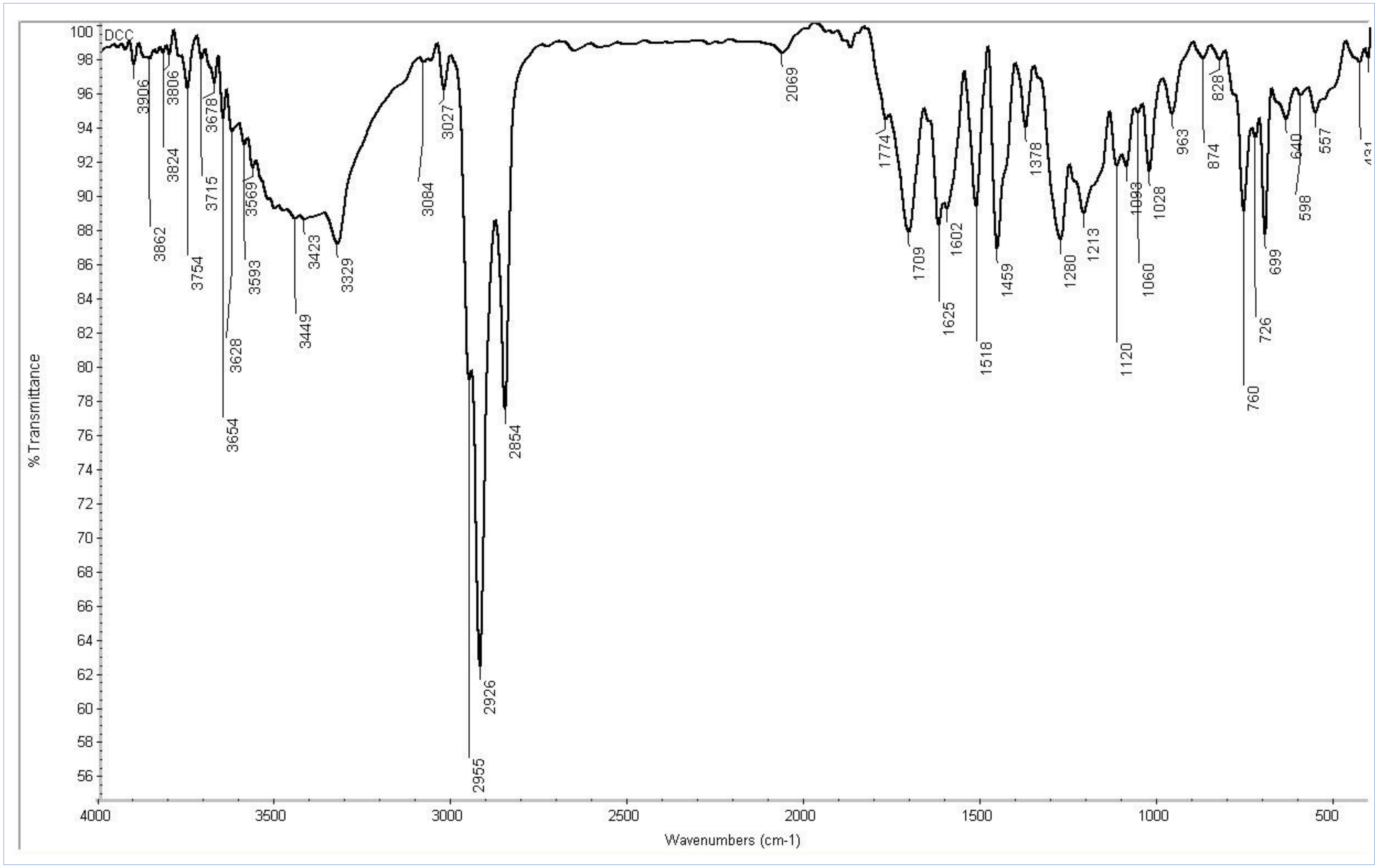

Figure S36: IR spectrum of compound 8.

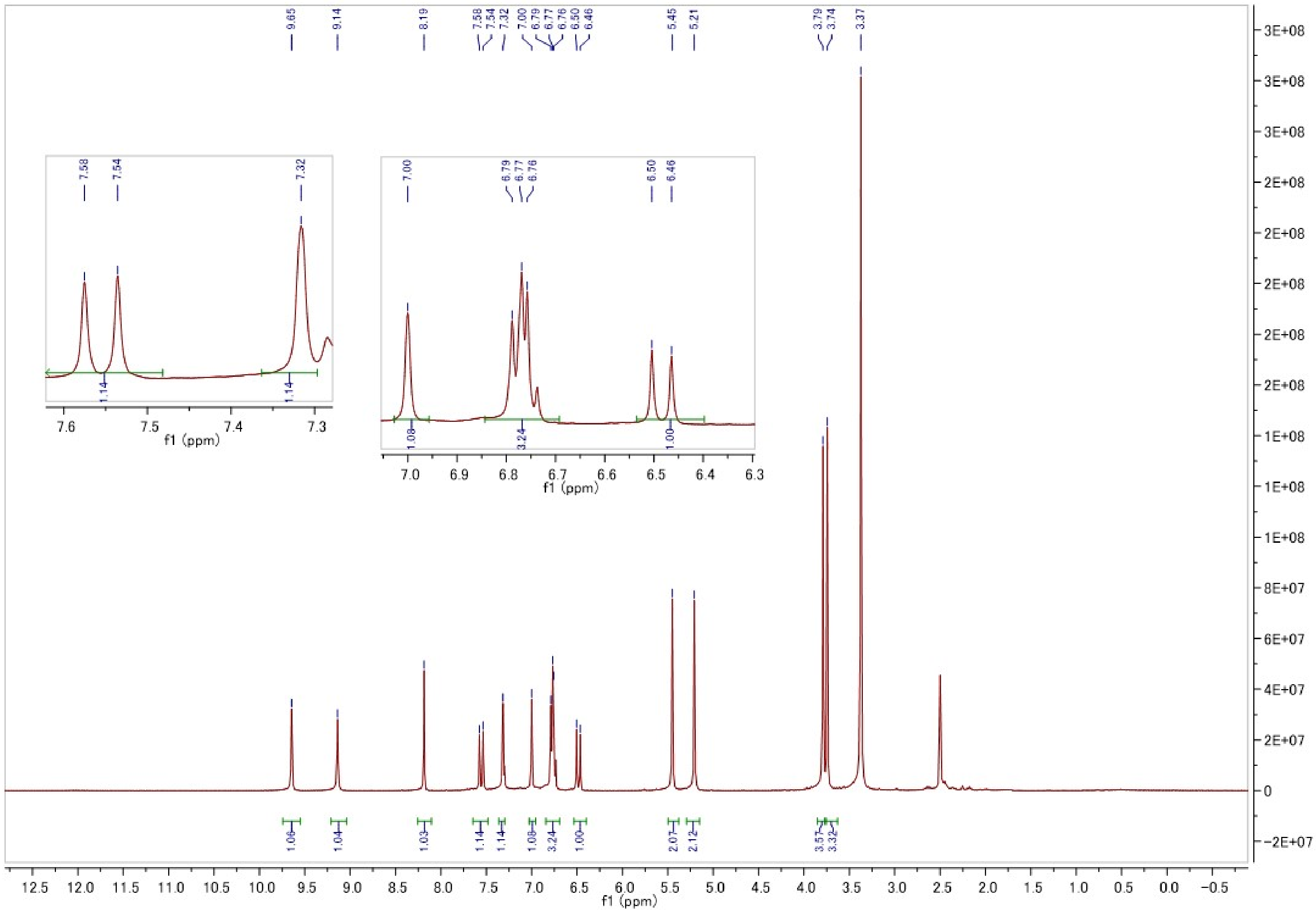

Figure S37: ^{1}H-NMR spectrum of compound 9 (DMSO-*d6*, 400 MHz).

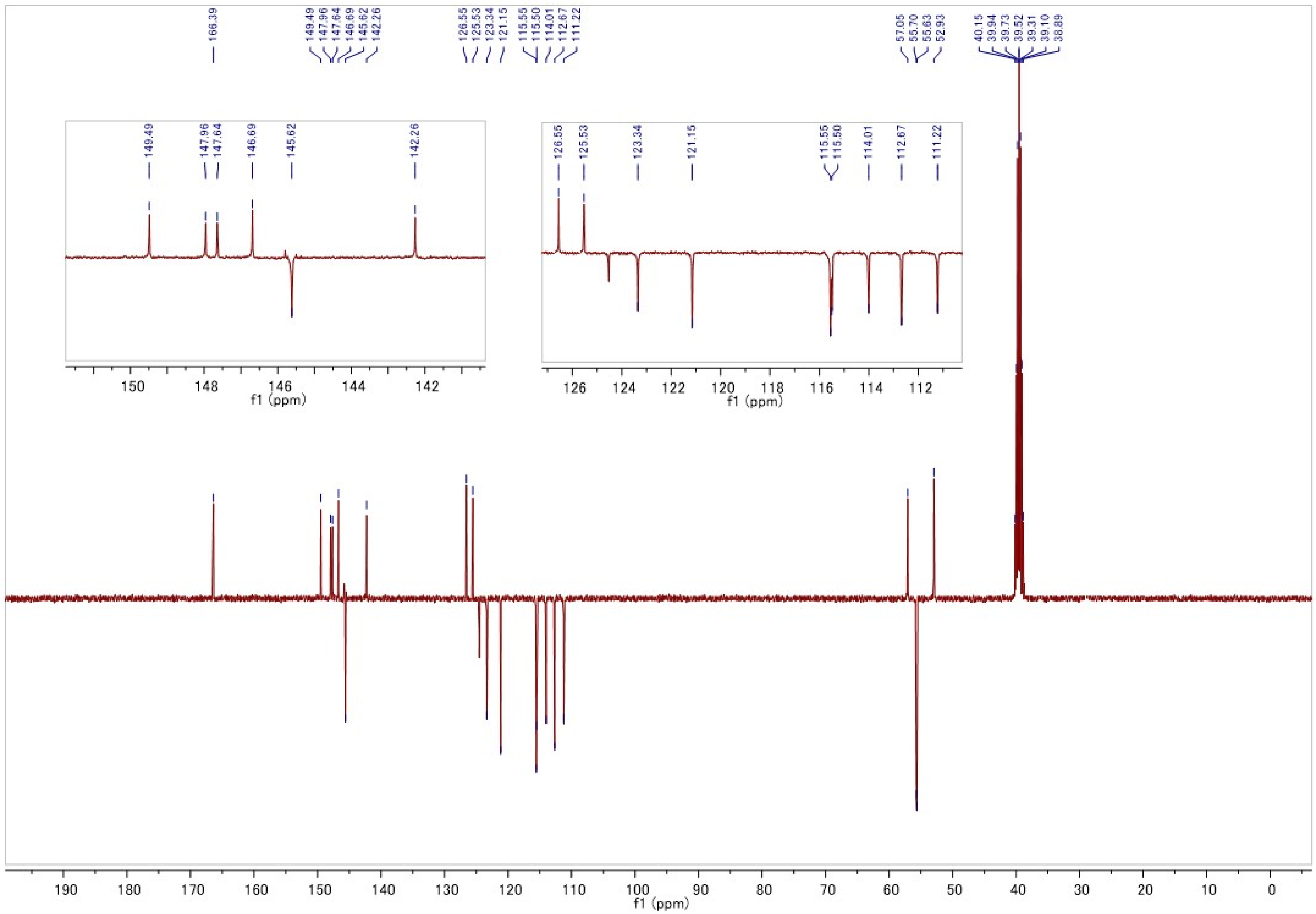

Figure S38: APT spectrum of compound 9 (DMSO-*d6*, 100 MHz).

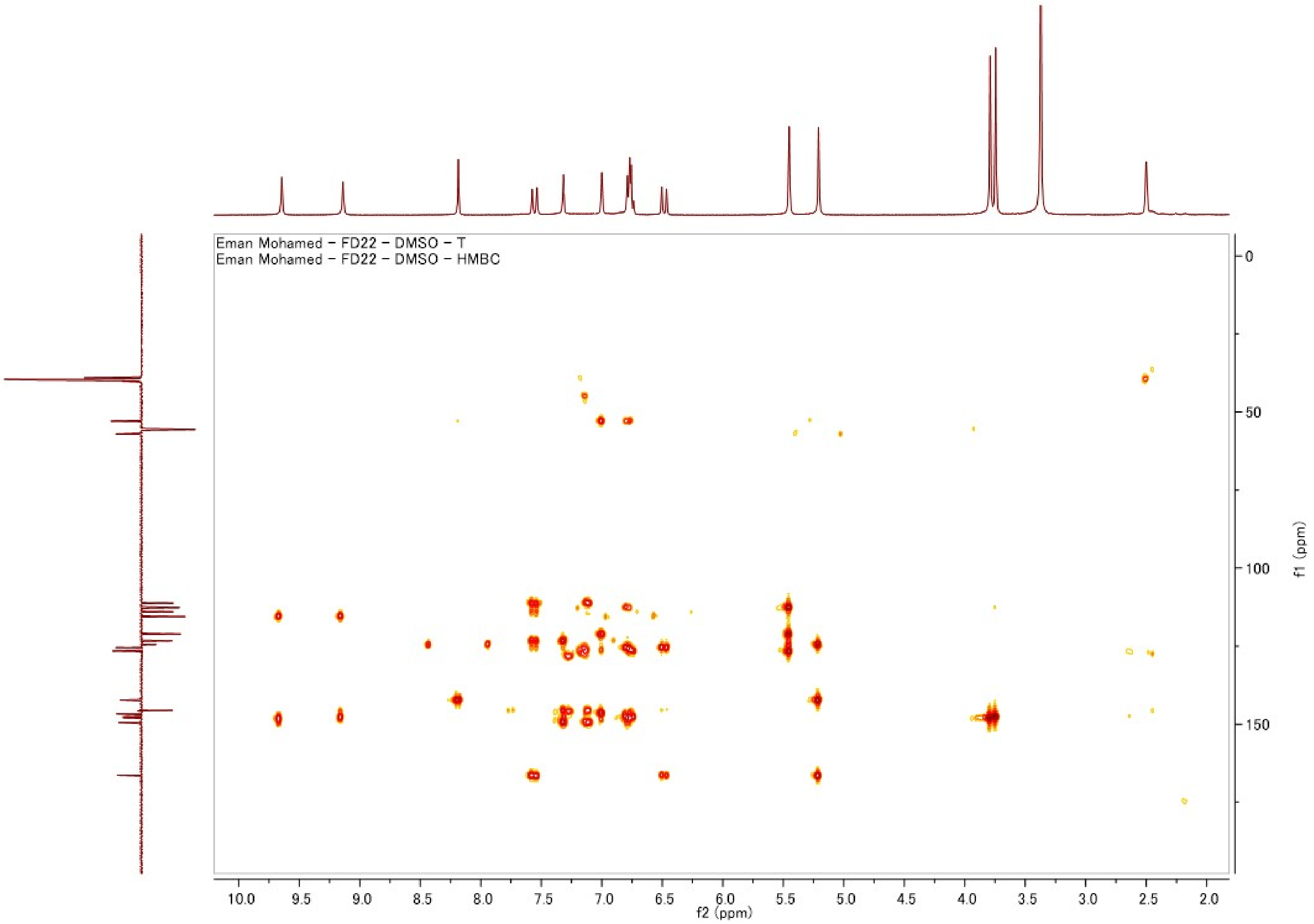

Figure S39: HMBC of compound 9 (DMSO-*d6*, 100 MHz).

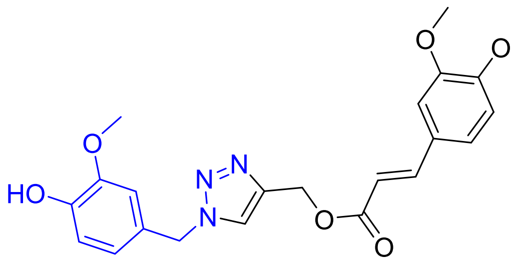

Chemical Formula: $C_{21}H_{20}N_3O_6$
Exact Mass: 410.1352

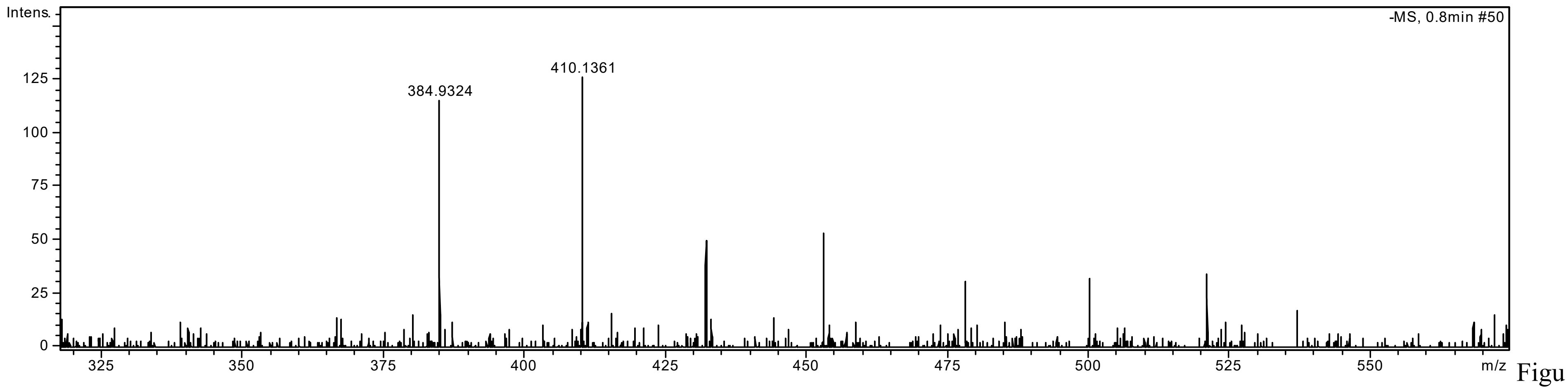

Figure S40: Negative HRESI-Mass of compound 9.

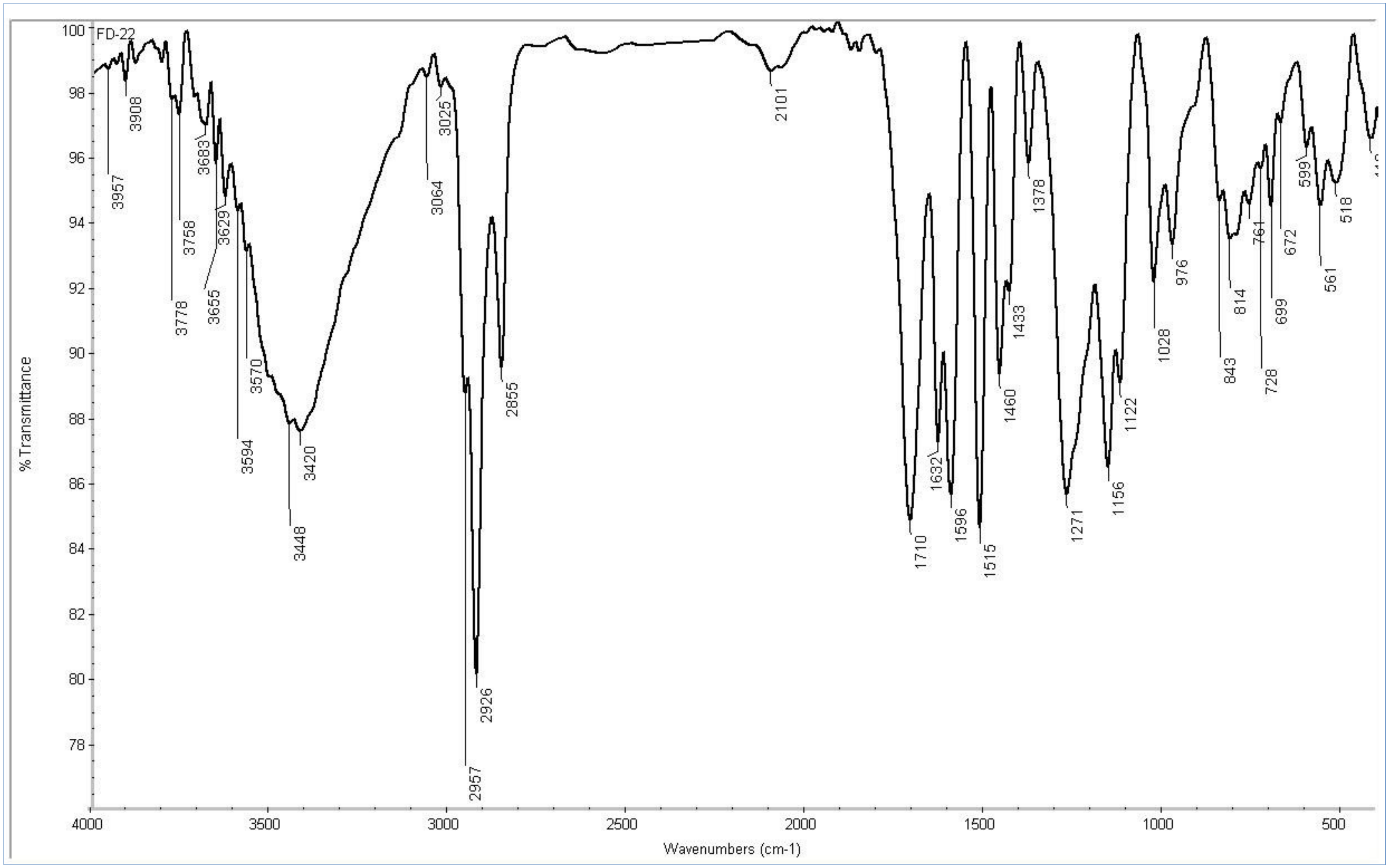

Figure S41: IR spectrum of compound 9.

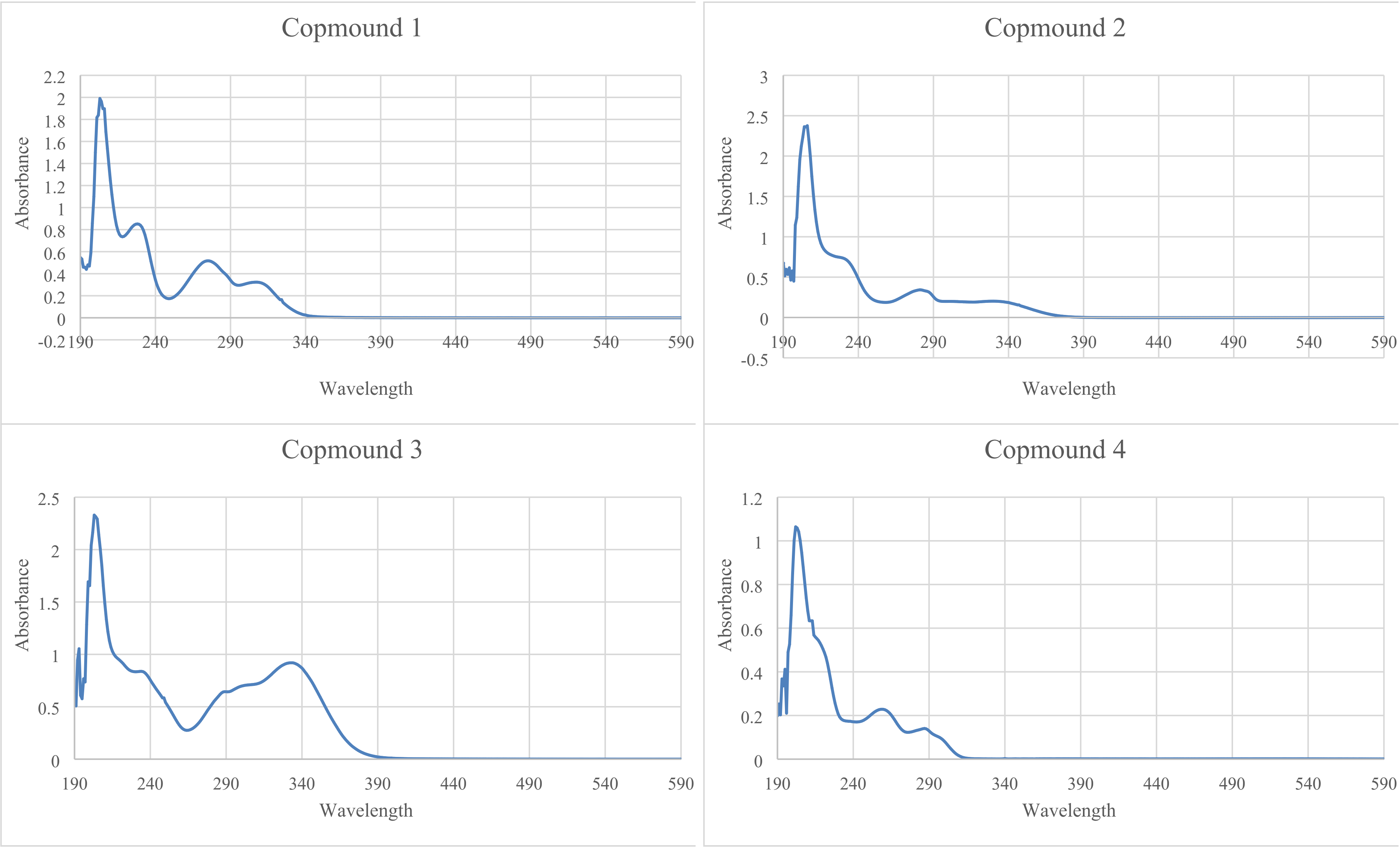

Figure S42: UV spectrum of compounds 1-4

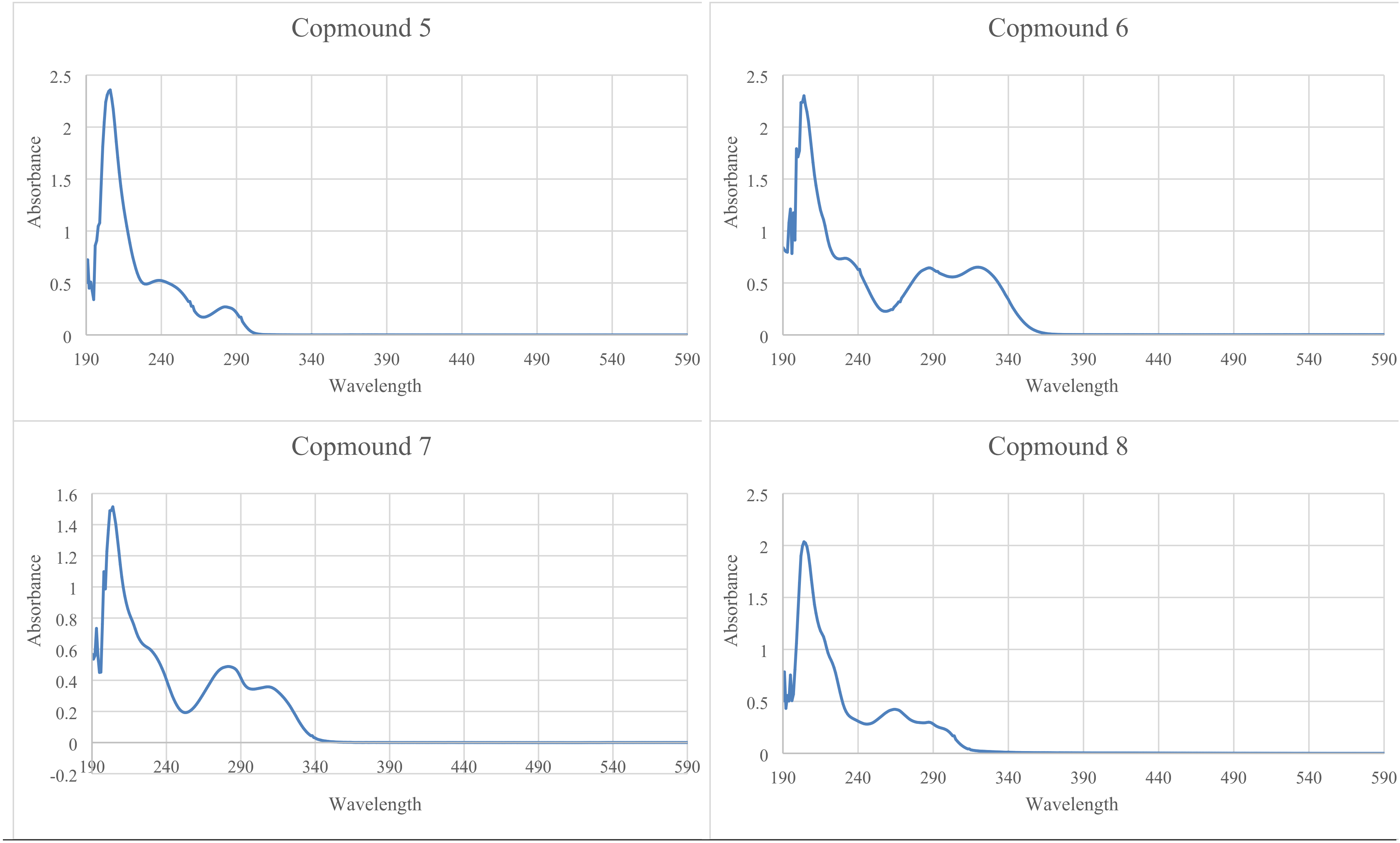

Figure S43: UV spectrum of compounds 5-8

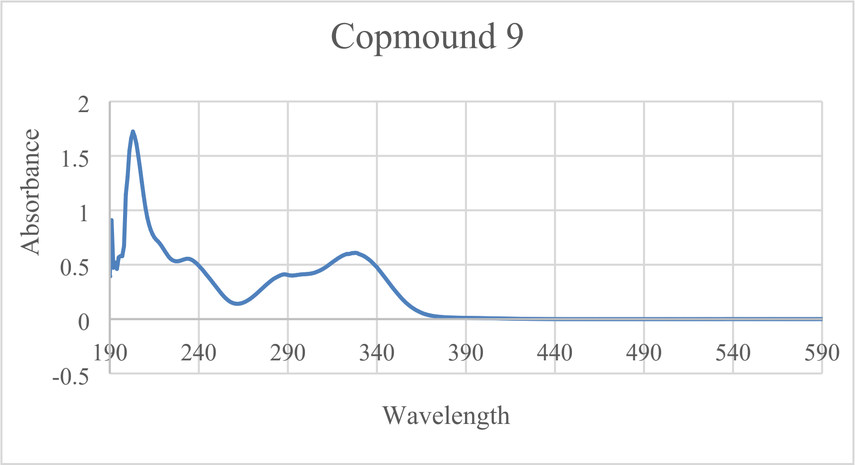

Figure S44: UV spectrum of compound 9.

Biological evaluation

1. AChE inhibition assay

The synthesized compounds were assayed to determine their ability to inhibit hAChE using (K197-100, Bio Vision, Egypt)[1]. A 96-well plate was used by applying the spectroscopic method of Ellman[1], and donepezil as positive reference compound. The compounds were dissolved in a small amount of DMSO to obtain a concentration range of 0.01-10 μM. Each well contained 160 μl of dithiobis-(2-nitrobenzoic acid) (Ellman's reagent, DTNB), 10 μl of the tested compound and 50 μl hAChE solution, while Blank wells contained 160 μl DTNB, 10 μl DMSO, and 50 μl buffer (1 M, pH=8.0, Tris-HCl) . 100% ChE activity (100% activity) contained 160 μl DTNB, 2 μl DMSO, 8 μl buffer and 50 μl hAChE. After 5 min of incubation at 25 °C, 30 μl of acetylthiocholine iodide (ATC) 15 μM was then added to obtain a final volume of 250 μl. Absorbance readings at 412 nm were taken for 40 min. Two time points (t1 and t2) in the linear range of the plot were chosen and the corresponding values for the absorbance (OD1 and OD2) were obtained. The percentage of relative inhibition was calculated from the following equation:

% Relative Inhibition = Slope of[EC]−Slope of [S] /Slope of[EC] X100
% Relative Activity = Slope of [S] /Slope of [EC] X100
Where: S, is Sample Screen; EC, is Enzyme Control.
IC_{50} was determined graphically by plotting the values of relative activity percentage against their corresponding logarithmic concentration, best fitted line was determined and IC_{50} was calculated from its equation.

2. *In Vitro* Amyloid-β Aggregation Assay

Amyloid-β (1–42) aggregation was measured using the SensoLyte® Thioflavin T β-Amyloid aggregation kit (AnaSpec, Fremont, CA, USA)[2], according to the manufacturer's instructions. The assay is based on the property of the Thioflavin T dye, the fluorescence of which increases when it is bound to aggregates of amyloid-β (1–42) peptides. Briefly, Thioflavin T was dissolved in assay buffer [10 mM Phosphate/150 mM NaCl (pH=8.0)] and used at a concentration of 100 μM. Samples were dissolved in assay buffer, 5 μL of the sample and 85 μL of amyloid-β (1–42) were mixed, followed by the addition of 10 μL of 2mM Thioflavin T. Thioflavin T fluorescence was measured at intervals of 10 min for 2 h, with an excitation /emission =440 nm/484 nm wavelength (λ_{ex}). All fluorescence readings are expressed in relative fluorescence units (RFU). Morin was used as a positive control. Experiments were performed in triplicate and averaged, and the percentage of inhibition of amyloid-β aggregation was calculated according to the following equation:

% Relative Inhibition = (RFU of PC - RFU of S)/ (RFU of S) x 100

Where: PC, is the Peptide Positive Control or Solvent Control; S, is Sample Screen.

IC_{50} was generated by plotting the values of percent inhibition against their corresponding logarithmic concentration. Best fitted line was determined and IC_{50} was calculated from its equation.

References:
1. G.L. Ellman, K.D. Courtney, V. Andres Jr., R.M. Featherstone, *Biochem. Pharmacol.* 1961, **7**, 88e95.

2. Hellstrand E., et. al., ACS Chem. Neurosci., 2010, **1**, 13-18.
